# Integrated Real-World Study Databases in 3 Diverse Asian Health Care Systems in Taiwan, India, and Thailand: Scoping Review

**DOI:** 10.2196/49593

**Published:** 2023-09-11

**Authors:** Wen-Yi Shau, Sajita Setia, Ying-Jan Chen, Tsu-yun Ho, Salil Prakash Shinde, Handoko Santoso, Daniel Furtner

**Affiliations:** 1 Regional Medical Affairs Pfizer Corporation Hong Kong Limited Hong Kong Hong Kong; 2 Executive Office Transform Medical Communications Limited Wanganui New Zealand; 3 Department of Oncology National Taiwan University Hospital Taipei Taiwan; 4 Medical Affairs Office National Taiwan University Cancer Center Taipei Taiwan

**Keywords:** Asia, health care databases, real-world data, real-world evidence, scoping review

## Abstract

**Background:**

The use of real-world data (RWD) warehouses for research in Asia is on the rise, but current trends remain largely unexplored. Given the varied economic and health care landscapes in different Asian countries, understanding these trends can offer valuable insights.

**Objective:**

We sought to discern the contemporary landscape of linked RWD warehouses and explore their trends and patterns in 3 Asian countries with contrasting economies and health care systems: Taiwan, India, and Thailand.

**Methods:**

Using a systematic scoping review methodology, we conducted an exhaustive literature search on PubMed with filters for the English language and the past 5 years. The search combined Medical Subject Heading terms and specific keywords. Studies were screened against strict eligibility criteria to identify eligible studies using RWD databases from more than one health care facility in at least 1 of the 3 target countries.

**Results:**

Our search yielded 2277 studies, of which 833 (36.6%) met our criteria. Overall, single-country studies (SCS) dominated at 89.4% (n=745), with cross-country collaboration studies (CCCS) being at 10.6% (n=88). However, the country-wise breakdown showed that of all the SCS, 623 (83.6%) were from Taiwan, 81 (10.9%) from India, and 41 (5.5%) from Thailand. Among the total studies conducted in each country, India at 39.1% (n=133) and Thailand at 43.1% (n=72) had a significantly higher percentage of CCCS compared to Taiwan at 7.6% (n=51). Over a 5-year span from 2017 to 2022, India and Thailand experienced an annual increase in RWD studies by approximately 18.2% and 13.8%, respectively, while Taiwan’s contributions remained consistent. Comparative effectiveness research (CER) was predominant in Taiwan (n=410, or 65.8% of SCS) but less common in India (n=12, or 14.8% of SCS) and Thailand (n=11, or 26.8% of SCS). CER percentages in CCCS were similar across the 3 countries, ranging from 19.2% (n=10) to 29% (n=9). The type of RWD source also varied significantly across countries, with India demonstrating a high reliance on electronic medical records or electronic health records at 55.6% (n=45) of SCS and Taiwan showing an increasing trend in their use over the period. Registries were used in 26 (83.9%) CCCS and 31 (75.6%) SCS from Thailand but in <50% of SCS from Taiwan and India. Health insurance/administrative claims data were used in most of the SCS from Taiwan (n=458, 73.5%). There was a consistent predominant focus on cardiology/metabolic disorders in all studies, with a noticeable increase in oncology and infectious disease research from 2017 to 2022.

**Conclusions:**

This review provides a comprehensive understanding of the evolving landscape of RWD research in Taiwan, India, and Thailand. The observed differences and trends emphasize the unique economic, clinical, and research settings in each country, advocating for tailored strategies for leveraging RWD for future health care research and decision-making.

**International Registered Report Identifier (IRRID):**

RR2-10.2196/43741

## Introduction

The World Health Organization (WHO) has endorsed a resolution to provide universal health coverage (UHC) since 2005 [1]. UHC is defined as access to appropriate preventive, promotive, curative, and restorative services for all people at an affordable cost. Access to health care is a worldwide concern and an important element in health policies, defined as “the timely use of health services to achieve the best health outcomes” [2]. In Asia, different countries have their unique socioeconomic and health care challenges and have shown varied progress toward achieving UHC, making the region a crucial focal point for worldwide health policies [3,4].

In the context of UHC, generating and analyzing high-quality, real-world data (RWD) are crucial. RWD refers to routinely collected data relating to the health status or the delivery of health care, and real-world evidence (RWE) refers to the evidence obtained from the analysis of RWD [5]. However, using RWE to inform health technology assessment (HTA), guide health policies, and improve service delivery in the context of UHC requires an integrated, standardized, and consistent framework. Such a framework is currently absent in Asia [6]. Considering the diversity and magnitude of health challenges, Asian health systems are expected to tremendously benefit from fit-for-purpose RWE for pharmacoeconomics, pharmacovigilance, and pharmacoepidemiology assessments [6,7].

RWD warehouses provide access to large data sets when they involve large integrated health care systems with large sample sizes that increase statistical power to perform subgroup analyses and decrease the possibility of type II errors [8]. However, linkage or integration of RWD sources across health care facilities is one of the key challenges with RWE, which reduces the potential of clinical data warehouses for health care benefits and regulatory and health economic decision-making [9-11]. Nonetheless, little is known about the identity, spectrum, and scope of integrated databases, especially in Asia, which should also vary according to the health care reimbursement systems and the economies in different countries.

RWD and RWE are often the only sources of information about treatment outcomes for patients with complications, comorbidities, or other vulnerabilities, such as lifestyle factors, a high-risk family history, economic hardship, and limited access to health care [12]. Traditional randomized controlled trials often exclude or underrepresent these specific groups due to strict inclusion criteria, potential risks, or logistical challenges, thereby limiting the generalizability of trial results to these populations [13]. Appropriate use of RWE can improve access to treatment for underserved populations and impart efficiency to effective health care delivery [14]. Such evidence is of particular value for informing HTA and medical decision-making in resource-limited or lower-income countries in Asia.

To achieve the benefits of RWE in Asian health care strategy and policy, we performed a scoping review to identify and characterize the databases used for RWE generation in Asia. The complete protocol for this scoping review is described in our previous publication [15] and illustrated in Multimedia Appendix 1 (pages 2-8). As a pilot, we chose 3 countries (Taiwan, India, Thailand) with diverse health care systems and income levels defined according to World Bank data [16]. Taiwan is a high-income economy with health insurance that provides UHC and a single social health insurance scheme [15]. India is considered a lower-middle-income economy that largely relies on a self-pay health care system. Thailand, an upper-middle-income economy, has a health insurance scheme that provides UHC with differential decentralized benefit packages. Through this scoping review, we aimed to identify and characterize the research and databases used for RWE generation in these 3 Asian countries. Our hypothesis proposed that the evolving pattern of the use of RWD warehouses and RWE is influenced by the health care reimbursement system and economic status of each of the 3 countries.

## Methods

### Study Methodology

The study methodology adhered to the Preferred Reporting Items for Systematic Reviews and Meta-Analyses Extension for Scoping Reviews (PRISMA-ScR) guidelines [17]. The complete protocol has been published before [15] and is summarized infographically in Multimedia Appendix 1 (pages 6-8).

### Literature Search

We performed a comprehensive literature search to identify RWE/RWD studies in India, Thailand, and Taiwan by incorporating 3 combined concepts in PubMed [15]. Concept 1 was designed to identify published studies that used RWE or RWD in the absence of specific Medical Subject Heading (MeSH) terms in PubMed. Concept 2 introduced the 3 target countries. Concept 3 included a combination of terms to identify studies that fitted the eligibility criteria for this scoping review (Multimedia Appendix 1, page 8). The search terms used were as follows:

- Concept 1: “Treatment Outcome” (MeSH) OR “Evidence-Based Medicine” (MeSH) OR “Retrospective Studies” (MeSH) OR “Time Factors” (MeSH) OR “real world” OR “real-world” OR “RWD” OR “RWE” OR “real life” OR “real patient” OR “real practice” OR “real clinical” OR “real population” OR “actual world” OR “actual life” OR “actual patient” OR “actual practice” OR “actual clinical” OR “actual population”
- Concept 2: “India” (MeSH) OR “Taiwan” (MeSH) OR “Thailand” (MeSH) OR “India” OR “Taiwan” OR “Thailand”
- Concept 3: “Electronic Health Records” (MeSH) OR “Insurance, Health” (MeSH) OR “Registries” (MeSH) OR “Databases, Pharmaceutical” (MeSH) OR “Pharmaceutical Services” (MeSH) OR “registry” OR “registries” OR “electronic health record*” OR “electronic health care record*” OR “electronic medical record*” OR “EHR” OR “EHRs” OR “EMR” OR “EMRs” OR “claims database*” OR “administrative database*” OR “hospital data” OR “claims data” OR “electronic health data” OR “clinical database*” OR “electronic health care data” OR “informatics”

In addition to these search terms, we applied 2 filters: English language and publication within 5 years before the date of the literature search. We chose these filters to help identify the RWE or RWD of interest to an international community and also identify databases that are in current (or recent) use.

Manual searching of the references cited in the eligible studies or reviews was not performed. We used a single database (PubMed) to limit the extent of duplicates and to focus on English language studies published in indexed, peer-reviewed journals.

The abstracts of all studies retrieved by the literature search were downloaded into Covidence software (Veritas Health Innovation Ltd) for screening and data extraction.

### Study Eligibility and Screening

The next phase involved screening of the retrieved studies against eligibility criteria (Multimedia Appendix 1, page 8) [15]. These eligibility criteria were chosen to help us identify relevant studies reporting RWE and RWD. The eligibility criteria covered 4 aspects: database type, publication type, study type, and publication scope.

Our intent was to retrieve original research papers using electronic medical records (EMRs)/electronic health records (EHRs), health insurance/administrative claims, clinical registries, or pharmacy databases, provided the data were collected across multiple hospitals/clinics. Brief reports, short communications, and research letters were eligible if they reported original data. Randomized controlled trials, pragmatic controlled trials, preclinical studies, and nonhuman studies were excluded. Studies that involved nontarget countries were eligible, provided they also included data from one or more target countries.

After removing duplicates in an automated process, the studies were screened for eligibility in a 2-phase process in Covidence. Phase 1 involved screening the titles and abstracts. For studies that passed phase 1, their full text was obtained for phase 2. Due to the number of studies retrieved by the PubMed search, phases 1 and 2 were conducted by 3 reviewers; in addition, comprehensive quality control was not feasible, so a fourth reviewer spot-checked a random selection of 20% of the studies. Discussions were held among the reviewers about ambiguous studies or any contradictions or discrepancies between reviewers until a consensus was reached. If there was no consensus, another reviewer was consulted. Data extraction was carried out in phase 3.

### Data Extraction

Data were extracted into Covidence using a custom template, which included the following variables:

- Covidence identification number, study identifier, and title and year of publication of each eligible study. The study identifier was standardized as the last name of the first author and the year of publication.
- Publication type (clinical study or protocol).
- Study type as comparative effectiveness research (CER) or descriptive research (non-CER). The definition of CER was standardized during the data extraction phase adapted from MeSH [18], which defines CER as “studies primarily comparing interventions and strategies (including the comparison between active and non-active interventions/strategies) to prevent, diagnose, treat, and monitor health conditions using validated methods for confounders elimination, e.g., matching, and statistical adjustments like stratification, weighting, regression, instrumental variable analysis etc.”
- Database type based on the source of data. This included medical records (EMRs or EHRs), health insurance/administrative claims, clinical registries, pharmacy claims, or mixed databases involving more than one type.
- Disease name/condition studied and disease area. The categorization of disease areas for the studies was defined based on the primary diagnosis, in line with the pathophysiology, and, if overlapping, based on the in-charge medical specialty.
- Study outcomes separated as clinical (benefit or safety), cost, or patient-reported outcomes (PROs). PRO studies are defined as studies that incorporate measures of health outcomes that are directly reported by patients themselves [19].
- Study population (adults, pediatric, or mixed).
- Study duration, number of study centers, and study sample size. The study duration (research period) was measured in years and was calculated as the time in years from the study’s start date to end date or the end of a follow-up period, as explicitly stated by the authors. The lag from the end of the research period to publication was also calculated in years.
- Database name used by the author (if specified in the publication) grouped by database type.

Data were extracted by 6 reviewers in parallel, and due to the size of the database, without duplicate or overlapping data entries, the ability to conduct thorough quality control was limited. However, a spot check was carried out for the first 50 extractions, where discrepancies in interpreting a few variables were identified and corrected. This process also involved standardizing some definitions, re-extracting data from the initial batch, and additional regular spot checks. Regular discussions were held among reviewers to ensure consistent and accurate data extraction. Again, any contradictions or discrepancies were discussed until a consensus was reached. If there was no consensus, another reviewer was consulted.

### Data Analysis

Data extracted into Covidence were exported into a Microsoft Excel spreadsheet for descriptive analyses (Multimedia Appendix 2). The included studies were then categorized as single-country studies (SCS) and cross-county collaboration studies (CCCS) based on whether they used databases from one of the target countries or multiple countries.

The included studies were grouped by clinical research topics into 5 disease areas: cardiology and metabolic disorders (CVM); oncology; inflammatory and autoimmune disorders (IAD); infectious diseases and vaccines (IDV); and others. The “others” category was a residual category designed to capture studies outside the 4 main areas, including neurological, psychiatric/mental health, respiratory, gastrointestinal, musculoskeletal, renal and urological, and dermatological disorders.

To adjust the partial year data for 2017 and 2022 (the PubMed final search was conducted on September 27, 2022, with a filter for 2017-2022), we calculated the annual study numbers for these years by multiplying the mean quarterly study number by 4. We used linear regression, with the year as a continuous explanatory variable, to calculate the linear trend in the number of studies per year. This approach allowed us to understand the average change in the number of studies for each year over the study period. A 2-year simple moving average (SMA) was also calculated for the percentage of total studies to smooth out random fluctuations from one year to the next. SMA involves taking the arithmetic mean of a set of values over a specific period, which, in our case, was 2 consecutive years [20]. The purpose of using SMA is to improve systematic error detection by smoothing out the fluctuations in data points [21]. As this was a descriptive study, we did not perform statistical hypothesis tests. We presented categorical data as frequencies and percentages and continuous data as mean (SD) values.

All data analyses for this research were conducted in Excel. For the creation of high-resolution images in the main manuscript, we used Adobe Illustrator. We confirm that no generative artificial intelligence tools were used, either as stand-alone systems or in conjunction with PubMed or Covidence, during the ideation, search strategy, screening, data extraction, data analysis, or manuscript-writing phase of this research.

## Results

### General Characteristics of Studies

**Figure 1:**
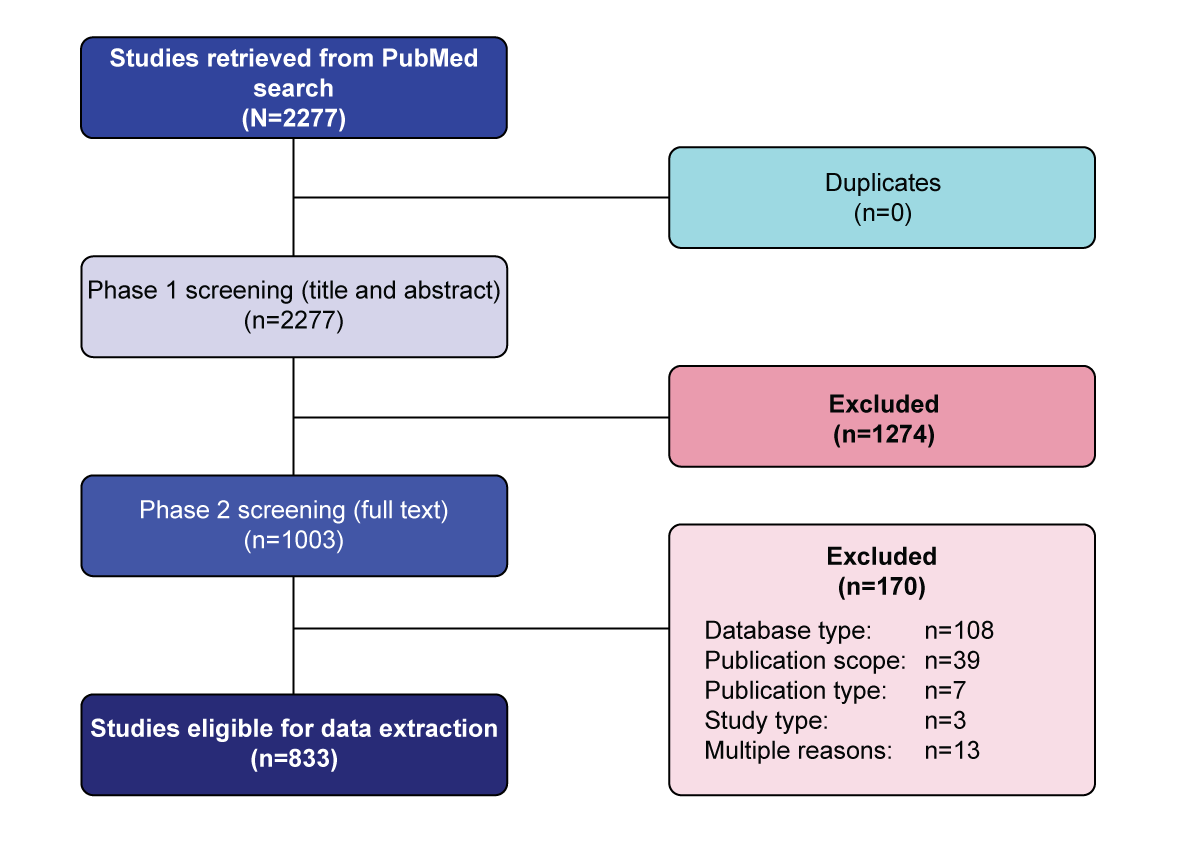
Literature search for eligible studies. Reasons for exclusion: database type (the study used a database other than EHRs/EMRs, health insurance/administrative claims, clinical registries, or pharmacy claims or data from only 1 hospital), publication scope (the study did not include India, Taiwan, or Thailand), publication type (correspondence, editorial/commentary, guideline, case report/series, review), study type (randomized controlled trial, pragmatic controlled trial, preclinical study, or nonhuman study), and other reasons (country, design, data type, number of centers, or number of patients not clearly stated). EHR: electronic health record; EMR: electronic medical record; PRISMA: Preferred Reporting Items for Systematic Reviews and Meta-Analyses.

As previously described, a PubMed literature search was conducted on September 27, 2022. The search yielded a total of 2277 studies with no duplicates. Of these, 1003 (44%) were included in phase 2 screening, and 833 (36.6%) studies were eligible for data extraction (Figure 1). The general descriptive characteristics of all eligible studies, SCS, and CCCS are presented, respectively, in Tables S1-S3 in Multimedia Appendix 3.

### Regional Distribution and Time Trend

Among the eligible studies, 88 (10.6%) were CCCS and 745 (89.4%) were SCS; 623 (83.6%) SCS were from Taiwan, 81 (10.9%) from India, and 41 (5.5%) from Thailand. Of the total studies in each country, there was a higher proportion of CCCS in India (52/133, 39.1%) and Thailand (31/72, 43.1%) than in Taiwan (51/674, 7.6%). Table 1 shows the study distribution in the 3 countries and their collaborative relationship. Despite the variations in the number of SCS across the 3 target countries, their participation in CCCS was fairly similar, with Taiwan having 51 (38.1%) studies, India having 52 (38.8%), and Thailand having 31 (23.1%); see Table 2. The breakdown and characteristics of the 88 CCCS are summarized in Multimedia Appendix 4 (page 2) and Table S3 in Multimedia Appendix 3, respectively.

**Table 1:** Overview of eligible studies from linked databases in single target countries and collaborative studies between target or nontarget countries.

Country	SCS^a^ from individual target countries (n=745), n (%)	CCCS^b^ between target/nontarget countries, including duplicates (n=134^c^), n (%)					Total CCCS by country, n (%)	Total studies (N=879^d^) by country, n (%)
		Other countries (nontarget; n=52)	Nontarget countries and Taiwan (n=17)	Nontarget countries and India (n=16)	Nontarget countries and Thailand (n=19)	All countries (Taiwan, India, Thailand; n=30)		
Taiwan	623 (83.6)	24 (46.2)	N/A^e^	7 (43.8)	10 (52.6)	10 (33.3)	51 (7.6)	674 (76.7)
India	81 (10.9)	26 (50.0)	7 (41.2)	N/A	9 (47.4)	10 (33.3)	52 (39.1)	133 (15.1)
Thailand	41 (5.5)	2 (3.8)	10 (58.8)	9 (56.2)	N/A	10 (33.3)	31 (43.1)	72 (8.2)

^a^SCS: single-country studies.

^b^CCCS: cross-country collaboration studies.

^c^Duplications in CCCS adjusted; the number of CCCS after removing duplicates reduced from 134 to 88.

^d^Duplications in total studies adjusted; the number of studies after removing duplicates reduced from 879 to 833.

^e^N/A: not applicable.

**Table 2:** Breakdown of eligible SCS^a^ and overall CCCS^b^ by study characteristics (N=879)^c^.

Study characteristics			Taiwan (n=674)				India (n=133)					Thailand (n=72)		
			SCS (n=623)		CCCS (n=51)		SCS (n=81)		CCCS (n=52)		SCS (n=41)			CCCS (n=31)
**Study type, n (%)**														
	CER^d^	410 (65.8)		14 (27.5)		12 (14.8)		10 (19.2)		11 (26.8)			9 (29.0)	
	Non-CER (descriptive)	213 (34.2)		37 (72.5)		69 (85.2)		42 (80.8)		30 (73.2)			22 (71.0)	
**Database type, n (%)**														
	EMR^e^/EHR^f^	71 (11.4)		13 (25.5)		45 (55.6)		9 (17.3)		9 (22.0)			5 (16.1)	
	Clinical registry	224 (36.0)		39 (76.5)		38 (46.9)		44 (84.6)		31 (75.6)			26 (83.9)	
	Health insurance/administrative claim	458 (73.5)		5 (9.8)		1 (1.2)		0		4 (9.8)			0	
	Pharmacy claim	2 (0.3)		0		0		0		0			0	
	Multiple databases	128 (20.5)		5 (9.8)		3 (3.7)		1 (1.9)		3 (7.3)			0	
**Disease area, n (%)**														
	CVM^g^	139 (22.3)		19 (37.3)		35 (43.2)		25 (48.1)		9 (22.0)			15 (48.4)	
	Oncology	150 (24.1)		8 (15.7)		8 (9.9)		5 (9.6)		12 (29.3)			5 (16.1)	
	IAD^h^	62 (10.0)		2 (3.9)		6 (7.4)		3 (5.8)		2 (4.9)			1 (3.2)	
	IDV^i^	32 (5.1)		3 (5.9)		9 (11.1)		4 (7.7)		2 (4.9)			3 (9.7)	
	Others	240 (38.5)		19 (37.3)		23 (28.4)		15 (28.8)		16 (39.0)			7 (22.6)	
**Study outcomes, n (%)**														
	Clinical	609 (97.8)		50 (98.0)		81 (100)		51 (98.1)		41 (100)			31 (100)	
	Cost	34 (5.5)		0		0		1 (1.9)		0			0	
	PROs^j^	3 (0.5)		3 (5.9)		0		3 (5.8)		0			1 (3.2)	
**Study population, n (%)**														
	Adults	514 (82.5)		38 (74.5)		47 (58.0)		34 (65.4)		29 (70.7)			23 (74.2)	
	Mixed	91 (14.6)		13 (25.5)		29 (35.8)		12 (23.1)		11 (26.8)			7 (22.6)	
	Pediatric	18 (2.9)		0		5 (6.2)		6 (11.5)		1 (2.4)			1 (3.2)	
Study duration (years), mean (SD)			10.2 (5.4)		6.2 (5.1)		5.4 (5.5)		7.6 (10.2)		7.5 (6.8)			7.0 (7.9)
**Lag period (years) from end of research to publication**														
	Overall mean (SD)	7.1 (2.6)		4.1 (1.9)		3.7 (1.7)		4.9 (2.6)		4.8 (1.9)			4.3 (2.3)	
	<2, n (%)	20 (3.2)		9 (17.6)		21 (25.9)		9 (17.3)		4 (9.8)			6 (19.4)	
	2-5, n (%)	128 (20.5)		25 (49.0)		44 (54.3)		21 (40.4)		22 (53.7)			19 (61.3)	
	≥6, n (%)	467 (75.0)		10 (19.6)		11 (13.6)		11 (21.2)		13 (31.7)			3 (9.7)	
	Unknown, n (%)	7 (1.1)		8 (15.7)		5 (6.2)		11 (21.2)		2 (4.9)			3 (9.7)	
**Study size, mean (SD)**														
	Sample size (in thousands)	175.6 (1087.3)		3390.3 (22,279.2)		143.4 (479.6)		140.2 (426.5)		57.5 (220.6)			28.0 (44.7)	
	Number of centers	21.5 (44.0)		342.3 (715.1)		63.9 (155.4)		399.0 (814.3)		20.2 (16.9)			223.1 (298.5)	

^a^SCS: single-country studies.

^b^CCCS: cross-country collaboration studies.

^c^Study numbers for database types and study outcomes may appear as duplicates; hence, the total percentage may not account for 100%. There were 46 duplicates due to >1 target country participating in the same CCCS; the total number of studies after removing duplicates was 833. The percentages may add up to less or more than 100 because of rounding.

^d^CER: comparative effectiveness research.

^e^EHR: electronic health record.

^f^EMR: electronic medical record.

^g^CVM: cardiology and metabolic disorders.

^h^IAD: inflammatory and autoimmune disorders.

^i^IDV: infectious diseases and vaccines.

^j^PRO: patient-reported outcome.

**Figure 2:**
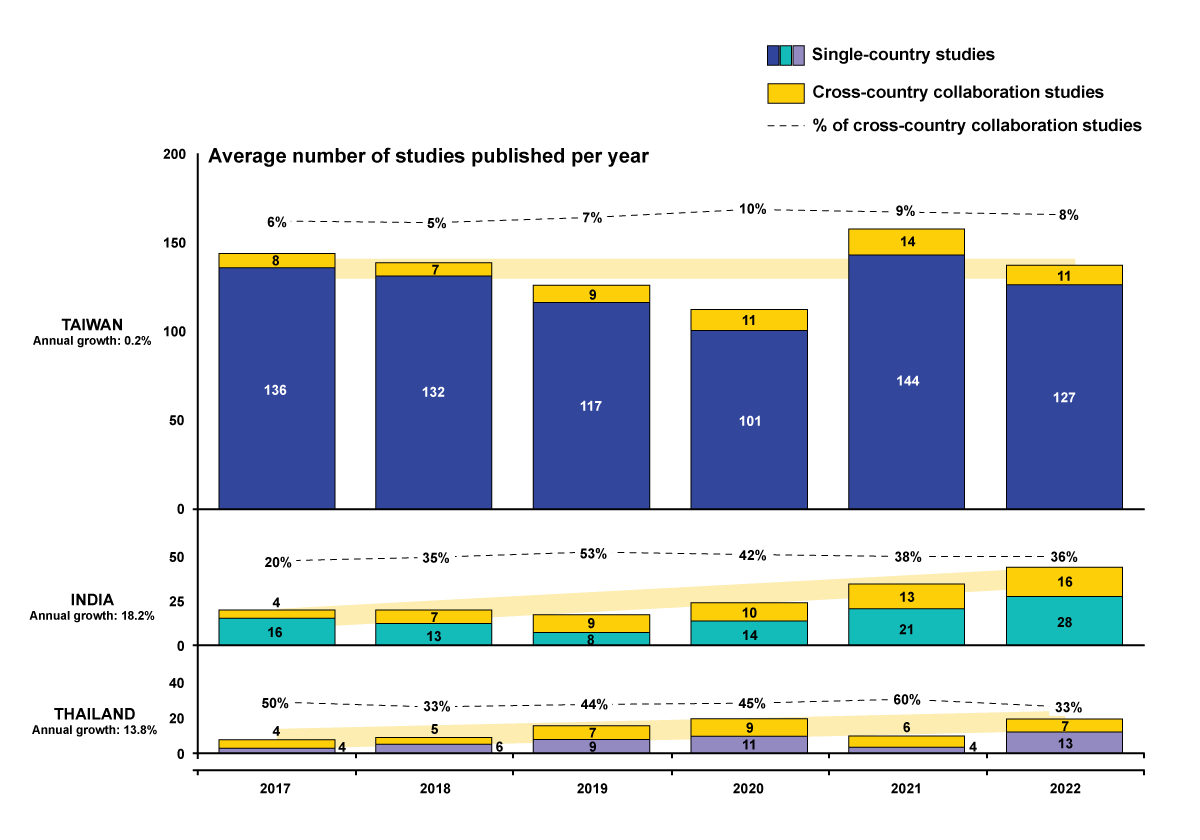
Annual trend and geography distribution for SCS and CCCS (N=833). India had the highest growth in the total studies among the 3 target countries. Taiwan had the most stable number of studies, while Thailand showed moderate growth and the highest percentage of cross-country collaborations. Although Taiwan has maintained a steady level of international collaboration studies, both India and Thailand are increasing their involvement in cross-country collaborative research. CCCS: cross-country collaboration studies; SCS: single-country studies.

The average number and growth rates of SCS and CCCS per year in the 3 target countries are illustrated in Figure 2. From 2017 to 2022, the number of studies published from India and Thailand showed an increasing trend, with an annual growth rate of 18.2% and 13.8%, respectively, but remained stable for Taiwan. The number of CCCS mirrored the total study count in each target country, with India displaying a stronger upward trend and Thailand showing a more inconsistent upward trend with a wider variation across the years.

Table 2 explains the key characteristics of the included studies, further broken down for SCS and CCCS by study type, disease area, database type, study outcomes, study population, duration, lag period from end of the research to publication, study sample size, and study center. Nearly all the publications (n=831, 99.8%) were clinical studies; the remaining 2 (0.2%) were study protocols (Table S1 in Multimedia Appendix 3).

### Study Characteristics

#### Study Type: CER or Non-CER (Descriptive)

**Figure 3:**
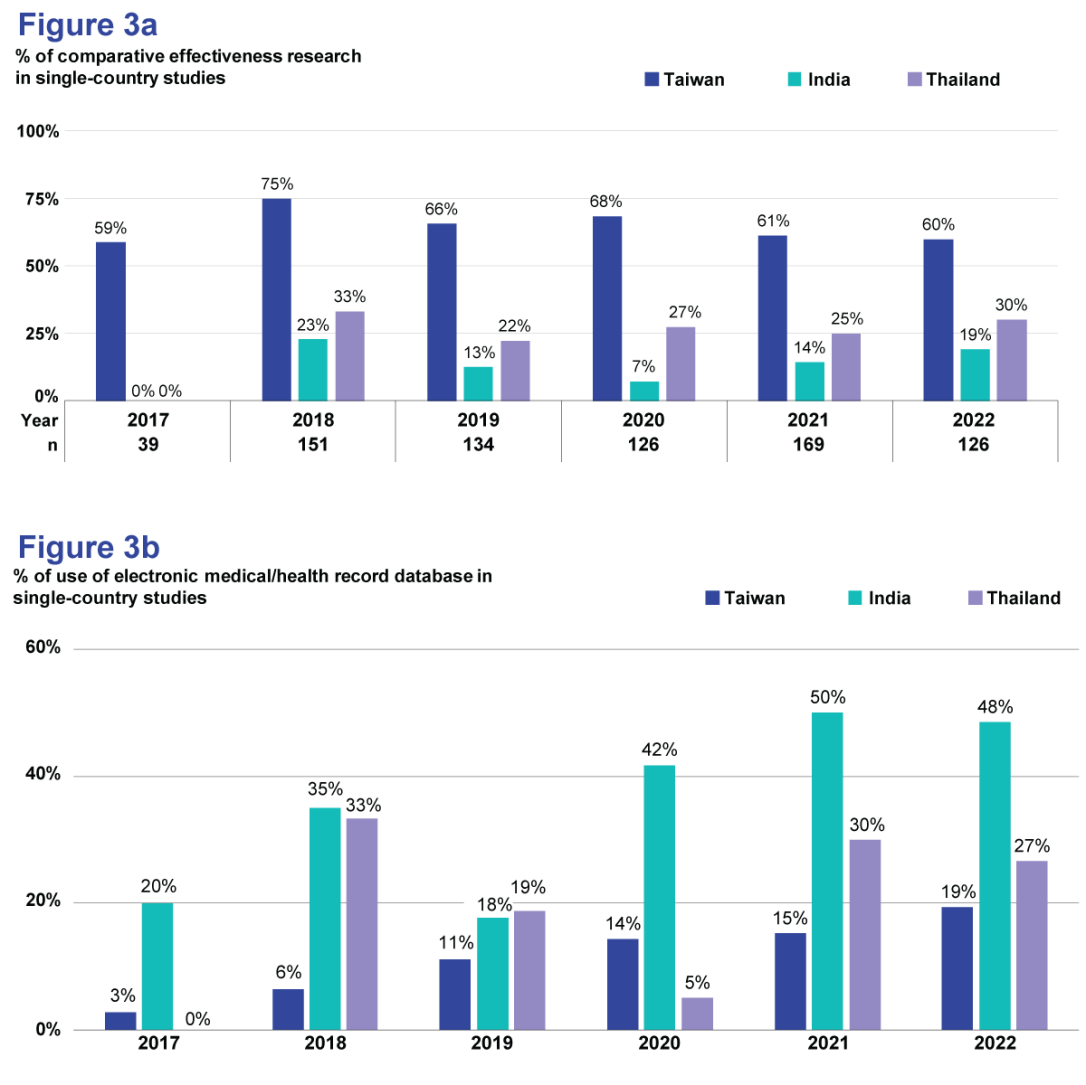
Distribution and time trend in SCS (n=745) for (a) percentage of CER and (b) percentage of EMR/EHR database use (either independently or in conjunction with other databases). CER: comparative effectiveness research; EHR: electronic health record; EMR: electronic medical record; SCS: single-country studies.

Overall, of the 833 studies retrieved, there were 454 (54.5%) CER-based studies, while the remaining 379 (45.5%) were non-CER-based (descriptive) studies (Table S1 in Multimedia Appendix 3). In addition, 410 (65.8%) were CER-based SCS from Taiwan. However, this type of research accounted for only 14.8% (n=12) of SCS from India, 26.8% (n=11) of SCS from Thailand, and 24.6% (n=33) of all combined CCCS (Table 2 and Multimedia Appendix 4, page 3). The differences in CER percentages between Taiwan and other countries remained consistent throughout 2017-2022 (Figure 3a). However, when data were averaged over 2 consecutive years, the proportion of CER was found to steadily decrease over time in SCS from Taiwan but increased in India and remained broadly constant in Thailand (Multimedia Appendix 4, page 4).

#### Database Type

Among the 833 studies retrieved, a single database type was used in 693 (83.2%) studies, with health insurance/administrative claims data being the most common (n=349, 41.9%), followed by clinical registries (n=237, 28.5%) and EMRs/EHRs (n=107, 12.8%); no study used a pharmacy claims database as the sole data source. Meanwhile, 140 (16.8%) studies used multiple types of databases, particularly a combination of a clinical registry with health insurance/administrative claims (n=101, 12.1%); see Table S1 in Multimedia Appendix 3. The adoption of multiple databases was far more common in SCS from Taiwan (n=127, 20.4%) than in India (n=3, 3.7%) and Thailand (n=3, 7.3%). A similar trend was observed for CCCS (Tables S2 and S3 in Multimedia Appendix 3). In general, the majority of SCS from Taiwan (n=458, 73.5%) used health insurance/administrative claims databases, either independently or in conjunction with other databases. In contrast, in India, a larger proportion of SCS used EMRs/EHRs (n=45, 55.6%) and clinical registries (n=38, 46.9%), while SCS from Thailand mainly used clinical registries (n=31, 75.6%), and the use of EMRs/EHRs accounted for only 22% (n=9) of SCS (Table 2). Despite a low overall usage of EMRs/EHRs in Taiwan (n=71, 11.4%), there was a notable increase in their usage from 1/36 (2.8%) to 20/103 (19.4%) over a span of 2017-2022 (Figure 3b). Overall, the proportion of studies that exclusively used EMRs/EHRs as the only database in the 3 target countries steadily increased from 8/165 (4.8%) to 57/231 (24.7%) in 2017-2022. Conversely, the use of health insurance/administrative claims databases decreased from 111/165 (67.3%) to 101/231 (43.7%); see Multimedia Appendix 4 (page 5). In relation to CCCS, no discernible trend over time was detected (Multimedia Appendix 4, page 6).

#### Disease Area

**Figure 4:**
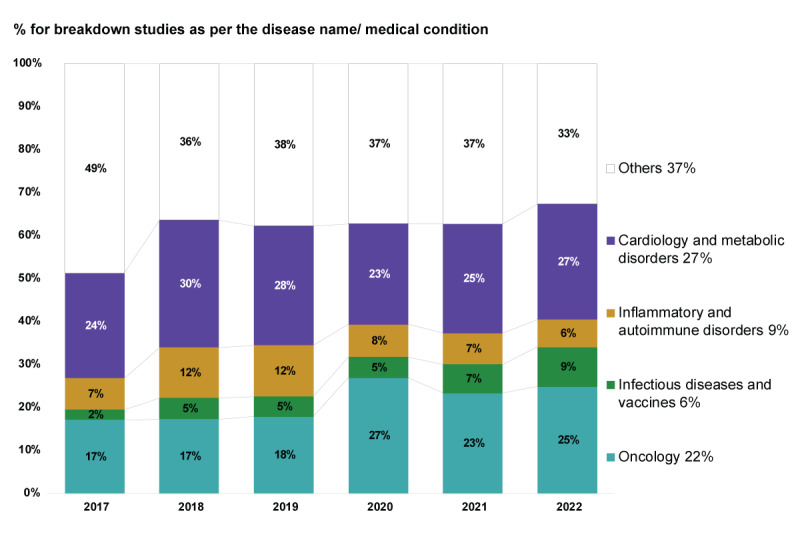
Distribution and time trend of the percentage of studies conducted in different medical areas (N=833). Studies in the CVM medical area made up 29.1% (242/833) of the total, with a slight fluctuation year to year. Research focusing on the oncology field increased from 17% to 25% from 2017 to 2022, while that focusing on the IDV field grew from 2% to 9% during the same period. CVM: cardiology and metabolic disorders; IDV: infectious diseases and vaccines.

In the 833 studies retrieved, the 2 most common medical areas of research were CVM (n=242, 29.1%) and oncology (n=188, 22.6%), followed by IAD (n=76, 9.1%) and IDV (n=53, 6.4%). The remaining 320 (38.4%) studies covered other diseases (Table S1 in Multimedia Appendix 3). The proportion of the “other” category reduced from 20/41 (48.8%) in 2017 to 46/141 (32.6%) in 2022. Oncology studies increased from 7/41 (17.1%) to 35/141 (24.8%), and IDV studies increased from 1/41 (2.4%) to 13/141 (9.2%), while CVM and IAD studies remained steady (Figure 4). CVM and oncology were the 2 most common disease areas of focus in SCS from Taiwan and Thailand (n=139, 22.3%, and n=12, 29.3%, respectively), whereas India had a high proportion of studies on CVM (n=35, 43.2%), with only 8 (9.9%) studies on oncology (Table 2). CVM was the most studied area in CCCS and accounted for 37.3% (n=19) of research in Taiwan and was similar in both India and Thailand (n=25, 48.1%, and n=15, 48.4%, respectively); see Table 2.

#### Study Outcome(s) Analyzed

Nearly all the 833 studies (n=792, 95.1%) reported clinical outcomes (in terms of a benefit or safety), and a further 22 (2.6%) reported clinical outcomes together with cost outcomes (Table S1 in Multimedia Appendix 3). Only a small proportion of studies reported cost or PRO outcomes alone. All SCS from Thailand and India and 609 (97.8%) SCS from Taiwan reported clinical outcomes. Another 34 (5.5%) SCS from Taiwan and 1 (1.9%) CCCS from India reported study outcomes related to cost, while 3 (5.8%) CCCS from India and 3 (5.9%) CCCS from Taiwan reported PROs (Multimedia Appendix 4, pages 7 and 8).

#### Study Population

Among the 833 studies retrieved, the majority (n=651, 78.2%) involved adults, 31 (3.7%) involved the pediatric population, and 151 (18.1%) involved a combination of adult and pediatric populations (Table S1 in Multimedia Appendix 3). Intriguingly, among SCS, the distribution of population varied among the 3 countries (Table 2). In Taiwan, 514 (82.5%) studies involved adults, 18 (2.9%) involved the pediatric population, and 91 (14.6%) involved mixed populations. The respective values were 47 (58%), 5 (6.2%), and 19 (35.8%) in India and 29 (70.7%), 1 (2.4%), and 11 (26.8%) in Thailand. The pediatric population was more represented in CCCS from India compared to SCS from India, at 11.5% (n=6) and 6.2% (n=5), respectively (Table 2).

#### Study Duration

Among the 833 studies retrieved, information about study duration (or duration of data collection/follow-up) was reported for 802 (96.3%) studies. The median duration was 9.0 years, with an average of 9.4 (SD 6.0) years, and ranged from 0.1 to 65.8 years. In total, 21 (2.6%) studies had a duration of >20 years (Multimedia Appendix 4, page 9).

The mean duration for SCS was 10.2 (SD 5.4) years in Taiwan, 5.4 (SD 5.5) years in India, and 7.5 (SD 6.8) years in Thailand (Table 2). The mean study duration in Taiwan remained broadly stable (~10 years) over 2017-2022, with no particular trend observed for CCCS. However, the mean study duration for CCCS was relatively shorter in Taiwan and relatively longer in India (Multimedia Appendix 4, pages 10 and 11).

#### Lag Between the Research Period and Publication

Of the 833 eligible studies, the majority (n=510, 61.2%) were published ≥6 years after the research period, 234 (28.1%) were published 2-5 years after the research period, and 58 (7%) were published within 2 years of the research period (Multimedia Appendix 4, page 12). However, the publication patterns of India, Thailand, and Taiwan differed. Specifically, most SCS from India and Thailand were published 2-5 years after the research period. In contrast, the majority of SCS from Taiwan were published ≥6 years after the research period (Table 2). It is noteworthy that there was an upward trend in the publication of studies within 2 years of the research period for both SCS and CCCS (Multimedia Appendix 4, pages 13 and 14).

The publication time lag also varied by the source of data. Among studies that used an exclusive single database, the majority of EMR/EHR database and clinical registry studies were published 2-5 years after the research period, while health insurance/administrative claims studies were mostly published ≥6 years after the research period. A higher proportion of studies from EMR/EHR databases were published within 2 years of the research period compared to clinical registries and health insurance/administrative claims databases for both SCS and CCCS. Specifically, for SCS, 19/90 (21.1%) EMR/EHR database studies were published within 2 years of the research period compared to 22/162 (13.6%) clinical registry studies and no health insurance/administrative claims studies. Similarly, for CCCS, 8/19 (42.1%) EMR/EHR database studies were published within 2 years of the research period compared to 16/89 (18%) clinical registry studies and no health insurance/administrative claims studies (Multimedia Appendix 4, pages 15 and 16).

#### Study Size: Sample Size and Number of Centers

Among the 833 studies retrieved, the sample size was specified in 813 (97.6%) studies and varied considerably, ranging from as few as 20 to over 154,500,000, averaging at 352,814 with a median of 9831 (Multimedia Appendix 4, page 17). SCS from Taiwan tended to be larger (mean 175,600, SD 1,087,300) than in India (mean 143,400, SD 479,600) and Thailand (mean 57,500, SD 220,600); see Table 2. This pattern was consistent over time based on 2-year smoothed averages (Multimedia Appendix 4, page 18). CCCS from Taiwan had the largest mean sample size (3,390,300, SD 22,279,200), while the mean sample size of CCCS from Thailand was smaller (28,000, SD 44,700). The mean sample size for CCCS from India (140,200, SD 426,500) was roughly similar to that for SCS (Table 2).

In addition, the number of participating centers was reported in 214 (25.6%) studies. The median number of centers was 13, ranging from 2 to 2746, and the mean was 105.5 (SD 378.2); see Multimedia Appendix 4 (page 19). The mean number of centers reported in SCS from Taiwan, India, and Thailand was 21.5 (SD 44.0), 63.9 (SD 155.4), and 20.2 (SD 16.9), respectively (Table 2). One SCS from Taiwan [22] warrants mention here; the study involved patients admitted to a single intensive care unit (ICU), and the authors used the health insurance database to collate additional information. Although we excluded SCS, this study was included due to the use of data from institutions other than the original ICU. The numbers were considerably higher for CCCS, with averages of 342,300 (SD 715,100) for Taiwan, 399,000 (SD 814,300) for India, and 223,100 (SD 298,500) for Thailand (Table 2). There was a trend of an increase in the mean number of study centers in SCS from India in the past 3 years (Multimedia Appendix 4, page 20). No particular time trend was noticed for study centers in CCCS across the target countries (Multimedia Appendix 4, page 21).

### Database Names

Where specified, the name of the database used in each study was extracted and tabulated by target country and database type as per the disease area. The names of the databases are listed in Multimedia Appendix 5 (pages 2-39 for Taiwan, 40-43 for India, and 44-47 for Thailand).

Databases in Taiwan included primarily health insurance/administrative claims related to the National Health Insurance Research Database (NHIRD) and its subsets (Longitudinal Health Insurance Database [LHID] 2000, 2005, and 2010 or the Longitudinal Cohort of Diabetes Patients) and linked databases. Noteworthy registries, mostly linked to the NHIRD, were the Taiwan Cancer Registry Database (TCRD), the Registry for Catastrophic Illness Patients (RFCIP), and the Taiwan Death Registry (TDR), as well as more disease-specific registries, such as the Taiwan Stroke Registry, the Taiwan Renal Registry Data System (TWRDS), the Taiwan Hepatitis C Virus (HCV) registry, the Taipei Out-of-Hospital Cardiac Arrest (OHCA) registry, the Taiwan Bone Marrow Transplant Registry, and the Acute Coronary Syndrome–Diabetes Mellitus (ACS-DM) registry of the Taiwan Society of Cardiology (TSOC). Regarding EMRs/EHRs, the most common source was the Chang Gung Memorial Hospital (CGMH) and its affiliated hospitals/clinics.

In India, the main databases were clinical registries of cardiology and metabolic disorders, such as the Envision en-ABL-e registry, the Nanoluté Registry, and the Primary Percutaneous Coronary Intervention (PPCI) registry of Kerala. Others included oncology registries, such as the OncoCollect Lymphoma Registry, the Association of Surgical Gastroenterologists of Kerala (ASGK) colorectal cancer (CRC) registry, and others, such as Towards Improved Trauma Care Outcomes in India (TITCO) and the Indian Pediatric Continuous Renal Replacement Therapy–International Collaboration of Nephrologists

and Intensivists for Critical Care Children (PCRRT-ICONIC) Neonatal Kidney Educational Registry (TINKER). Registries were followed by large EMR/EHR databases, including HealthPlix EMR and EyeSmart EMR, whereas the notable health insurance/administrative claims database was linked to the Arogyasri health insurance scheme.

In Thailand, clinical registries were mostly related to CVM (eg, the COOL-AF registry, the Thai Type 1 Diabetes and Diabetes Diagnosed Before Age 30 Years Registry, Care and Network [T1DDAR CN], the Cardiac Intervention Association of Thailand [CIAT]), oncology (eg, the Khon Kaen cancer registry, the Thai Lymphoma Study Group [TLSG] registry), and IAD (eg, the Thai Rheumatic Disease Prior Authorization [RDPA] registry). Health insurance/administrative claims databases were used in oncology (the Nationwide Hospital Admission Data Registry) and other disease areas, which included the 43-files database, the Universal Coverage Health Security Insurance Scheme Database Thailand, and the Universal Coverage Scheme (UCS) claim data set under the National Health Security Office (NHSO). EMRs/EHRs were combined with the Thai Type 1 Diabetes and Diabetes Diagnosed Age Before 30 years Registry, Care and Network (T1DDAR CN); the Khon Kaen Cancer Registry, Khon Kaen Central Hospital; and the Region 7 Office of Disease Prevention and Control.

## Discussion

### Principal Findings

This scoping review was performed with the objectives of identifying and characterizing the databases used for RWE generation in Asian countries, with the expectation that the information gained can be used to support the development of RWE in Asian health care strategy and policy. As a pilot, we focused on 3 Asian countries with diverse economic and health care settings: India, Taiwan, and Thailand.

We identified studies reporting RWE/RWD in these 3 countries by searching PubMed using a 3-concept search strategy combined with filters for the English language and publication within 5 years of the search date. A total of 833 studies were retrieved by the search and were eligible for data extraction/analysis, of which 745 used databases from 1 of the 3 target countries alone (SCS), primarily Taiwan (83.6%), followed by India (10.9%) and Thailand (5.5%). From 2017 to 2022, India and Thailand exhibited a surge in published RWD studies, growing annually at around 18.2% and 13.8%, respectively, while Taiwan’s output remained stable. The count of CCCS corresponded with each country’s total studies, with India showing a robust upward trend, whereas Thailand displayed a more uneven ascent. The increasing trend in the number of studies published from India and Thailand could be attributed to increased investments in research by local and international bodies and improvement in research infrastructure.

RWE is derived from a mixture of CER and non-CER (descriptive) research, where the former is helpful for gauging the impact of a novel/alternative treatment or preventive/prognostic strategy on patient outcomes and the latter may provide insight into the current clinical situation (eg, the prevalence of a disease or pattern of a treatment usage). We observed a shift in the type of research over time. This shift was particularly evident in Taiwan, where the proportion of studies reporting CER decreased from 72% in 2017-2018 to 61% in 2021-2022. By comparison, CER increased over this period in India (from 29% to 33%). The specific health challenges faced by each country generally shape its research focus, along with changes in policy directions. For example, the rising burden of noncommunicable diseases in India might have necessitated more CER to inform treatment choices [23,24]. Similarly, Taiwan’s vision for 2030 emphasizes “Precision Health” as 1 of its 6 core strategic industries. This approach leverages a wealth of physiological and genomic data, alongside external factors, to devise individualized preventive measures and treatment strategies [25]. These efforts align closely with population health management strategies in Taiwan, which necessitate a comprehensive understanding of disease prevalence and treatment patterns. Although the specific reasons behind these trends remain uncertain, it is possible that the COVID-19 pandemic may have influenced these shifts. These trends do, however, signify the evolving need for RWE in guiding health care decisions and research priorities and informing policy making within the countries.

We found marked differences in the types of databases used in the studies performed in the 3 countries. Notably, the health insurance/administrative claims database was used as a data source in 73.5% of SCS from Taiwan versus 1.2% from India and 9.8% from Thailand (with 55% of the studies using it as an exclusive data source in Taiwan). Instead, a larger proportion of SCS from India used EMRs/EHRs (55.6%, with 52% of the studies using them as an exclusive data source) and clinical registries (46.9%, with 43% of the studies using them as an exclusive data source). In contrast, SCS from Thailand mainly used clinical registries (75.6%, with 68% of the studies using them as an exclusive data source). The use of EMRs/EHRs accounted for 22% of SCS from Thailand, with 15% of the studies using it as an exclusive data source. These characteristics largely reflect the clinical settings of each country. Taiwan provides national health insurance with UHC through a single scheme that facilitates the development of a single, centralized database (NHIRD), which is linked to over 70 additional clinical databases [26]. By comparison, India largely relies on a self-pay health care system, and Thailand offers a health insurance scheme with UHC and differential decentralized benefit packages. The current health care system in India is decentralized and does not have extensive, centralized databases [27,28], but it is rapidly enhancing access to local EMR/EHR systems with the “Digital Health Mission,” which also aids in relevant RWD generation [29]. The predominant use of clinical registries for RWD generation in Thailand probably also reflects a strong culture of clinical registries, which might be due to specific health policies or a historical practice [30]. We found that a large proportion of the studies were in the fields of CVM and oncology. This may reflect the frequencies of these diseases, as well as their social and financial burdens, creating a driver for research in these disease areas to provide RWE and support health care decision-making.

We also assessed other aspects of the studies, including study duration (period of data collection), number of participating centers, and sample sizes, where reported. These characteristics varied considerably, reflecting the heterogeneity in the analyzed studies as well as the study populations. We found that the majority of studies from integrated databases were published ≥6 years after the research period, but studies from India and Thailand had a shorter time to publication compared to those from Taiwan. The specific reasons for these publication patterns are unclear but might be related to many factors, including how quickly data are made available or uploaded into the database, the data analysis time, and the time to write a manuscript and submit it to a journal. However, the higher proportion of CER and the greater use of the health insurance/administrative claims database in Taiwan might have also influenced these results. Although RWD studies are generally appreciated for their potential to quickly generate insights, it is important to consider that several factors related to data cleaning, consolidation, harmonization, and analysis may require considerable time, and ensuring robust quality and validity of data can be a complex and time-consuming task. We also observed a rising trend in the number of studies being published within 2 years of the research period. Over time, an increasing proportion of studies were published quicker, which may indicate more efficient research practices or a growing emphasis on timely dissemination of findings.

Although each database may have specific strengths, weaknesses, or data structures that make it more or less suitable for a particular analysis or research question, overall, we found that the adoption and use of pharmacy claims database were low in all the 3 pilot countries in Asia. In addition, data from EMR/EHR databases tended to be published more quickly after the research period compared to clinical registry data, and studies using health insurance/administrative claims databases took the longest time for publication compared to EMR/EHR and clinical registry databases. The lag time for clinical registry studies may be reasonable if we consider that many clinical registries are carried out at a fixed point in time, or over a set period, with a long interval between the research period and data collection via forms completed by research staff. The data from clinical registries may also be available for researchers for many years afterward. The prominence of the use of clinical registries in >80% of CCCS in our study underscores their crucial role in international RWE generation.

It is more difficult to explain the long lag time for studies using health insurance/administrative claims databases. As mentioned before, there is a significant lag before patient data are uploaded to health insurance/administrative claims databases, estimated to be about 2 years for the Taiwan NHIRD [26]. We should also consider that some studies may involve a long follow-up or data collection period that is not necessarily reflected in our analysis. Regardless of the underlying factors, our findings suggest that studies using health insurance/administrative claims databases do not capture the most recent information from clinical practice. Thus, such studies may not reveal the current clinical practice but rather practices from several years earlier.

The unique and common names for identified databases in our research will facilitate communication and collaboration among stakeholders working with RWD sources. This will enhance the ability of stakeholders to share knowledge and insights across borders and to leverage the strengths and resources of different countries, enabling the generation of robust RWD. To provide more insight for readers and assist in their future considerations of available RWD, a deeper analysis of various other databases and their use in Asia is essential. We recognize this need and plan to undertake further research in this area in other Asian countries.

We are aware of a few studies with a similar objective or design as our scoping review [31,32]. The first of these [32], published in 2019, involved a literature search of Medline and Embase for the period from January 1, 2010, to September 8, 2015, and retrieved 10,069 publications, which used a total of 2635 unique data sources from 102 countries. Consistent with our findings, the authors of that study found that administrative databases (1656 unique data sources) were the most common unique source of information. More recently, Rogers et al [31] reported a literature search of 3 databases (PubMed, Embase, and CINAHL) for the period from January 1, 2009, to February 7, 2020, to identify clinical trials (or interventional studies) using RWD that were performed in the United States [31]. Although neither of these 2 studies [31,32] can be directly compared with our own due to the different objectives and search strategies involved, the findings highlight the expansive range of RWD and its increasing significance to inform health care decisions and policy.

### Limitations

Through a carefully designed search strategy, this scoping review captured a large number of eligible studies reporting RWE in 3 target countries. As a direct consequence, there were limits on the amount of data that we could extract from the individual studies due to practical limitations and the varied level of details presented in the studies. Therefore, the data presented here represent just the tip of the huge amount of data that could, conceivably, be extracted from these studies with sufficient resources. Although we focused on presenting the narrative for key identified databases in this paper, a future in-depth analysis of databases of countries and cross-border studies will provide a more comprehensive overview of the research landscape and help identify potential areas for collaboration and knowledge sharing among stakeholders.

We should also acknowledge the fact that many studies did not report certain characteristics (eg, number of centers, study period) or that they covered multiple settings or designs, making it difficult to extract data with consistent accuracy. For example, some eligible studies reported the actual study duration, but others reported the enrollment period with or without the end of the follow-up/observation period. Study screening and data extraction were performed by multiple reviewers due to time constraints, without duplicate or overlapping data entry. Quality control involved spot checks and discussions among reviewers, considering the available resources and time. Although this approach enabled us to manage the workload and streamline the review process, it is important to acknowledge that it may have led to some unintended inaccuracies in our findings. As such, we recommend interpreting the findings of this review with an understanding of the potential limitations associated with using a single reviewer.

Furthermore, it is possible that a few relevant studies may have been incorrectly excluded when screening the titles/abstracts if, for example, the abstracts omitted any mention of RWD/RWE sources. Despite these limitations, the literature review retrieved a large number of studies that met our eligibility criteria, and we have provided comprehensive insight into the use of RWD for generating RWE in the chosen countries.

A final limitation worth noting is the decision to focus on English language publications and exclusively using PubMed for our citation search strategy. We made this decision to identify studies that would be of interest to an international community and considering that the bulk of medical literature is in English. Similarly, we chose to limit our search to PubMed as part of our strategy to identify robust RWD from fully published, indexed, peer-reviewed medical journals, while also minimizing duplicate results. Expanding the search to non-English language journals and additional literature databases may have retrieved a few additional relevant studies, which would have helped improve the number of eligible studies. Overall, however, we are confident that these limitations would not have had a substantial effect on our findings.

While acknowledging the limitations of this study, it is also an opportunity to advocate for more transparency and standardization in reporting the study design, methods, number of study centers, and study duration in RWD studies. One specific area that demands more clarity and standardization is the reporting of the study duration, which encompasses both the patient recruitment phase and the patient follow-up or observation period.

### Conclusion

This systematic scoping review identified some clear differences in the types of RWD used in Taiwan, India, and Thailand, as well as time-dependent trends, that at least partly reflect the divergent economic and clinical settings and the availability of research data sources in these countries. The data extracted here provide a simple snapshot of the 3 countries, and further insights may be obtained in future analyses or with further data extraction. The methods used here could be upscaled and transferred to other countries or regions, allowing researchers to obtain tailored insights. The research will foster within-country and cross-border collaboration between researchers, health care institutions, drug manufacturers, and policy makers by allowing access to more diverse and comprehensive databases, ultimately improving the quality and relevance of research and decision-making in health care.

## Appendix

Multimedia Appendix 1 Graphical overview of introduction and methodology.

Multimedia Appendix 2 Databases for eligible studies.

Multimedia Appendix 3 Supplementary tables.

Multimedia Appendix 4 Supplementary figures.

Multimedia Appendix 5 Identified databases.

## Abbreviations

CCCS: cross-country collaboration studies
CER: comparative effectiveness research
CVM: cardiology and metabolic disorders
EHR: electronic health record
EMR: electronic medical record
HTA: health technology assessment
IAD: inflammatory and autoimmune disorders
ICU: intensive care unit
IDV: infectious diseases and vaccines
MeSH: Medical Subject Headings
NHIRD: National Health Insurance Research Database
PRO: patient-reported outcome
RWD: real-world data
RWE: real-world evidence
SCS: single-country studies
SMA: simple moving average
UHC: universal health coverage
