# Supplementary material for: Integrated Real-World Study Databases in 3 Diverse Asian Health Care Systems in Taiwan, India, and Thailand: Scoping Review
Source: J Med Internet Res. 2023 Sep 11;25:e49593. doi: 10.2196/49593 (PMC10520767; doi:10.2196/49593)
Supplement: Multimedia Appendix 1 [file jmir_v25i1e49593_app1.pdf]

Multimedia Appendix 1

Scoping review to identify and describe integrated contemporary real-world studies databases from three diverse healthcare systems in Asia: Taiwan, India, and Thailand

Wen-Yi Shau, Sajita Setia, Ying-Jan Chen, Tsu-yun Ho, Salil Shinde, Handoko Santoso, Daniel Furtner

International Registered Report Identifier (IRRID): RR2-10.2196/43741

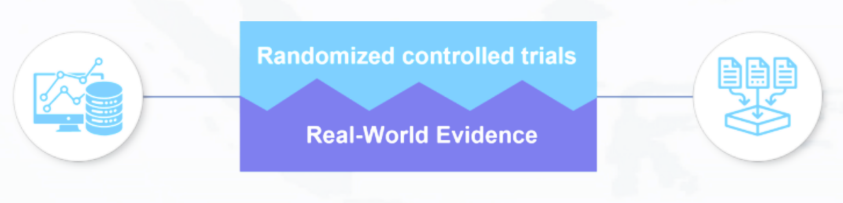

INTRODUCTION

Contemporary databases for real-world studies of three diverse healthcare systems in Asia

Scoping review for India, Thailand, and Taiwan

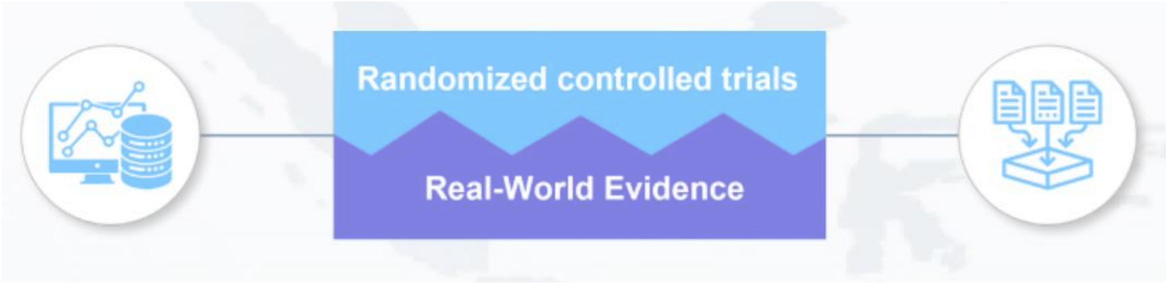

Purpose of this research

The collection of real-world evidence (RWE) is increasingly crucial in Asia

Only ~17% of clinical trials are conducted in Asia, and Asian populations are often under-represented in pivotal clinical trials.[1,2]

RWE provides certainty about the safety and effectiveness of medications, health interventions and technologies in the local setting for Asian patients.[1]

Unmet needs remain in increasing the adoption of RWE in Asia to support regulatory and clinical decision-making.[3]

Generating fit-for-purpose real-world evidence in Asia

To support the benefits of RWE in Asian healthcare strategy, planning and policy, we identified **linked contemporary databases** used in **real-world studies** in **three representative countries in Asia** by performing a **systematic scoping review.**[4]

Abbreviations: RCT, randomized controlled trial; RWD, real-world data; RWE, real-world evidence

References: 1. Lou J, et al. Int J Technol Assess Health Care. 2020 Oct;36(5):474-80. PMID: 32928330. 2. https://hiper.nus.edu.sg/wp-content/uploads/2021/03/REALISE-Full-guidance-post-feedback_20201211-version-1.1.pdf. Last accessed: Feb 9, 2023. 3. McNair D et al. Clin Pharmacol Ther. 2022 Jan;111(1):44-51. PMID: 34655224. doi: 10.1002/cpt.2449. 4. Shau WY et al. JMIR Res Protoc. 2022 Dec 13;11(12):e43741.

Rationale for selecting three diverse countries: India, Thailand, and Taiwan

*Asia is a very diverse region. We chose a representative country for three economies in Asia.**

	**Diverse economies in Asia**	**Diverse health-care reimbursement in Asia**
**Taiwan**	High-income economy	Health insurance for *universal coverage* and a single social health insurance scheme exists
**Thailand**	Upper-middle-income economy	Health insurance for universal coverage with *differential de-centralised* benefit packages
**India**	Lower-middle income economy	Largely a *self-pay* healthcare system

*These economies were defined according to the World Bank analysis for the 2023 fiscal year, whereby low-income economies are those with a gross national income (GNI) per capita, calculated using the World Bank Atlas method, of $1,085 or less in 2021; lower-middle-income economies are those with a GNI per capita between $1,086 and $4,255; upper-middle-income economies are those with a GNI per capita between $4,256 and $13,205; high-income economies are those with a GNI per capita of $13,205 or more (World Bank country and lending groups. The World Bank Group. URL: https://datahelpdesk.worldbank.org/knowledgebase/articles/906519-world-bank-country-and-lending-groups)

.

References: **1.** Shau WY, Setia S, Shinde SP, Santoso H, Furtner D. Contemporary Databases in Real-world Studies Regarding the Diverse Health Care Systems of India, Thailand, and Taiwan: Protocol for a Scoping Review. JMIR Res Protoc. 2022 Dec 13;11(12):e43741.

How will this research add value to RWD generation in Asia?

Databases identified in this review will serve as a basis to further guide approaches and initiatives to drive collaboration/ improvements in generation & utilisation of RWE in healthcare decision-making in Asia.

Research findings are crucial for nurturing advancement in RWD generation in Asia by:

Understanding dynamics, trends and variations for RWD research in representative countries for three diverse economies and health-care reimbursement systems in Asia

Our identified linked RWD databases may open the path to new avenues for public-private partnerships

Multiple collaborations to expand the scope and spectrum of high-quality, robust RWE generation in Asia.

Abbreviations: RWD, real-world data; RWE, real-world evidence

METHODOLOGY

Contemporary databases for real-world studies of three diverse healthcare systems in Asia

Scoping review for India, Thailand, and Taiwan

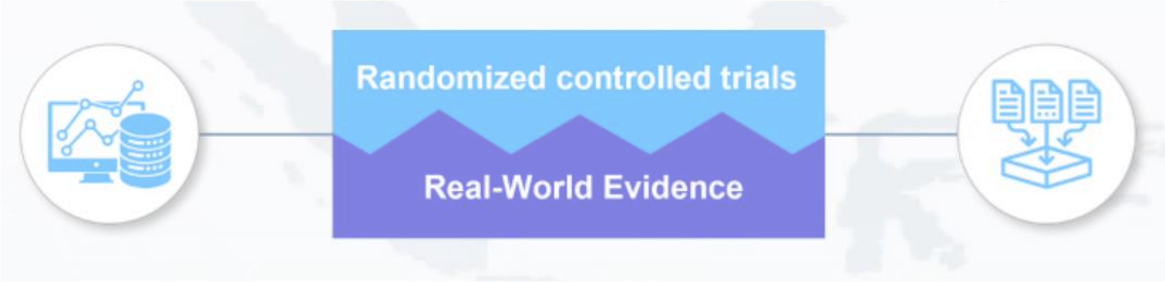

Methodology

The study protocol and methodology adhered to the PRISMA Extension for Scoping Reviews (PRISMA-ScR) guidelines.

01 Scoping Data

The PubMed search strategy covered three concepts

- Concept 1 was designed to identify potential RWE/RWD studies by applying a variety of MeSH terms (“Treatment Outcome”, “Evidence-Based Medicine”, “Retrospective Studies”, “Time Factors”) and related keywords (e.g. “real-world”, “actual life”, “actual practice”).
- Concept 2 introduced the three countries (India, Taiwan, and Thailand).
- Concept 3 focused on data types, by using a combination of MeSH terms (“Electronic Health Records”, “Insurance, Health”, “Registries”, “Databases, Pharmaceutical”, “Pharmaceutical Services”) and related keywords (e.g., electronic medical record, electronic healthcare record, EMR, EHR, administrative database, registry).

These searches were combined with filters for the English language and publications in the last 5 years before the search

02 Screening

- The retrieved articles underwent Phase 1 (title and abstract) and Phase 2 (full text) screening phases

03 Data Extraction

- Data extraction of eligible citations are done using **Covidence software**.

Abbreviations: RCT, randomized controlled trial; RWD, real-world data; RWE, real-world evidence

References: 1. Shau WY, Setia S, Shinde SP, Santoso H, Furtner D. Contemporary Databases in Real-world Studies Regarding the Diverse Health Care Systems of India, Thailand, and Taiwan: Protocol for a Scoping Review. JMIR Res Protoc. 2022 Dec 13;11(12):e43741.

Inclusion and exclusion criteria for eligible citations retrieved from PubMed

Inclusion criteria	Exclusion criteria
**Database types**	
• Studies involving electronic health records, health insurance/administrative claims, clinical registries, or pharmacy databases<br>**AND**<br>• Databases with research data involving >1 hospital/clinic were included	• A data source involving electronic health records, health insurance/administrative claims, clinical registries, or pharmacy databases not mentioned were excluded<br>**OR**<br>• Databases with research data involving 1 hospital/clinic were excluded
**Publication types**	
The following publication types were included:<br>Original research, including brief reports/short communications/research letters	The following publication types were excluded:<br>• Correspondence/letters to the editor<br>• Case reports, case series*<br>• Narrative, systematic, or scoping reviews<br>• Editorials/commentary<br>• Guidelines
**Study types**	
All types of real-world studies or their protocols using the following databases were included:<br>• Electronic health records<br>• Health insurance/administrative claims<br>• Clinical registries<br>• Pharmacy databases	The following types of studies were excluded:<br>• RCTs<br>• PCTs<br>• Pre-clinical studies<br>• Non-human studies
**Scope of publication**	
Studies with databases involving Taiwan, India, or Thailand. Eligible international/regional/multi-country studies were included, provided any of the target countries were included	Studies with a scope outside Taiwan, India, or Thailand were excluded

***Publications with prospective descriptions of a handful of patient cases; retrospective case series with a real-world data study design[2] were eligible for inclusion (Glen S. "Retrospective Study: Case-Control and Case-Series" From StatisticsHowTo.com: Elementary Statistics for the rest of us! Available from: https://www.statisticshowto.com/retrospective-study/)

Abbreviations: PCTs, pragmatic clinical trials; RCTs, randomised controlled trials

References: 1. Shau WY, Setia S, Shinde SP, Santoso H, Furtner D. Contemporary Databases in Real-world Studies Regarding the Diverse Health Care Systems of India, Thailand, and Taiwan: Protocol for a Scoping Review. JMIR Res Protoc. 2022 Dec 13;11(12):e43741.
