# Supplementary material for: Integrated Real-World Study Databases in 3 Diverse Asian Health Care Systems in Taiwan, India, and Thailand: Scoping Review
Source: J Med Internet Res. 2023 Sep 11;25:e49593. doi: 10.2196/49593 (PMC10520767; doi:10.2196/49593)
Supplement: Multimedia Appendix 3 [file jmir_v25i1e49593_app3.pdf]

Appendix 3: Supplemental tables

Supplemental Table 1. Descriptive characteristics of the included studies (N = 833)

**Characteristic**	***N* (%)**
Total	833 (100)
Publication type	
Clinical study	831 (100)
Protocol	2 (0)
Country/cluster	
Taiwan	623 (75)
India	81 (10)
Thailand	41 (5)
Taiwan; One/more non-target countries	26 (3)
India; One/more non-target countries	24 (3)
Thailand; One/more non-target countries	10 (1)
Taiwan; Thailand; One/more non-target countries	10 (1)
India; Taiwan; One/more non-target countries	9 (1)
India; Thailand; One/more non-target countries	7 (1)
India; Taiwan; Thailand; One/more non-target countries	2 (0)
Publication year	
2017 [a]	41 (5)
2018	162 (19)
2019	151 (18)
2020	145 (17)
2021	193 (23)
2022 [a]	141 (17)
Disease areas	
Cardiology and metabolic disorders	221 (27)
Oncology	181 (22)
Inflammatory and autoimmune disorders	74 (9)
Infectious diseases and vaccines	49 (6)
Others	308 (37)
Study population	
Adults	651 (78)

Paediatric	31 (4)
Mixed	151 (18)
Database type/source	
Health insurance/claims	349 (42)
Clinical registry	237 (29)
EMR/EHR	107 (13)
Clinical registry; Health insurance/claims	101 (12)
EMR/EHR; Clinical registry	20 (2)
EMR/EHR; Health insurance/claims	12 (1)
EMR/EHR; Clinical registry; Health insurance/claims	5 (1)
EMR/EHR; Pharmacy claims	1 (0)
Health insurance/claims; Pharmacy claims	1 (0)
Study type	
CER	454 (55)
Descriptive (non-CER)	379 (45)
Main study outcome	
Clinical (benefit/safety)	792 (95)
Clinical (benefit/safety); Cost	22 (3)
Clinical (benefit/safety); PROs	4 (0)
Cost	13 (2)
PROs	2 (0)

[a]The search was conducted on September 27, 2023, with a filter for studies published within 5 years of the search date.

CER, comparative effectiveness research; EHR, electronic health records; EMR, electronic medical records; PRO, patient-reported outcome

Supplemental Table 2. Descriptive characteristics of the included single-country studies using database(s) from target countries; (N = 745)

	Taiwan (74.7%)	India (9.7%)	Thailand (4.9%)
Total	623 (100)	81 (100)	41 (100)
Publication type			
Clinical study	623 (100)	80 (99)	42 (100)
Protocol	0	1 (1)	0
Publication year			
2017[a]	34 (5)	4 (5)	1 (2)
2018	132 (21)	13 (16)	6 (15)
2019	117 (19)	8 (10)	9 (22)
2020	101 (16)	14 (17)	11 (27)
2021	144 (23)	21 (26)	4 (10)
2022[a]	95 (15)	21 (26)	10 (24)
Disease area			
Cardiology and metabolic disorders	139 (22)	35 (43)	9 (22)
Infectious diseases and vaccines	32 (5)	9 (11)	2 (5)
Inflammatory and autoimmune disorders	62 (10)	6 (7)	2 (5)
Oncology	150 (24)	8 (10)	12 (29)
Others	240 (39)	23 (28)	16 (39)
Study population			
Adults	514 (83)	47 (58)	29 (71)
Paediatric	18 (3)	5 (6)	1 (2)
Mixed	91 (15)	29 (36)	11 (27)
Database type/source			
Clinical registry	108 (17)	35 (43)	28 (68)
Clinical registry; Health insurance/claims	100 (16)	0	0
EMR/EHR	44 (7)	42 (52)	6 (15)
EMR/EHR; Clinical registry	12 (2)	3 (4)	3 (7)
EMR/EHR; Clinical registry; Health insurance/claims	4 (1)	0	0
EMR/EHR; Health insurance/claims	10 (2)	0	0
EMR/EHR; Pharmacy claims	1 (0)	0	0
Health insurance/claims	343 (55)	1 (1)	4 (10)
Health insurance/claims; Pharmacy claims	1 (0)	0	0
Study type			

Comparative effectiveness study	410 (66)	12 (15)	11 (27)
Descriptive (non-CER)	213 (34)	69 (85)	30 (73)
Subcategories based on study outcome			
Clinical (benefit/safety)	586 (94)	81 (100)	41 (100)
Clinical (benefit/safety); Cost	21 (3)	0	0
Clinical (benefit/safety); PROs	2 (0)	0	0
Cost	13 (2)	0	0
PROs	1 (0)	0	0

Values are *n* (%) of studies

[a]The search was conducted on September 27, 2023, with a filter for studies published in English and within 5 years of the search date.

CER, comparative effectiveness research; EHR, electronic health records; EMR, electronic medical records; PRO, patient-reported outcome

Supplemental Table 3. Descriptive characteristics of the included collaboration studies using database(s) from >1 countries (*N* = 88)

	Taiwan and one/more non-target countries	India and one/more non-target countries	Thailand and one/more non-target countries	Taiwan, Thailand and one/more non-target countries	India, Taiwan and one/more non-target countries	India, Thailand and one/more non-target countries	India, Taiwan, Thailand and one/more non-target countries
Total	24 (100)	26 (100)	2 (100)	10 (100)	7 (100)	9 (100)	10 (100)
Publication type							
Clinical study	24 (100)	25 (96)	2 (100)	10 (100)	7 (100)	9 (100)	10 (100)
Protocol	0	1 (4)	0	0	0	0	0
Publication year							
2017[a]	1 (4)	2 (8)	0	0	0	1 (11)	1 (10)
2018	3 (13)	5 (19)	1 (50)	0	3 (43)	2 (22)	1 (10)
2019	5 (21)	5 (19)	1 (50)	2 (20)	0	3 (33)	2 (20)
2020	5 (21)	9 (35)	0	4 (40)	0	1 (11)	2 (20)
2021	8 (33)	5 (19)	0	3 (30)	1 (14)	2 (22)	2 (20)
2022[a]	2 (8)	2 (8)	0	1 (10)	3 (43)	1 (11)	2 (20)
Disease area							
Cardiology and metabolic disorders	6 (25)	14 (54)	1 (50)	6 (60)	3 (43)	4 (44)	4 (40)
Infectious diseases and vaccines	1 (4)	1 (4)	1 (50)	0	1 (14)	1 (11)	1 (10)
Inflammatory and autoimmune disorders	1 (4)	1 (4)	0	0	1 (14)	1 (11)	2 (20)
Oncology	4 (17)	2 (8)	0	2 (20)	0	1 (11)	3 (30)
Others	12 (50)	8 (31)	0	2 (20)	2 (29)	2 (22)	4 (40)

Study population							
Adults	17 (71)	16 (62)	1 (50)	9 (90)	5 (71)	6 (67)	7 (70)
Paediatric	0	6 (23)	0	0	0	0	0
Mixed	7 (29)	4 (15)	1 (50)	1 (10)	2 (29)	3 (33)	3 (30)
Database type/source							
Clinical registry	14 (58)	21 (81)	1 (50)	8 (80)	5 (71)	8 (89)	9 (90)
Clinical registry; Health insurance/claims	1 (4)	0	0	0	0	0	0
EMR/EHR	4 (17)	4 (15)	1 (50)	2 (20)	2 (29)	1 (11)	1 (10)
EMR/EHR; Clinical registry	1 (4)	1 (4)	0	0	0	0	0
EMR/EHR; Clinical registry; Health insurance/claims	1 (4)	0	0	0	0	0	0
EMR/EHR; Health insurance/claims	2 (8)	0	0	0	0	0	0
Health insurance/claims	1 (4)	0	0	0	0	0	0
Study type							
CER	6 (25)	5 (19)	0	5 (50)	1 (14)	2 (22)	2 (20)
Descriptive (non-CER)	18 (75)	21 (81)	2 (100)	5 (50)	6 (86)	7 (78)	8 (80)
Subcategories based on study outcomes							
Clinical (benefit/safety)	24 (100)	25 (96)	2 (100)	10 (100)	5 (71)	9 (100)	9 (90)
Clinical (benefit/safety); Cost	0	1 (4)	0	0	0	0	0
Clinical (benefit/safety); PROs	0	0	0	0	1 (14)	0	1 (10)
PROs	0	0	0	0	1 (14)	0	0

Values are *n* (%) of studies

[a]The search was conducted on September 27, 2023, with a filter for studies published in English and within 5 years of the search date.

CER, comparative effectiveness research; EHR, electronic health records; EMR, electronic medical records; PRO, patient-reported outcome
