# Supplementary material for: Integrated Real-World Study Databases in 3 Diverse Asian Health Care Systems in Taiwan, India, and Thailand: Scoping Review
Source: J Med Internet Res. 2023 Sep 11;25:e49593. doi: 10.2196/49593 (PMC10520767; doi:10.2196/49593)
Supplement: Multimedia Appendix 4 [file jmir_v25i1e49593_app4.pdf]

Multimedia Appendix 4 – supplemental figures

Scoping review to identify and describe integrated contemporary real-world studies databases from three diverse healthcare systems in Asia: Taiwan, India, and Thailand

Wen-Yi Shau, Sajita Setia, Ying-Jan Chen, Tsu-yun Ho, Salil Shinde, Handoko Santoso, Daniel Furtner

International Registered Report Identifier (IRRID): RR2-10.2196/43741

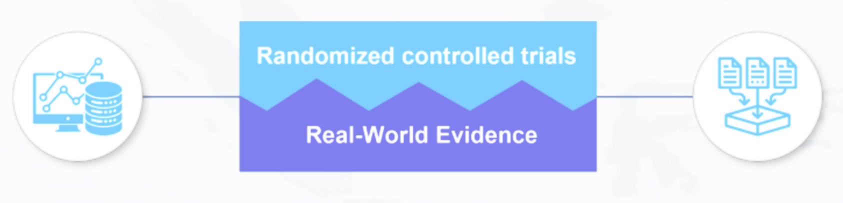

Country/cluster wise RWD studies from integrated databases

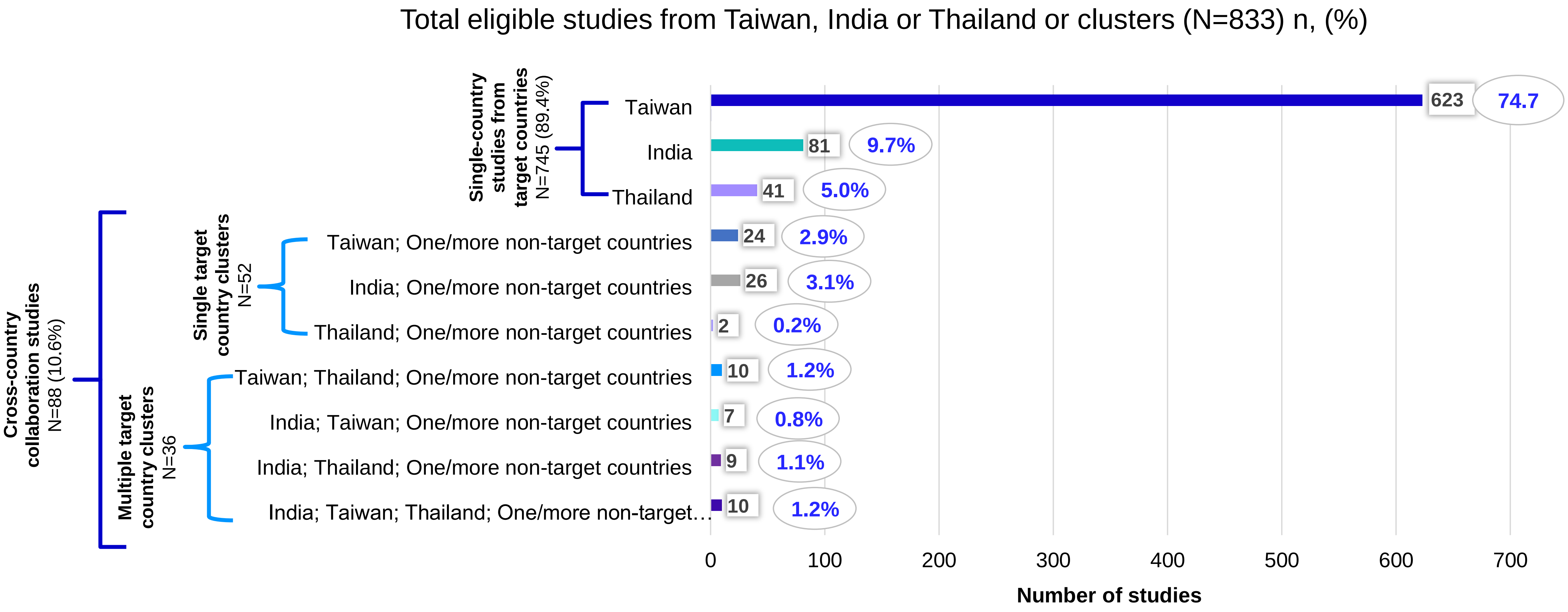

Abbreviations: RWD, real-world data

Study types from integrated databases for all included studies

Study types of all eligible studies from all 3 target countries/clusters (N=833) (n, %)

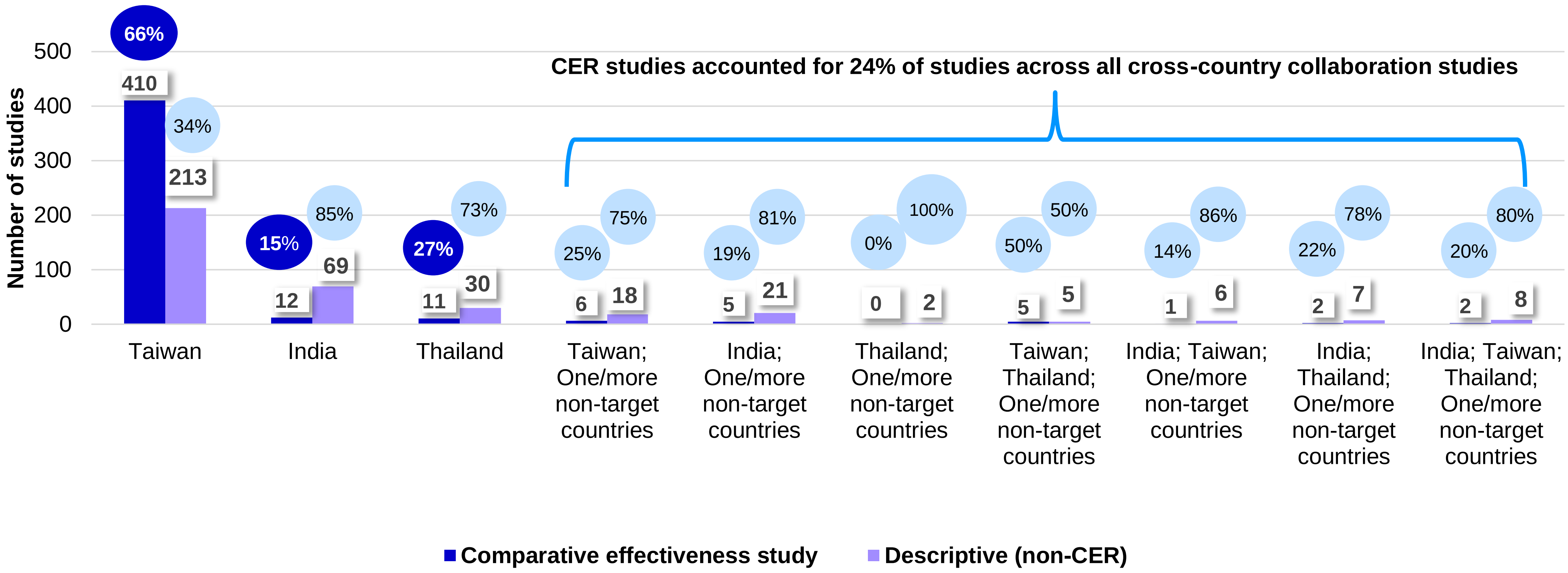

Taiwan is the only country contributing more CER than descriptive research

Abbreviations: CER, comparative effectiveness research; RWD, real-world data

Time trend for CER for single country and collaborative studies

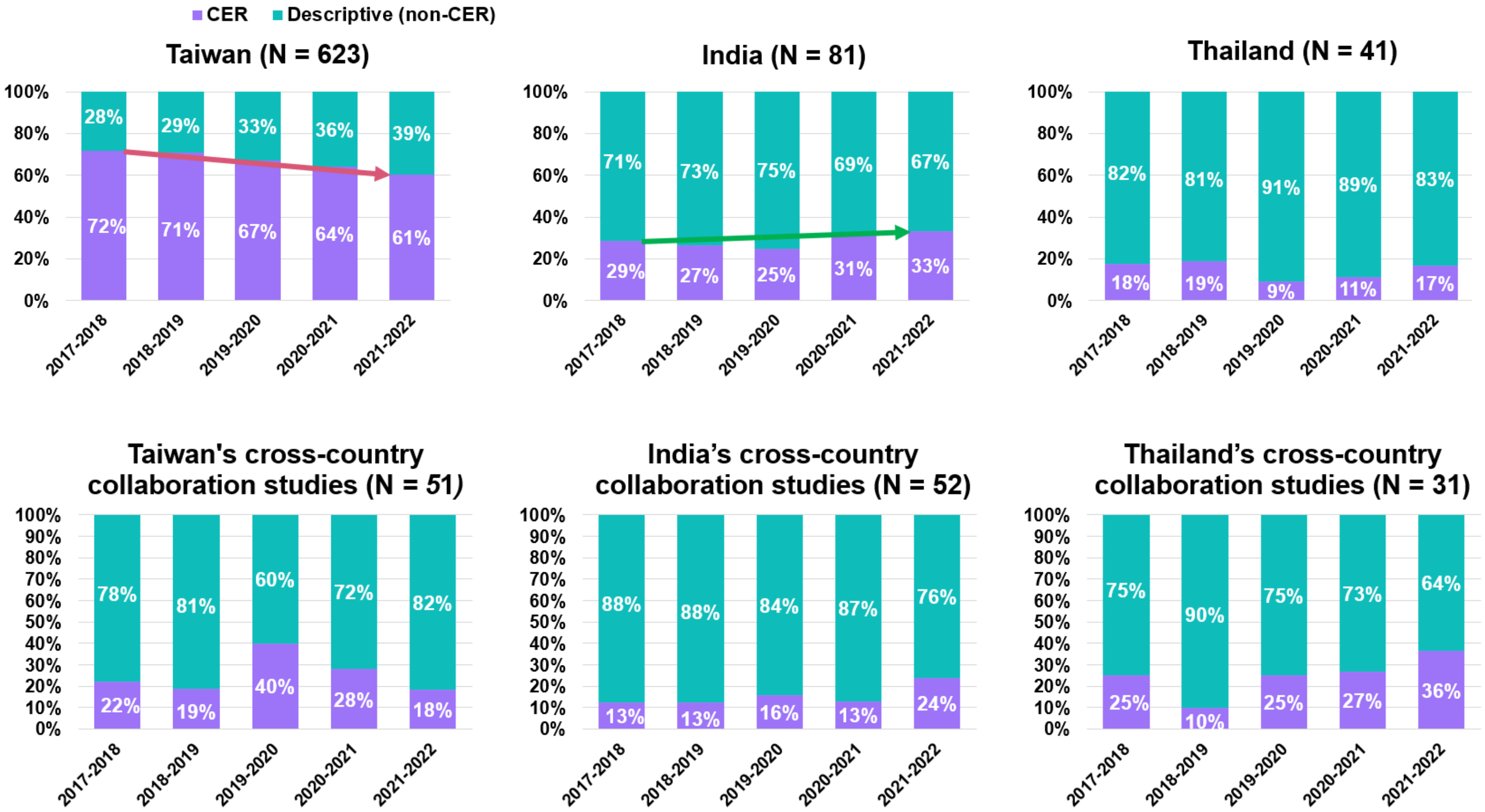

Study numbers from cross-country collaborative studies may appear as duplicates for studies conducted in multiple target countries.

Abbreviations: CER, comparative effectiveness research; RWD, real-world data

Percentage of single exclusive database utilisation for RWD studies from single-country studies of target countries

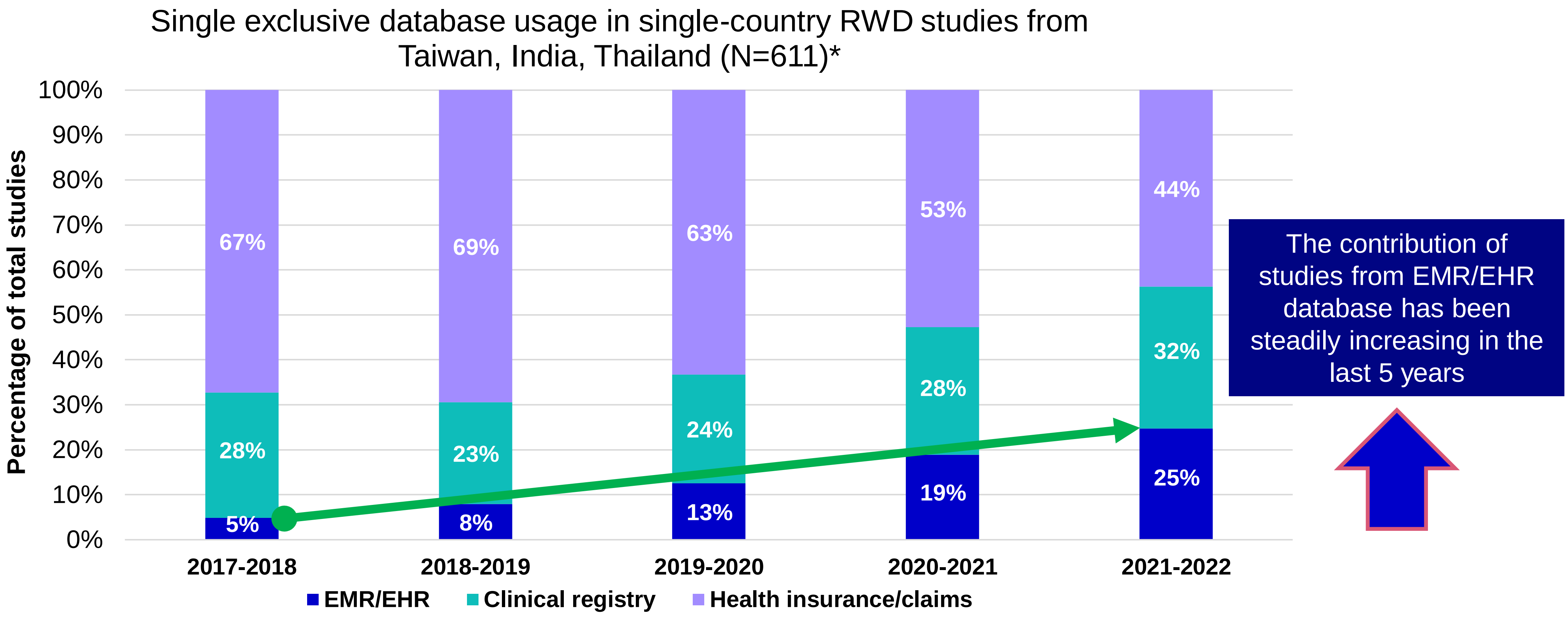

*Out of 745 studies in target countries, 611 has a single database type/source, while 134 has a combination of one or more database type/source.

Abbreviations: RWD, real-world data

Percentage of single exclusive database utilisation for RWD studies from cross-country collaboration studies of target countries

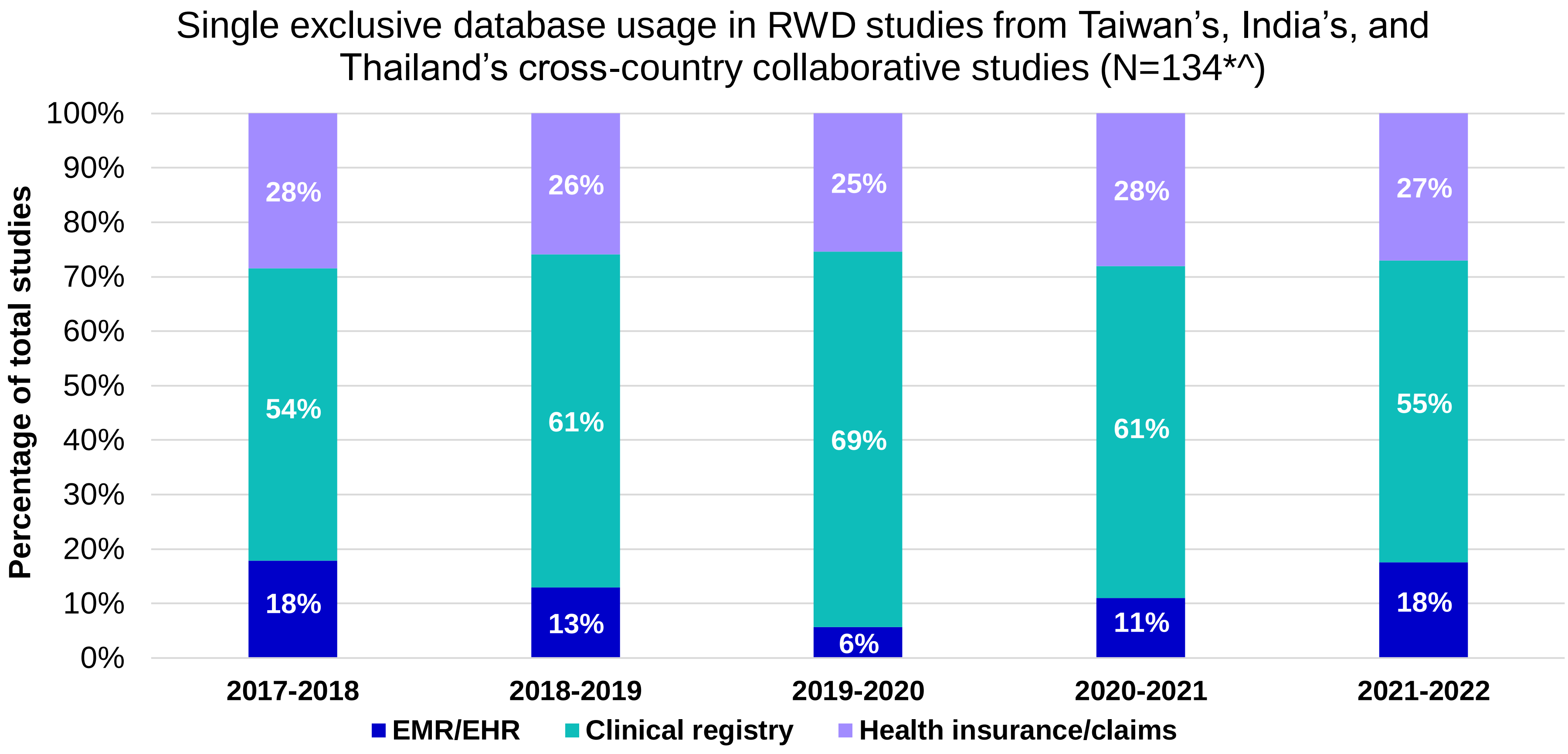

*Out of 134 studies in cross-country collaborative studies, 128 has a single database type/source, while 6 has a combination of one or more database type/source.

^Study numbers from cross-country studies may appear as duplicates for studies conducted in multiple target countries.

Abbreviations: RWD, real-world data

Study outcomes from integrated databases from single-country studies of target countries

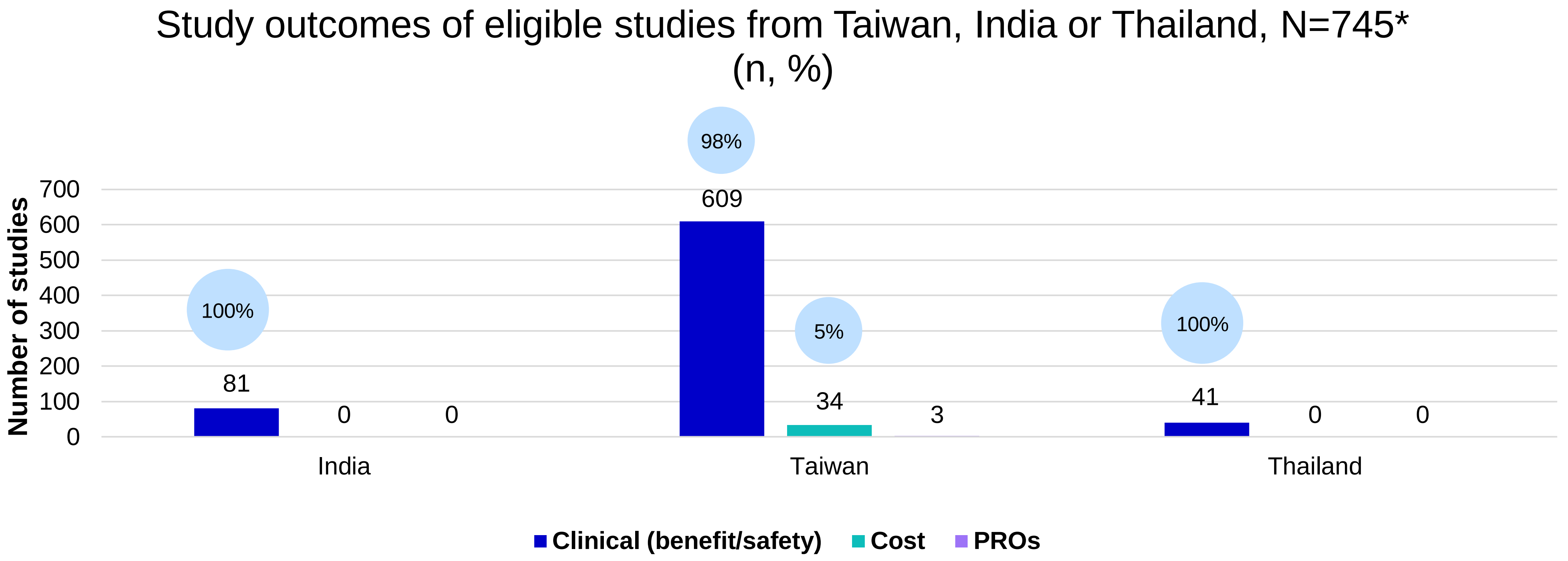

Study outcomes related to cost and PROs are incorporated only in RWD studies from Taiwan

*Study numbers may appear as duplicates for outcomes where >1 outcome was studied; hence the total percentage may not account for 100%.

Abbreviations: PROs, patient reported outcomes; RWD, real-world data

Study outcomes from integrated databases of cross-country collaborative studies

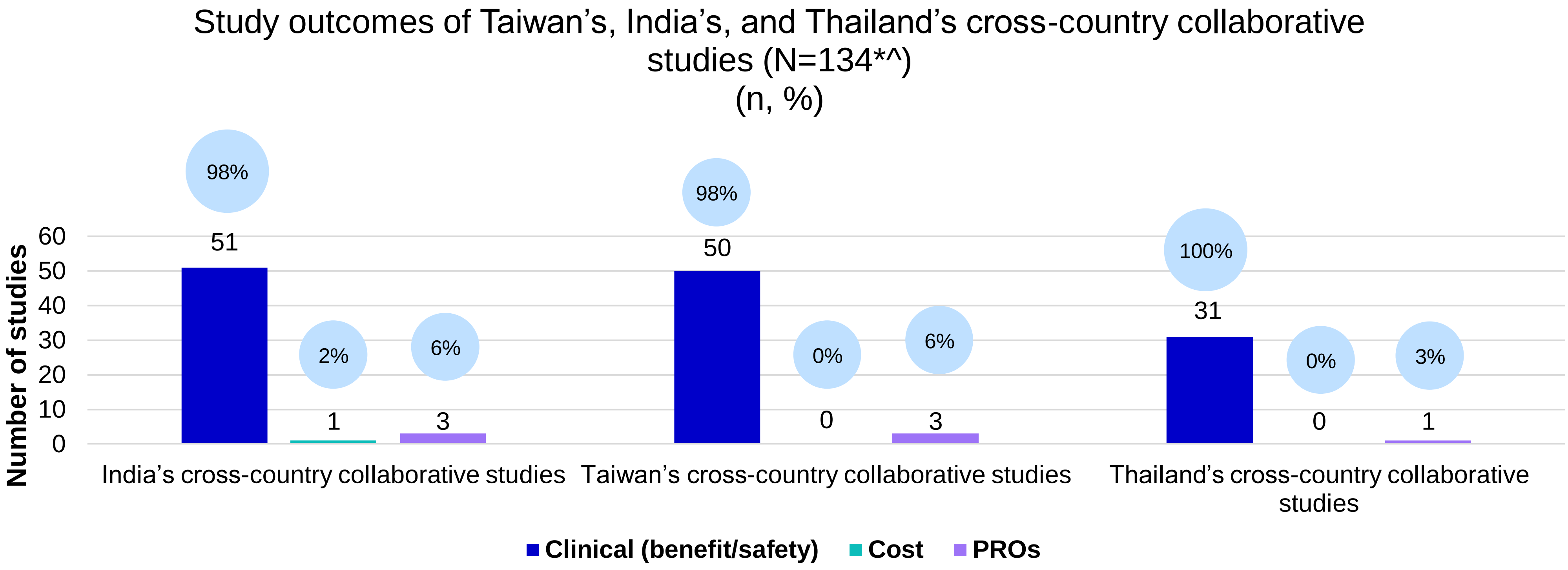

Study outcomes related to cost are incorporated only in RWD studies from India's cross-country collaborative studies

*Study numbers may appear as duplicates for outcomes where >1 outcome was studied; hence the total percentage may not account for 100%.
^Study numbers from cross-country studies may appear as duplicates for studies conducted in multiple target countries.

Abbreviations: PROs, patient reported outcomes; RWD, real-world data

Study duration from integrated databases of all included studies

Study Duration (Years)	Frequency	Cumulative %
2	78	10%
4	95	22%
6	99	34%
8	105	47%
10	102	60%
12	91	71%
14	112	85%
16	52	92%
18	40	97%
20	7	97%
>20	21	100%
Unknown	31	Excluded*

Descriptive Statistics	
Mean	9.4
Standard Error	0.2
Median	9.0
Mode	14.0
Standard Deviation	6.0
Sample Variance	36.2
Kurtosis	12.5
Skewness	2.0
Range	65.7
Minimum	0.1
Maximum	65.8
Sum	7533.1
Count	802.0

Histogram of Study Duration (N=833^)

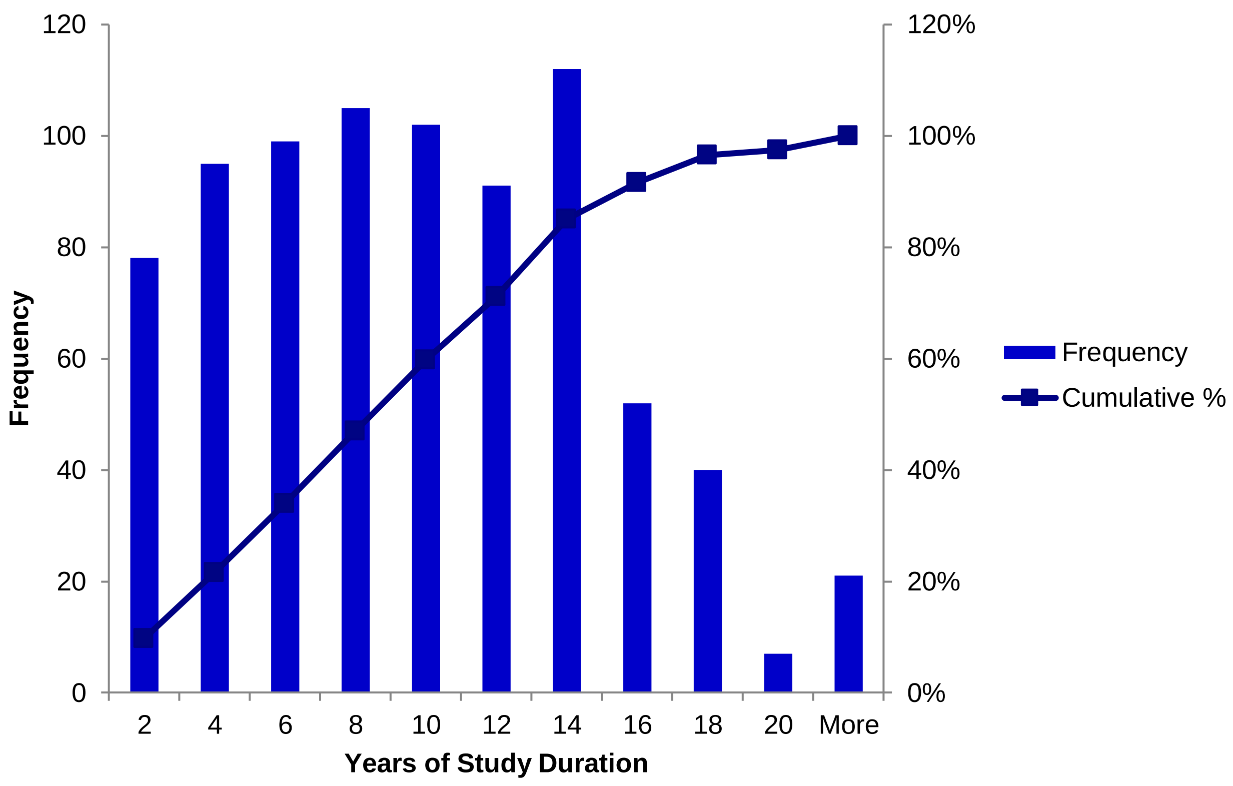

^Out of 833 studies, 802 have study duration while it was unknown for 31 studies

Study duration from integrated databases of three single-country studies from 2017-2022

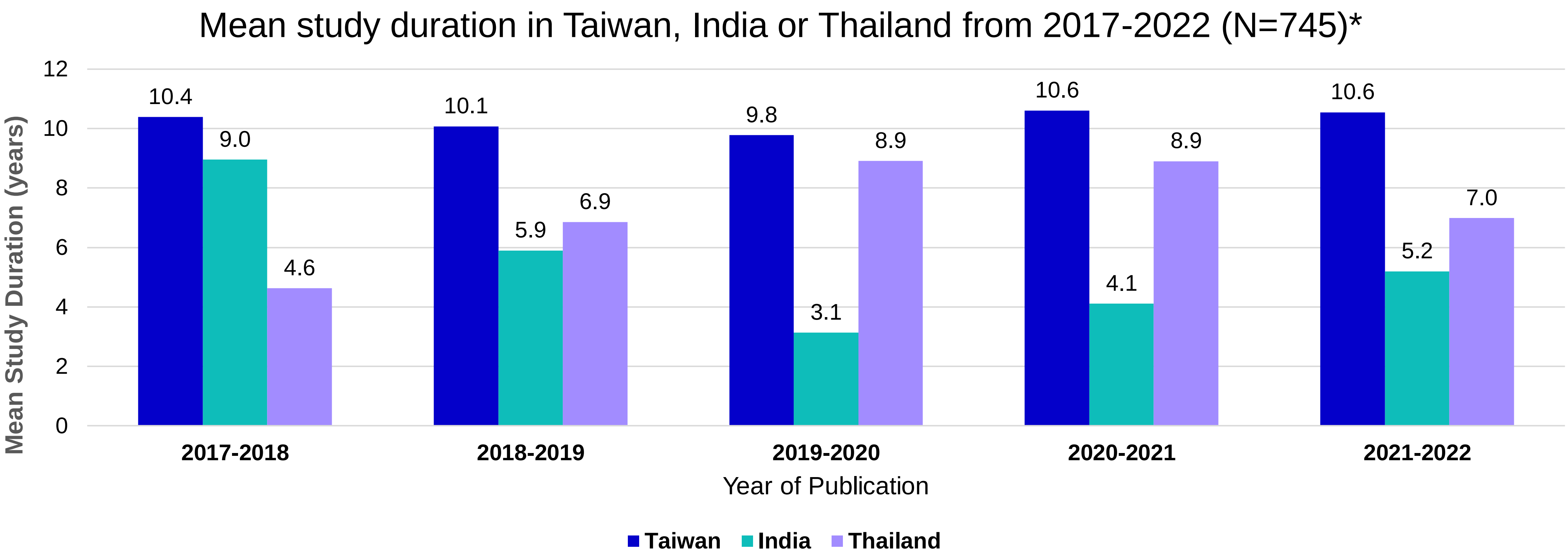

Mean study duration was almost constant for Taiwan at approximately 10 years.

*As the PubMed final search was conducted on September 27, 2022, with filter for last 5 years, the eligible studies do not contain full data from 2017 and 2022.

Study duration from integrated databases of cross-country collaborative studies from 2017-2022

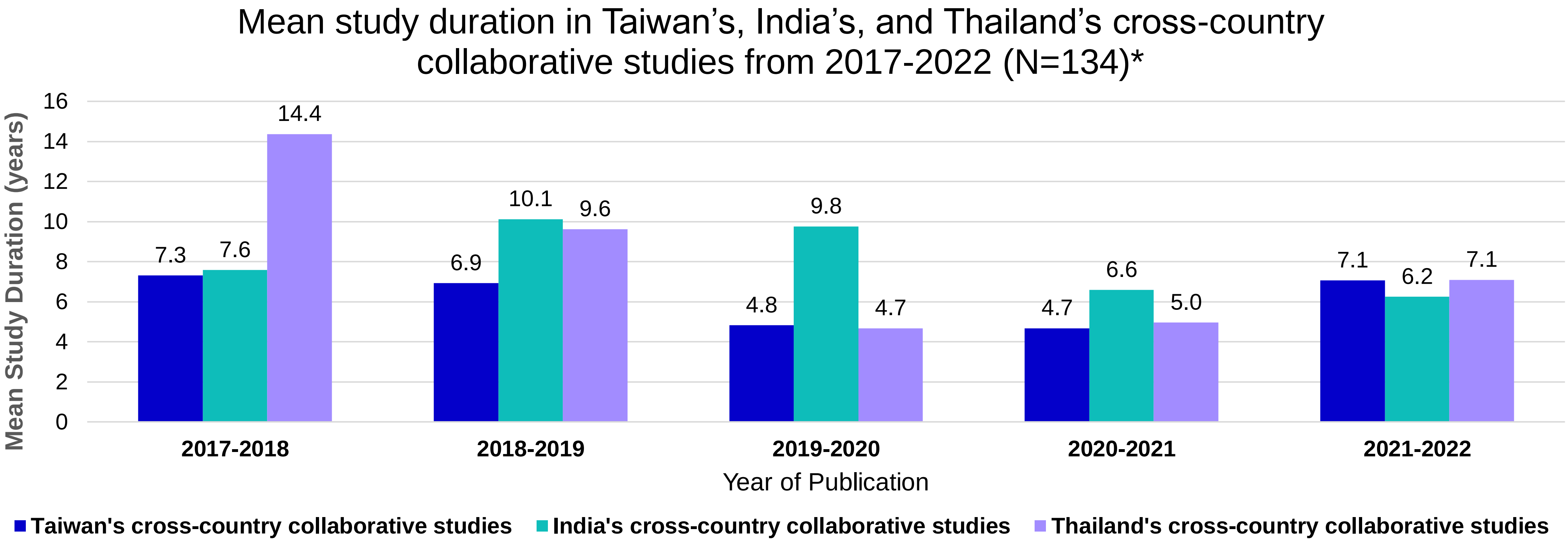

No trend in particular was observed across all cross-collaborative studies

*As the PubMed final search was conducted on September 27, 2022, with filter for last 5 years, the eligible studies do not contain full data from 2017 and 2022.

*Study numbers from cross-country studies may appear as duplicates for studies conducted in multiple target countries.

Publication time lag as per the number of RWD studies for all integrated databases

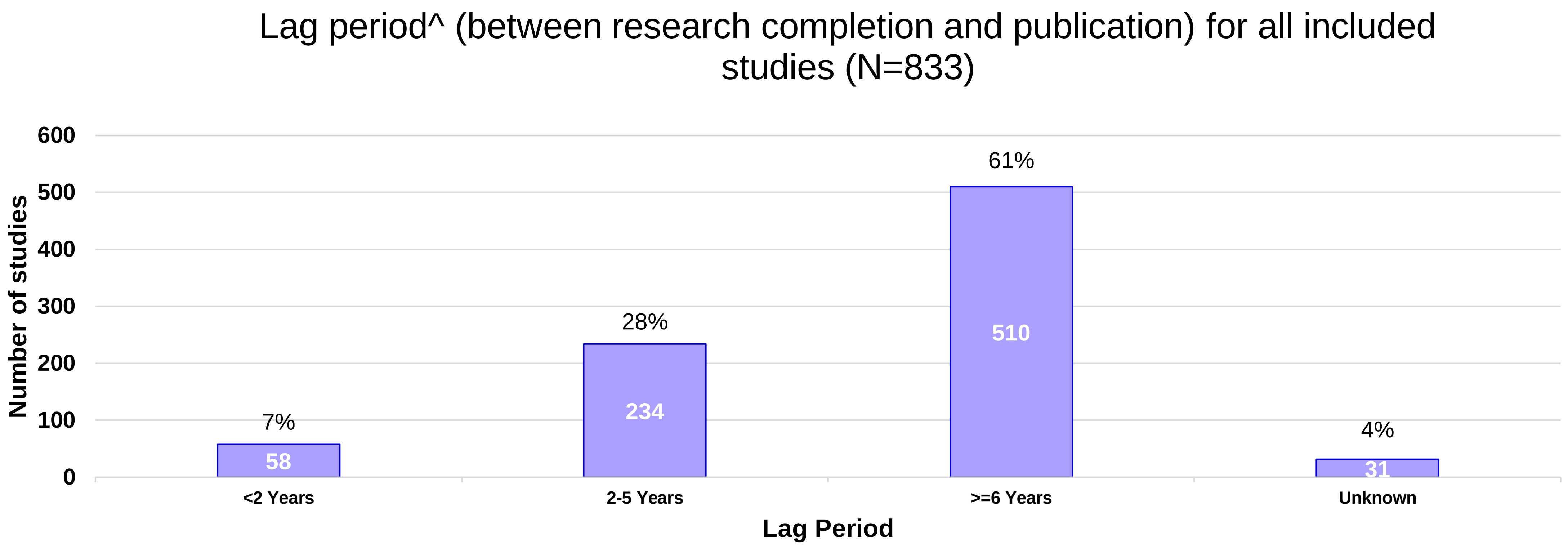

^The lag period is defined as follows:
<2 Years (for publications published within 2 years after the research completion),
2-5 Years (for publications published within 2 to 5 years after the research completion), >=6 Years (for publications published 6 years and above after the research completion).
Unknown is for publications with an unspecified year of the research completion.

Abbreviations: RWD, real-world data

Trend for publication time lag from the integrated databases of single-country studies from target countries from 2017-2022

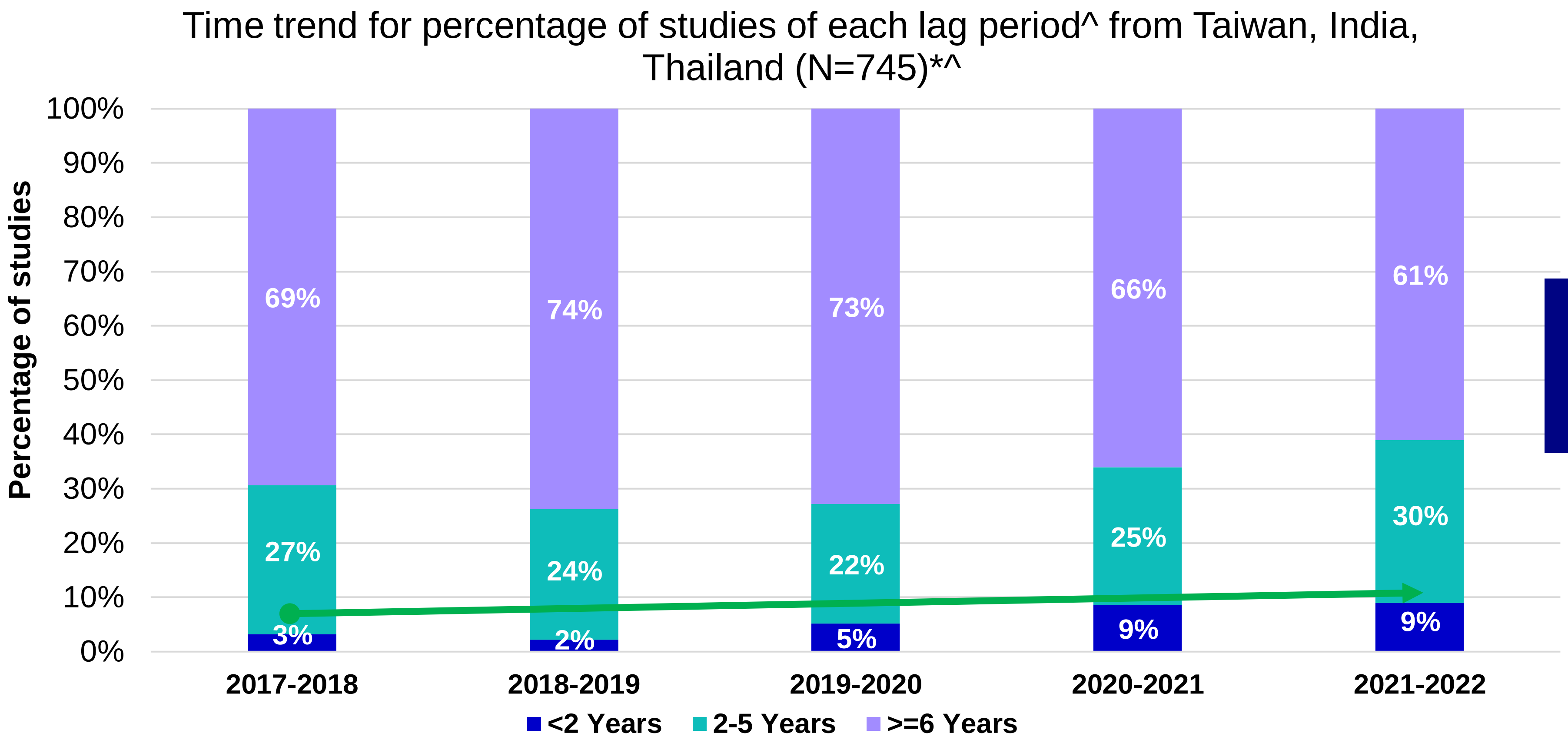

Rising trend in the number of studies being published within 2 years of their completion

*Out of 745 studies in target countries, 730 have a specified year of research completion, while 15 have an unspecified year of research completion.

^The lag period is defined as follows:
<2 Years (for publications published within 2 years after the research completion),
2-5 Years (for publications published within 2 to 5 years after the research completion), >=6 Years (for publications published 6 years and above after the research completion).

Trend for publication time lag from the integrated databases of cross-country collaborative studies from 2017-2022

Time trend for percentage of studies of each lag period^ from Taiwan's, India's, and Thailand's cross-country collaborative studies from 2017-2022 (N=134)*^

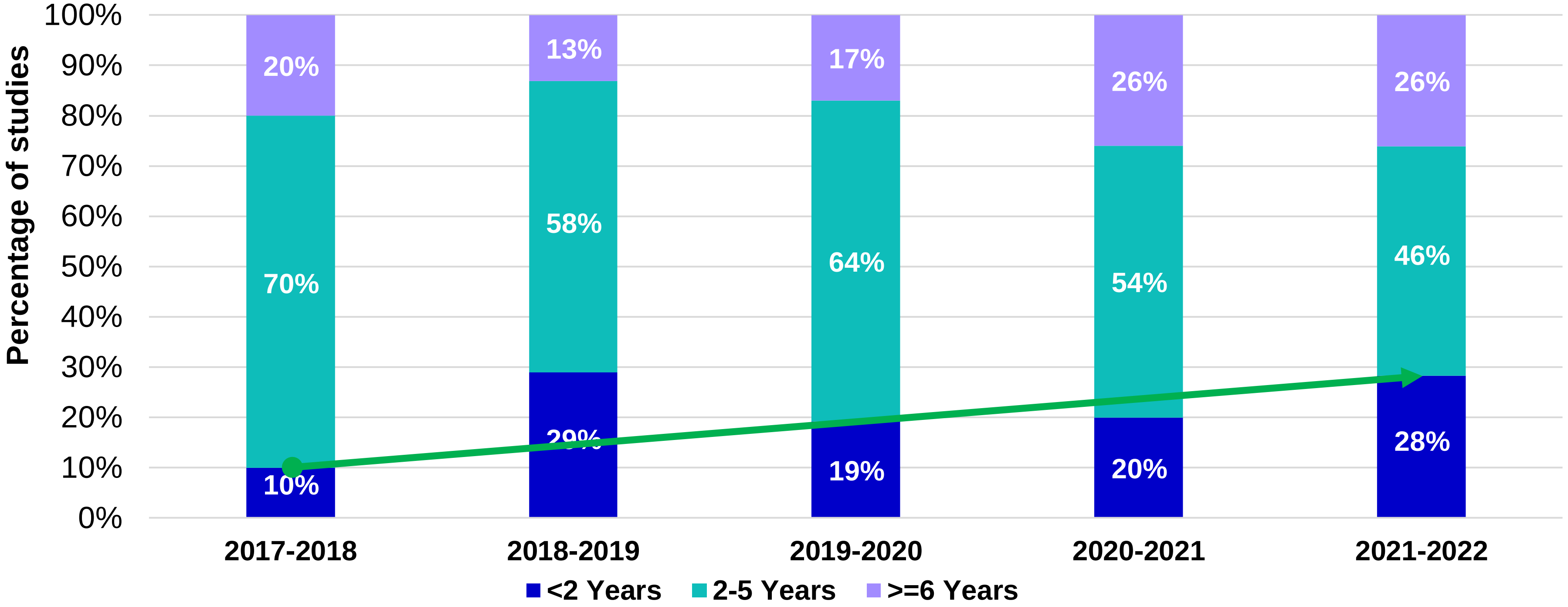

*Out of 134 studies in target countries, 113 have a specified year of research completion, while 21 have an unspecified year of research completion. Study numbers from cross-country studies may appear as duplicates for studies conducted in multiple target countries.

^The lag period is defined as follows:
<2 Years (for publications published within 2 years after the research completion),
2-5 Years (for publications published within 2 to 5 years after the research completion), >=6 Years (for publications published 6 years and above after the research completion).

Comparative analysis of publication speed for single-country studies from single exclusive databases

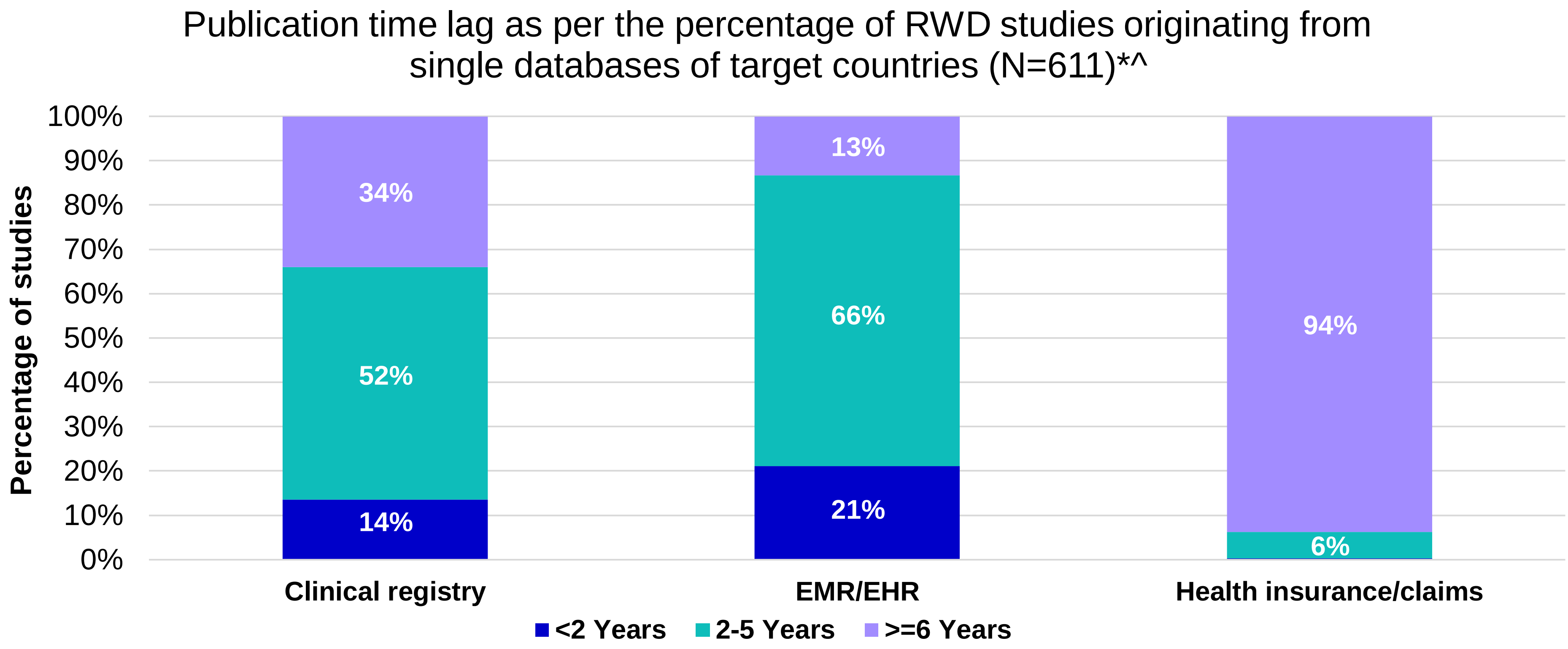

*Out of 745 studies in target countries, 611 used a single database type/source, while 134 utilised a combination of one or more database types/sources.
Out of 611 studies with a single database type/source, 598 have a specified year of research completion, while 13 have an unspecified year of research completion.

^The lag period is defined as follows:
<2 Years (for publications published within 2 years after the research completion),
2-5 Years (for publications published within 2 to 5 years after the research completion), >=6 Years (for publications published 6 years and above after the research completion).

Comparative analysis of publication speed for cross-country collaborative studies from single exclusive databases

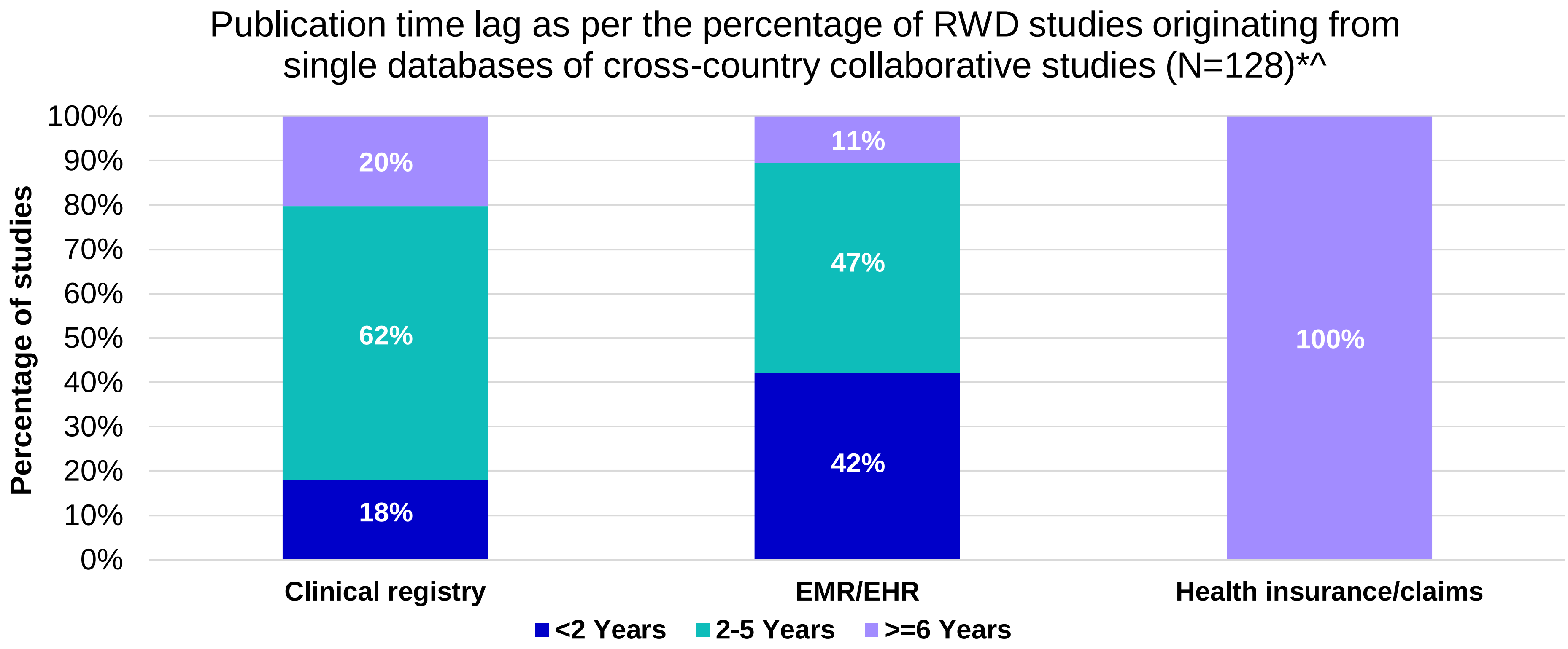

*Out of 134 cross-country collaborative studies in target countries, 128 used a single database type/source, while 6 utilised a combination of one or more database types/sources. Out of 128 studies with a single database type/source, 109 have a specified year of research completion, while 19 have an unspecified year of research completion.

^The lag period is defined as follows:
<2 Years (for publications published within 2 years after the research completion),
2-5 Years (for publications published within 2 to 5 years after the research completion), >=6 Years (for publications published 6 years and above after the research completion).

Study sample size from integrated databases for all included studies

Sample Sizes	Frequency	Cumulative %
10	0	0%
100	14	2%
1000	123	17%
10000	270	50%
100000	263	82%
1000000	111	96%
>1 M	32	100%
Unknown	20	Excluded*

Descriptive Statistics	
Mean	352,814
Standard Error	192,814
Median	9,831
Mode	105
Standard Deviation	5,497,745
Sample Variance	3.E+13
Kurtosis	764
Skewness	27
Range	154,499,980
Minimum	20
Maximum	154,500,000
Sum	286,838,010
Count	813

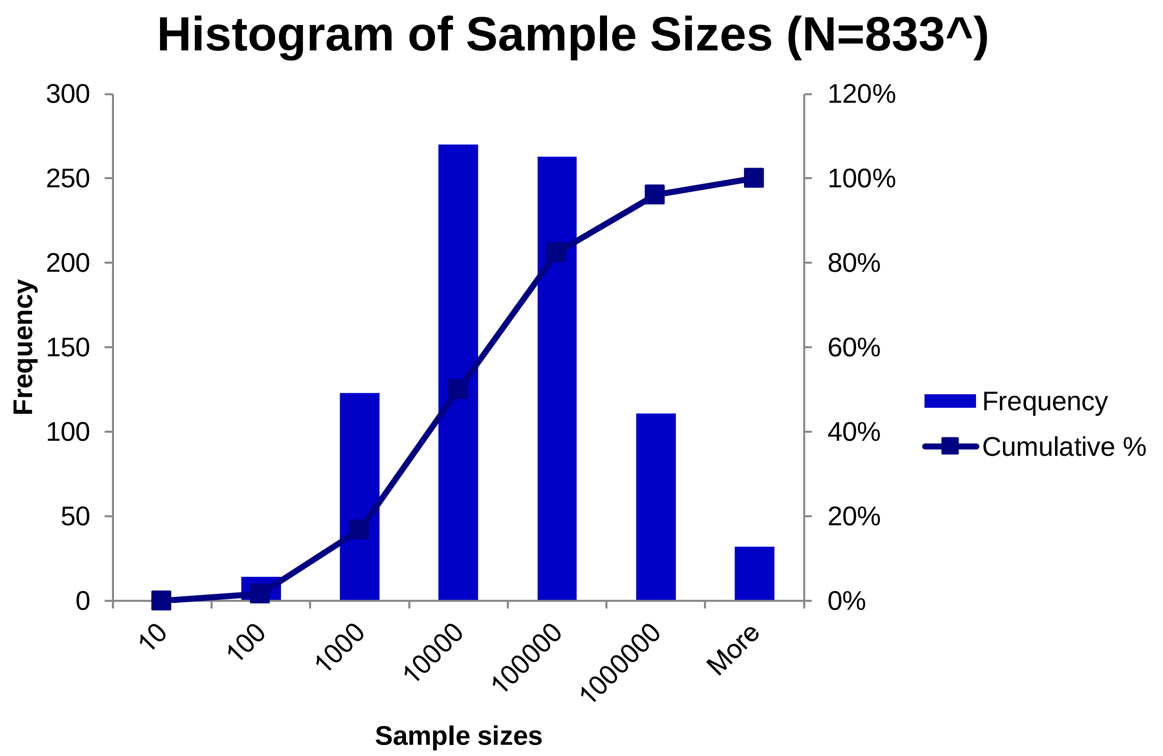

^Out of 833 studies, 813 studies have data on sample sizes, while it was unknown for 20 studies.

Sample size from integrated databases of three target countries from 2017-2022

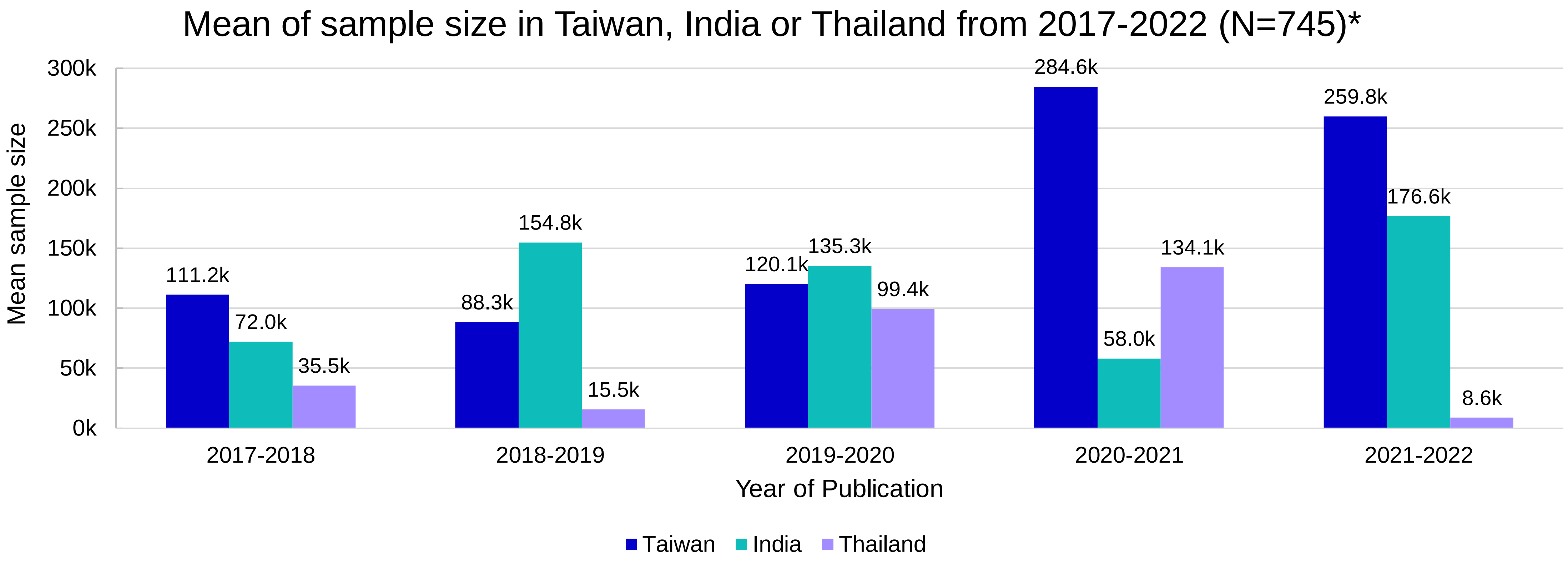

*As the PubMed final search was conducted on September 27, 2022, with filter for last 5 years, the eligible studies do not contain full data from 2017 and 2022.

Abbreviations: k, thousand; RWD, real-world data

Number of study centres from integrated databases for all included studies

Number of Centres	Frequency	Cumulative %
10	102	48%
20	30	62%
30	18	70%
40	16	78%
50	11	83%
60	6	86%
70	2	86%
80	2	87%
90	0	87%
100	1	88%
>100	26	100%
Unknown	619	Excluded*

Descriptive Statistics	
Mean	106
Standard Error	26
Median	13
Mode	7
Standard Deviation	378
Sample Variance	143008
Kurtosis	36
Skewness	6
Range	2744
Minimum	2
Maximum	2746
Sum	22579
Count	214

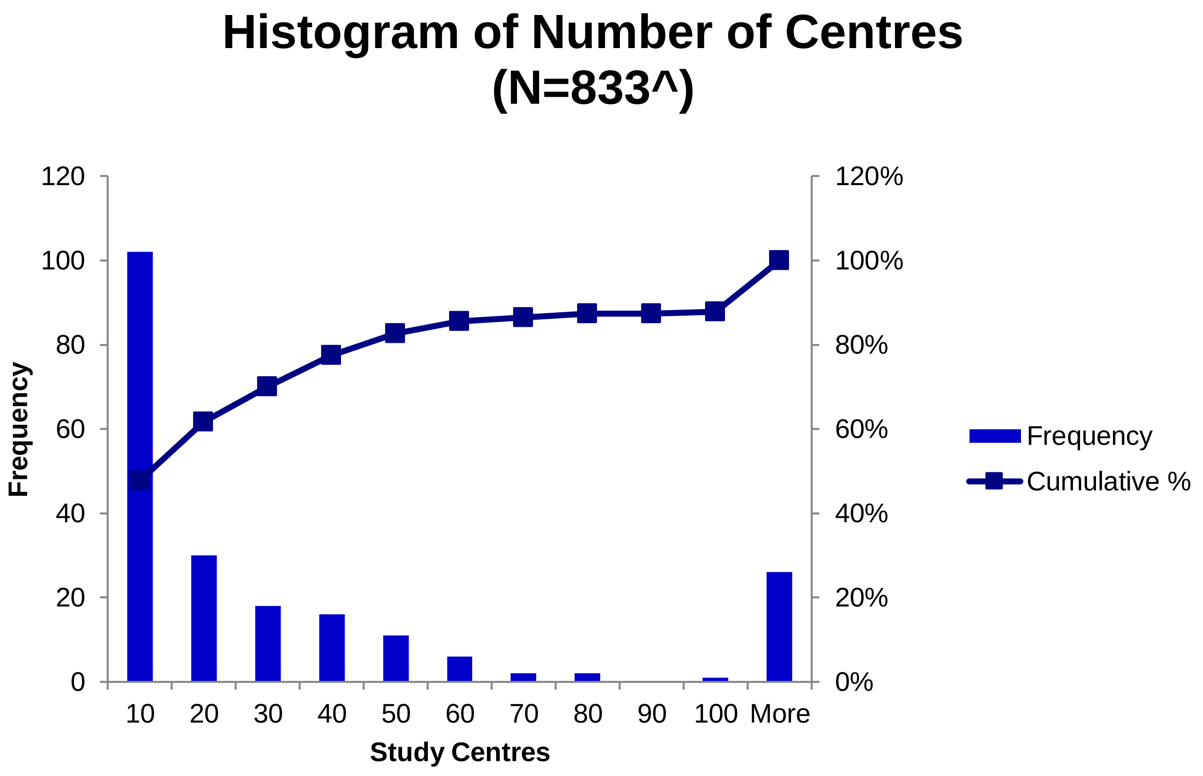

Most studies did not explicitly report the number of centers involved, followed by 10, 20 and >100 study centers

^Out of 833 studies, 214 have information on the number of centres, while it was unknown in 619 studies.

Study centers from integrated databases of three target countries from single-country studies from 2017-2022

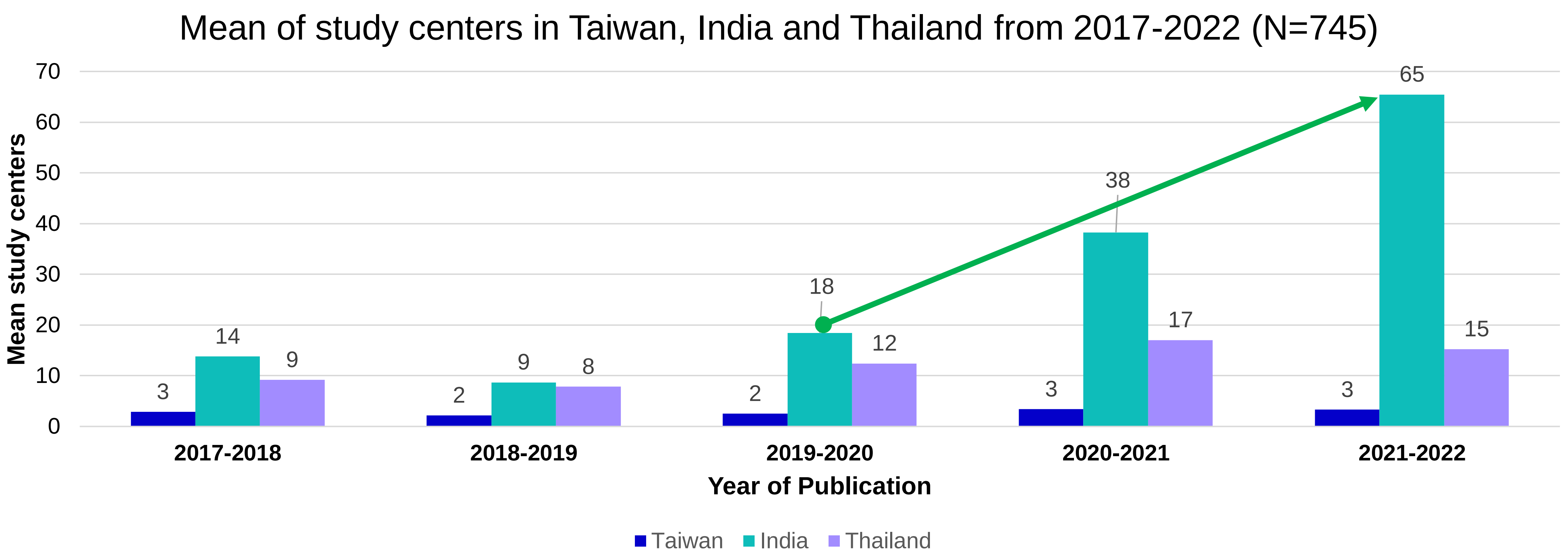

*As the PubMed final search was conducted on September 27, 2022, with filter for last 5 years, the eligible studies do not contain full data from 2017 and 2022.

Study centers from integrated databases of cross-country collaborative studies from 2017-2022

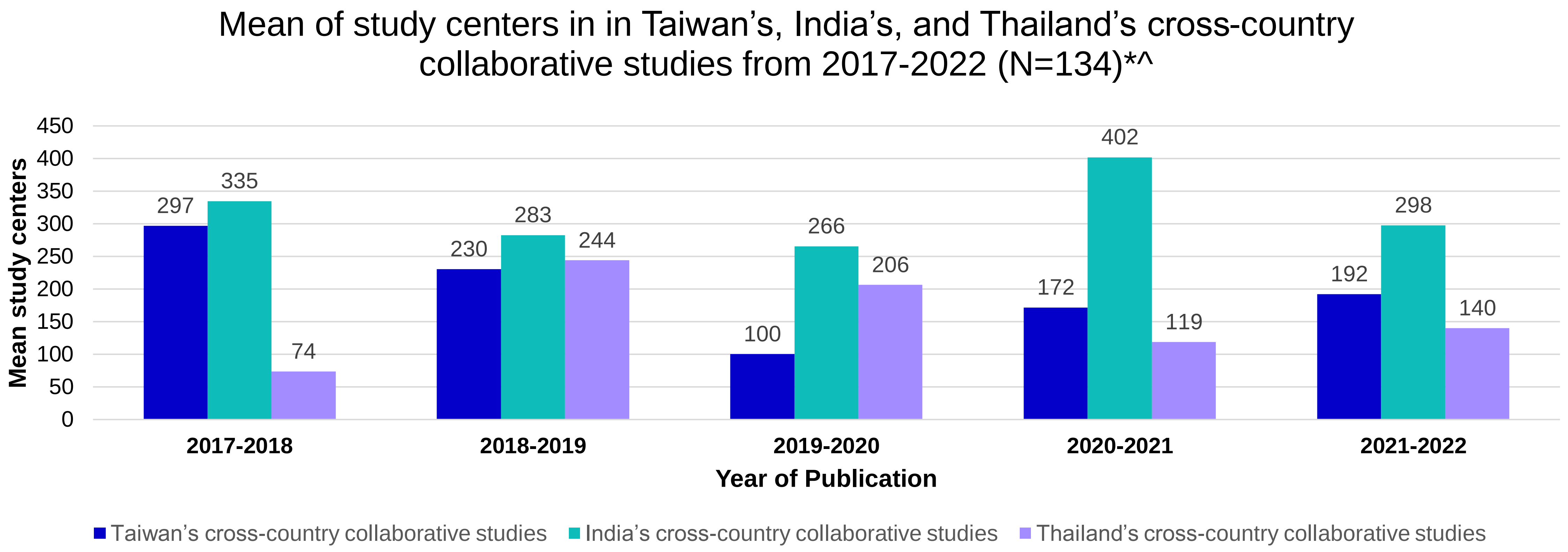

*As the PubMed final search was conducted on September 27, 2022, with filter for last 5 years, the eligible studies do not contain full data from 2017 and 2022.

^Study numbers from cross-country studies may appear as duplicates for studies conducted in multiple target countries.
