# Supplementary material for: Integrated Real-World Study Databases in 3 Diverse Asian Health Care Systems in Taiwan, India, and Thailand: Scoping Review
Source: J Med Internet Res. 2023 Sep 11;25:e49593. doi: 10.2196/49593 (PMC10520767; doi:10.2196/49593)
Supplement: Multimedia Appendix 5 [file jmir_v25i1e49593_app5.pdf]

Multimedia Appendix 5

Scoping review to identify and describe integrated contemporary real-world studies databases from three diverse healthcare systems in Asia: Taiwan, India, and Thailand

Wen-Yi Shau, Sajita Setia, Ying-Jan Chen, Tsu-yun Ho, Salil Shinde, Handoko Santoso, Daniel Furtner

International Registered Report Identifier (IRRID): RR2-10.2196/43741

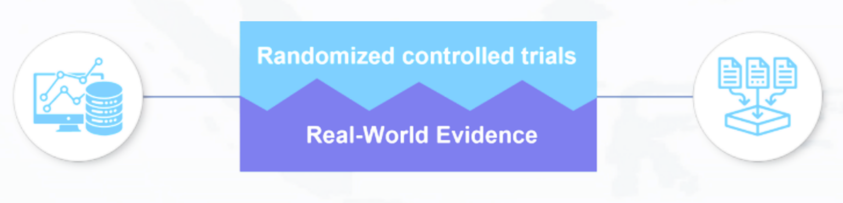

TOPLINE RESULTS

Real-world studies originating from contemporary integrated databases

Databases identified names as per disease area

Scoping review for Taiwan, India, and Thailand

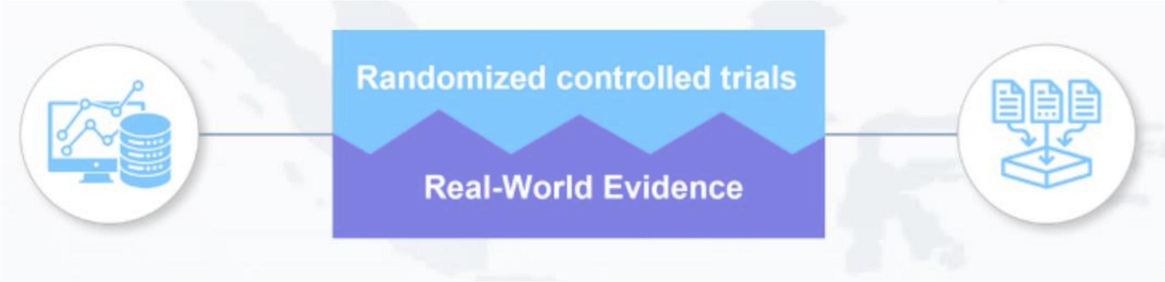

TOPLINE RESULTS

Real-world studies originating from contemporary integrated databases

Databases identified names from Taiwan

Scoping review for India, Thailand, and Taiwan

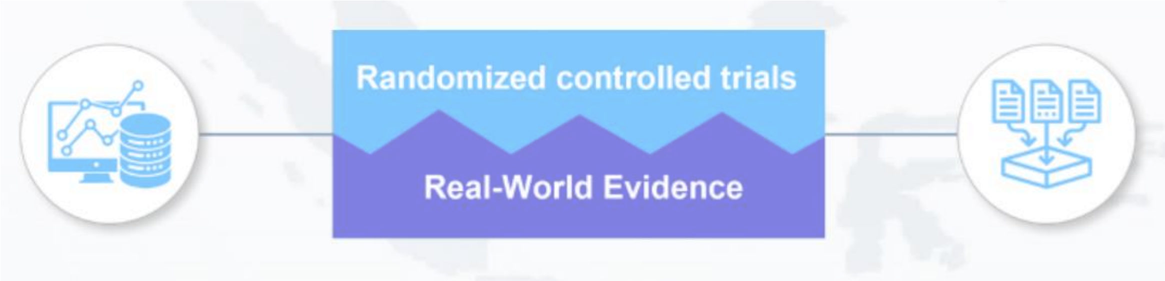

Unique names of identified clinical registries databases from Taiwan (1)

Identified registry databases from oncology disease area from Taiwan (N=43^)

Database Type	Database Name	Disease Area	Study Details
Clinical registry	Taiwan upper urinary tract urothelial carcinoma Collaboration database	Oncology (1)	Lo 2022
	Taiwan Cancer Registry		Liang 2020
	Taiwan Cancer Registry		Yang 2021
	Taiwan Cancer Registry		Chang 2017
	Taiwan Cancer Registry		Li 2021
	Taiwan Cancer Registry		Fang 2022
	Taiwan Cancer Registry; Taiwan Death registry		Chen 2018
	Taiwan Cancer Database; Taiwan Cancer Registry		Chen 2021
	Taiwan Cancer Registry		Lin 2018
	Taiwan Oral Mucosal Screening (TOMS) program; Taiwan Cancer Registry (TCR) and Taiwan Death Registry (TDR)		Ho 2019
	Taiwan Cancer Registry		Tseng 2022
	Taiwan Cancer Registry		Chen 2022
	Taiwan Cancer Registry; Taiwan Death Registry		Chao 2022
	Taiwan Cancer Registry		Chen 2021
	Taiwan Cancer Registry		Chen 2022
	Taiwan Cancer Registry		Kuo 2020
	Taiwan Cancer Registry		Lin 2018
	Taiwan Cancer Registry		Chang 2020
	Taiwan Cancer Registry		Shih 2021
	Taiwan Cancer Registry		Tsai 2021
	National Cancer Registration system; Taiwan Cancer registry		Hsieh 2017

^Out of 43 studies based on clinical registries with oncology disorders, databases name are available in 41 studies while 2 studies do not mention any particular names for databases.

Unique names of identified clinical registries databases from Taiwan (2)

Identified registry databases from oncology disease area from Taiwan (N=43^)

Database Type	Database Name	Disease Area	Study Details
Clinical registry	Taiwan Cancer Registry	Oncology (2)	Wang 2019
	Taiwan Cancer Registry		Liu 2020
	Taiwan Cancer Registry		Zhang 2020
	Taiwan upper tract urothelial carcinoma registry		Chen 2021
	Taiwan Cancer Registry		Chao 2017
	Taiwan Bone Marrow Transplant Registry database		Wang 2022
	Registry for Catastrophic Illness Patient Database (RCIPD)		Hu 2017
	Taiwan Cancer Registry		Zhang 2020
	Taiwan Cancer Registry		Liu 2021
	Taiwan Cancer registry		Cheng 2018
	Taiwan Blood and Marrow Transplantation Registry		Huang 2019
	Taiwan Health and Welfare Data Science (HWDS); Taiwan Cancer Registry(TCR); Taiwan Death Registry (TDR)		Nguyen 2020
	Taiwan immune checkpoint inhibitors registry database		Huang 2020
	Taiwan Cancer Registry		Chang 2018
	Taiwan Cancer Registry		Lin 2021
	Taiwan Cancer Registry		Hsu 2020
	Taiwan Cancer Registry		Wang 2020
	Taiwan Cancer Registry		Hu 2021
	Taiwan Cancer Registry		Chuang 2020
	Taiwan Society of Cancer Registry (TSCR)		Wang 2020

^Out of 43 studies based on clinical registries with oncology disorders, databases names are available in 41 studies, while 2 studies do not mention any particular names for databases.

Unique names of identified clinical registries databases from Taiwan (3)

Identified registry databases from cardiology and metabolic disorders disease area from Taiwan (N=27^)

Database Type	Database Name	Disease Area	Study Details
**Clinical registry**	Taiwan Society of Cardiology-Heart Failure with reduced Ejection Fraction (TSOC-HFrEF) registry	**Cardiology and metabolic disorders**	Fong 2021
	Taiwan Society of Cardiology-Heart Failure with reduced Ejection Fraction (TSOC-HFrEF) registry		Chang 2022
	Taiwan Stroke Registry		Yang 2018
	Taiwan Cancer Registry		Lin 2018
	Taiwan Primary Aldosteronism Investigation (TAIPAI) database		Wu 2022
	Taiwan Network of Targeted Temperature Management for Cardiac Arrest (TIMECARD) registry		Chou 2022
	Taiwan Stroke Registry		Wang 2022
	Taiwan Society of Cardiology Registry-heart failure with reduced ejection fraction (TSOC-HFrEF Registry)		Chen 2021
	Hunter Outcome Survey, a global, multicentre registry that collects real-world data on patients with MPS II		Lin 2018
	Taiwan Primary Aldosteronism Investigation (TAIPAI) registry		Wu 2019
	Cardiovascular Atherosclerosis and Percutaneous TrAnsluminal INterventions (CAPTAIN) registry		Hsieh 2019
	Taipei out-of-hospital cardiac arrest (OHCA) registry		Lin 2022
	Taiwan network of targeted temperature ManagEment for CARDiacarrest (TIMECARD) registry		Lin 2022
	Taiwan Cohort-Registry of chronic thromboembolic pulmonary hypertension or CTEPH (TREC)		Liu 2022
	Acute Coronary Syndrome-Diabetes Mellitus (ACS-DM) Registry of the Taiwan Society of Cardiology (TSOC)		Jong 2019
	Taiwan Secondary Prevention for patients with AtheRosCLErotic disease (T-SPARCLE) registry		Yeh 2017
	Taiwan Stroke Registry		Qureshi 2019
	Taichung OHCA registry		Yu 2021
	Taiwan network of targeted temperature ManagEment for CARDiac arrest (TIMECARD) registry		Chien 2021
	Acute Coronary Syndrome-Diabetes Mellitus Registry of the Taiwan Society of Cardiology (TSOC ACS-DM) Registry		Wang 2020
	Nationwide ACS (acute coronary syndrome) registry by the Taiwan Society of Cardiology		Li 2019
	Taiwan Diabetes Registry		Wang 2021
	Utstein-based registry system of patients with out-of-hospital cardiac arrest from a Taipei emergency medical service (EMS)		Chiang 2018
	Out-of-hospital cardiac arrest (OHCA) database in Taoyuan City		Chien 2020
	Cardiovascular Atherosclerosis and Percutaneous TrAnsluminal INterventions (CAPTAIN) registry		Hsieh 2018
	Taiwan cohort - Registry of CTEPH (TREC)		Liu 2021

^Out of 27 studies based on clinical registries with cardiology and metabolic disorders, databases name are available in 26 studies, while 1 study does not mention any particular name for databases.

Unique names of identified clinical registries databases from Taiwan (4)

Identified registry databases from infectious diseases and vaccines & inflammatory and autoimmune disorders area from Taiwan (N=11)

Database Type	Database Name	Disease Area	Study Details
**Clinical registry**	Taiwanese chronic hepatitis C cohort (T-COACH)	**Infectious diseases and vaccines**	Hsu 2021
	Taiwan Association for the Study of the Liver HCV Registry		Cheng 2022
	Taiwan HCV Registry		Huang 2022
	Dengue Disease Reporting System		Yeh 2017
	Taiwan Association for the Study of the Liver HCV Registry (TACR)		Lo 2022
	Taiwan HCV Registry (TACR)		Chen 2021
	TACR		Huang 2021
	BATTLE registry	**Inflammatory and autoimmune disorders**	Wang 2022
	BATTLE registry		Huang 2022
	Registry for Catastrophic Illness from the National Health Research Institute (NHRI)		Huang 2018
	Taiwan Rheumatology Association Clinical Electronic Registry (TRACER)		Lin 2021

Unique names of identified clinical registries databases from Taiwan (5)

Identified registry databases from Others area from Taiwan (N=27^)

Database Type	Database Name	Disease Area	Study Details
**Clinical registry**	Pre-end-stage renal disease (ESRD) care registry	**Others**	King 2021
	Chang Gung Trauma Registry Programme		Cheng 2020
	Taipei City Elderly Health Examination Database; National Death Registry; Taipei Geriatric Health Examination Database		Huang 2021
	Chang Gung Memorial Hospital Trauma Registry System		Wu 2020
	Taiwan Renal Registry Data System		Liu 2021
	Taiwan Blood Services Foundation donor database		Lu 2022
	Chang Gung Stroke Registry database		Lin 2017
	Taiwan Renal Registry Data System		Liao 2021
	OCEAN (Obesity and Clock for Elegant AgiNg, NCT02674230) registry		Wang 2020
	Taiwan Renal Registry Data System (TWRDS)		Su 2017
	Taiwan stroke registry (TSR)		Wang 2018
	Trauma Registry System		Rau 2017
	Taiwan Renal Registry Data System (TWRDS)		Lin 2018
	National Health Insurance Research Database		Macerollo 2019
	Nationwide registry for patients receiving hematopoietic stem cell transplantation (HSCT) by the Taiwan Society of Blood and Marrow Transplantation		Lee 2019
	Taiwan Central Cytogenetic Registry of the Health Promotion Administration register center; National Birth Defect Registration and Notification system		Hsiao 2022
	Taiwan Stroke Registry (TSR)		Wen 2017
	Taiwan Renal Registry Data System		Yang 2017
	Taiwan Stroke Registry		Wang 2018
	Taiwan registry		Ko 2021
	Taiwanese Registry of Epilepsy and Pregnancy (TREP)		Yeh 2017
	National Birth Reporting Database; National Birth Certification Registry; National Death Certification Registry		Liang 2018
	Taiwan Birth Registry; Taiwan Death Registry		Lee 2018
	National OAT registry; National Mortality Registry		Chang 2019
	Taiwan Stroke Registry		Chi 2018
	Taiwan Bariatric Registry of Taiwan Society Metabolic and Bariatric Surgery		Chang 2021
	Tainan Incident Registry System		Pan 2019

Unique names of identified mixed clinical registries and claims databases from Taiwan (1)

Identified Clinical registry; Health insurance/claims databases from multiple disease areas from Taiwan (N=18)

Database Type	Database Name	Disease Area	Study Details
**Clinical registry; Health insurance/claims**	National Health Insurance Research Database; Longitudinal Cohort of Diabetes Patients (LHDB 2000)	**Cardiology**	Chou 2020
	National Health Insurance Research Database; National Mortality Registry		Chueh 2022
	National Health Insurance program; National Register of Deaths in Taiwan		Chang 2018
	NHI claims database; Taiwan National Death Registry		Lai 2021
	Claim database of Taiwan's National Health Insurance; National Mortality Registry Database		Liao 2021
	Birth Certificate Application database (BCA); National Health Insurance Research Database		Su 2021
	National Health Insurance (NHI); National Death Registry		Lin 2018
	National Health Insurance Research Database; National Death Registry		Li 2019
	National Health Insurance (NHI); Taiwan Cancer Registry (TCR); Taiwan Death Registry (TDR)	**Infectious disease and vaccines**	Liu 2021
	Longitudinal Health Insurance Database 2000, a subset of the National Health Insurance Research Database; Cancer Registry		Lee 2021
	Taiwanese chronic Hepatitis C cohort; National Health Insurance Research Database		Hsu 2020
	National Health Insurance Research Database; National Death Registry		Lee 2019
	National TB Registry; National Health Insurance Research Database		Chen 2019
	Study on Taiwanese Chronic Hepatitis C Cohort (T-COACH); Registry for Catastrophic Illness; Taiwan Cancer Registry		Huang 2020
	National vaccination registry system (National Immunization Information System); National Health Insurance Database		Cheng 2018
	Longitudinal Health Insurance Database 2000 (LHID2000) a subset of the National Health Insurance Research Database; Registry for Catastrophic Illness Patients;	**Inflammatory and autoimmune disorders**	Chang 2020
	Registry of the National Notifiable Disease Reporting System; National Health Insurance Research Database		Chang 2021
	National Health Insurance Research Database (NHIRD); Death Registry		Hou 2022

Unique names of identified mixed clinical registries and claims databases from Taiwan (2)

Identified Clinical registry; Health insurance/claims databases from oncology disease area from Taiwan (N=64)

Database Type	Database Name	Disease Area	Study Details
Clinical registry; Health insurance/claims	National Health Insurance Research Database; Cancer Registry Database	Oncology (1)	Wu 2022
	Taiwan Cancer Registry (TCR) ; National Health Insurance (NHI) Research Database (NHIRD); National Death Registry (NDR)		Hsiao 2022
	National Health Insurance Research Database (NHIRD); Taiwan Cancer Registry (TCR); Cause of Death database		Tsai 2022
	Taiwan Cancer Registry; National Health Insurance Research Database; Taiwan Death Registry		Lee 2019
	Taiwan Cancer Registry; National Health Insurance Research Database (NHIRD); Death Registry		Huang 2021
	Taiwan cancer registry (TCR); death registration; National Health Insurance (NHI)		Lin 2022
	National Health Insurance Research Database; Registry for Catastrophic Illness Patients; Taiwan Cancer Registry Database.		Sun 2022
	Taiwan Cancer Registry Database, National Health Insurance Research Database		Chen 2021
	National Health Insurance Research Database Taiwan Cancer Registry		Hsieh 2022
	Taiwan Cancer Registry (TCR); Taiwan death registry; reimbursement data from National Health Insurance (NHI); data for the whole Taiwan population via Health and Welfare Data Science Center (HWDC) database		Li 2019
	Taiwan Cancer Registry Database (TCRD); Taiwan National Insurance Research Data (NHIRD); National Death Registry (NDR)		Pan 2022
	National Health Insurance Claim Database; Taiwan Cancer Database (TCDB) registry		Wang 2021
	Taiwan Cancer Registry (TCR); death registry; National Health Insurance (NHI)		Kuo 2019

Unique names of identified mixed clinical registries and claims databases from Taiwan (3)

Identified Clinical registry; Health insurance/claims databases from oncology disease area from Taiwan (N=64)

Database Type	Database Name	Disease Area	Study Details
**Clinical registry; Health insurance/claims**	Taiwan Cancer Registry Database (TCRD) ; Taiwanese National Health Insurance Research Dataset (TNHIRD)	**Oncology (2)**	Liao 2022
	Taiwan Cancer Registry; National Health Insurance Research Database; Death Registry		Li 2022
	Health and Welfare Data Science Center (HWDC); Taiwan Cancer Registry; death registration and reimbursement data for the whole Taiwan population provided by the Bureau of National Health Insurance (NHI)		Liang 2020
	National Health Insurance Research Database; Taiwan Cancer Registry; Catastrophic Illness Patient Registry; Cause of Death Data of Taiwan		JerryTeng 2022
	Taiwan Cancer Registry Database; Health and Welfare Database		Lee 2020
	Taiwan Cancer Registry (TCR); the National Health Insurance Research Database (NHIRD)		Chung 2022
	National Health Insurance database; National Death Registry; Taiwan Cancer Registry		Chen 2022
	Taiwan Cancer Registry (TCR); National Health Insurance Research database (NHIRD)		Chu 2021
	Taiwan Cancer Registry (TCR); Taiwan National Health Insurance (NHI) Research Database; National Death Registry database		Liang 2017
	National Health Insurance Claim Database, Taiwan Cancer Registry database		Chen 2021
	Taiwan Cancer Registry; National Health Insurance claims database		Lee 2019
	Taiwan Cancer Registry; database of labour insurance		Lee 2018
	Taiwan Cancer Registry Database (TCRD); Taiwan National Health Insurance Research Database (NHIRD)		Wu 2021

Unique names of identified mixed clinical registries and claims databases from Taiwan (4)

Identified Clinical registry; Health insurance/claims databases from oncology disease area from Taiwan (N=64)

Database Type	Database Name	Disease Area	Study Details
**Clinical registry; Health insurance/claims**	Taiwan cancer registry (TCR); death registry; National Health Insurance (NHI)	**Oncology (3)**	Li 2019
	Taiwan Cancer Registry Database (TCRD); National Health Insurance Research Database(NHIRD); National Death Registry		Huang 2019
	Cancer Registry; National Health Insurance Research Database (NHIRD); Death Registry		Ho 2018
	Taiwan Cancer Registry; Taiwan Death Registry; National Health Insurance Research Database (NHIRD)		Tsai 2018
	National Health Insurance Research Database; Taiwan Cancer Registry; Death Registry		Wu 2019
	National Health Insurance Research Database; Taiwan Cancer Registry Database		Tang 2022
	Taiwan Cancer Registry Database; National Health Insurance Research Dataset; National Register of Deaths		Tsai 2021
	National Cancer Registry; Causes of Death File; National Health Insurance Research Database		Hsieh 2020
	Longitudinal Health Insurance Database (LHID); Taiwan Cancer Registry (TCR); Cause of Death Data (CDD		Shen 2018
	Taiwanese Cancer Registry; National Health Insurance Research Dataset		Kang 2022
	Taiwan Cancer Registry (TCR); National Health Insurance Research Database; Taiwan Death registry		Chiang 2021
	Taiwan Cancer Database; National Death Certification System,; National Health Insurance Claims Database		Lin 2018
	Taiwanese National Health Insurance Research Database; National Cancer Registry		Chang 2021
	Taiwan National Cancer Registry; Taiwan National Health Insurance (NHI) data set		Lin 2018

Unique names of identified mixed clinical registries and claims databases from Taiwan (5)

Identified Clinical registry; Health insurance/claims databases from oncology disease area from Taiwan (N=64)

Database Type	Database Name	Disease Area	Study Details
**Clinical registry; Health insurance/claims**	National Health Insurance Research Database (NHIRD); Registry for Catastrophic Illness Patients Database	**Oncology (4)**	Lu 2019
	National Health Insurance Research Database (NHIRD); Registry of Catastrophic Illness Patient Database (RCIPD)		Lu 2021
	Taiwan Cancer Registry; National Health Insurance claims data; Death Registry		Tan 2018
	National Health Insurance Research Database; Taiwan Cancer Registry; National Death Registry		Hou 2022
	National Health Insurance Database; Mammography Screening database; Cancer Registry; National Mortality database		Lin 2022
	National Health Insurance (NHI) database; Taiwan Cancer Registry (TCR); Breast Cancer Screening Database		Tsai 2022
	Taiwan Cancer Registry Database (TCRD); Taiwan National Health Research Database (NHIRD); National Death Registry (NDR)		Huang 2022
	Taiwan Cancer Registry; National Health Insurance Research Database		Tang 2019
	Taiwan National Health Insurance; Taiwan Cancer Registry (TCR); Taiwan Mortality Registry		Kuo 2021
	Taiwan Cancer Registry Database (TCRD); National Health Insurance Research Database (NHIRD); National Death Registry Database (NDRD)		Huang 2018
	Taiwan Cancer Registry, National Health Insurance research database		Cheng 2021
	Taiwan Labor Insurance Databases; Taiwan Environmental Protection Agency regulatory datasets; Taiwan Cancer Registry		Fang 2021
	Taiwan Cancer Registry; National Health Insurance Database		Hsieh 2018

Unique names of identified mixed clinical registries and claims databases from Taiwan (6)

Identified Clinical registry; Health insurance/claims databases from oncology disease area from Taiwan (N=64)

Database Type	Database Name	Disease Area	Study Details
**Clinical registry; Health insurance/claims**	Taiwan Cancer Registry (TCR) database; National Health Insurance (NHI) databases; Taiwan National Death Certificate database	**Oncology (5)**	Chuang 2018
	Taiwan Cancer Registry; National Health Insurance Research Database; Death Registry		Liu 2020
	Taiwan Cancer Registry; National Health Insurance Research Database (NHIRD)		Tsai 2020
	National Health Insurance Research Database; Taiwan Cancer Registry database; National Death Registry		Huang 2020
	Taiwan Cancer Registry Database (TCR); National Health Insurance Research Database (NHIRD); National Registry of Deaths		Tsai 2018
	National Healthcare Insurance Research Database; Taiwan Death Registry		Liu 2021
	Taiwan Cancer Registry; National Mortality Registry; National Health Insurance Research Database		Chen 2019
	Taiwan Cancer Registry; National Health Insurance Research Database		Wang 2019
	Taiwan Cancer Registry Database (TCRD); National Health Insurance Research Database (NHIRD); National Death Registry		Wang 2021
	National Health Insurance Research Database; Taiwan Cancer Registry; national mortality data by the Department of Household Registration, Ministry of the Interior		Chou 2020
	Taiwan Cancer Registry; National Health Insurance Research Database		Yang 2020

Unique names of identified mixed clinical registries and claims databases from Taiwan (7)

Identified Clinical registry; Health insurance/claims databases from other disease area from Taiwan (N=18)

Database Type	Database Name	Disease Area	Study Details
**Clinical registry; Health insurance/claims**	National Health Insurance Research Database; the management information system of substitution maintenance therapy, Ministry of Health and Welfare; the road accident registry of injurious crashes, National Police Agency, Ministry of the Interior, and four independent management information systems at the Ministry of Justice	**Others**	Yang 2021
	Longitudinal health and welfare population-based database of 2010 (LHID2010); national death registry and national cancer registry		Kao 2021
	National Health Insurance Database; Cause of Death database; Multicenter Stroke Registry		Su 2021
	Registry for Catastrophic Illness Patient Database (RCIPD) linked to the Taiwan National Health Insurance Research Database (NHIRD)		Fang 2019
	National Health Insurance Research Database; Registry for Catastrophic Illness Patients		Chang 2021
	Registry for Catastrophic Illness Patients (RFCIP); Longitudinal Health Insurance Database 2000 (LHID2000), a subset of the National Health Insurance Research Database		Tsai 2020
	National Health Insurance Research Database; Registry for Catastrophic Illness Patients		Su 2021
	Taiwan Dialysis Registry Data System (TWRDS)		Chang 2022
	Taiwan's Police-Reported Traffic Accident Registry (PTAR); National Health Insurance Research Database (NHIRD		Liu 2022
	Taiwan's National Health Insurance (NHI) claims database; National Death Registry		Lin 2022
	National Health Insurance Research Database; Registry of Catastrophic Illness		Shen 2019
	National Health Insurance Research Database; Registry for Catastrophic Illness		Yang 2019
	National Health Insurance Research Database; Registry for Catastrophic Illness		Chang 2018
	National Health Insurance Research Database; National Disability Registry Database; Cause of Death File		Inchai 2021
	National Health Insurance Database; MMT program under the Centers for Disease Control and Prevention; Birth Notification System under the Bureau of Health Promotion; Birth Registration System under the Ministry of the Interior		Chen 2019
	The National Registry of Patients with Catastrophic Illness (NRPCI); the National Cause-of-Death Register (TNCDR)		Chen 2018
	National Labor Insurance Database from the Ministry of Labor; Registry of Professional Certified Chiefs from the Workforce Development Agency; National Health Insurance Research Database; National death registry; National cancer registry		Peng 2021
	Taiwanese Birth Register; National Health Insurance Research Database		You 2018

Unique names of identified EMR/EHR databases from Taiwan (1)

Identified EMR/EHR databases type from cardiology and metabolic disorders disease area from Taiwan (N=21^)

Database Type	Database Name	Disease Area	Study Details
EMR/EHR	Chang Gung Research Database	Cardiology and metabolic disorders	Fu 2021
	Chang Gung Hospital Database		Wang 2018
	Chang Gung Research Database		Tsai 2022
	Chang Gung Medical Foundation Database		Wang 2019
	Chang Gung Research Database		Chen 2021
	Chang Gung Research Database		Shao 2019
	Chang Gung Research Database		Wang 2021
	Chang Gung Research Database		Cheng 2022
	Chang Gung Research Database		Chen 2020
	Chang Gung Research Database		Shao 2020
	Chang Gung Research Database		Shao 2020
	Chang Gung Research Database		Chan 2020
	Chang Gung Research Database		Chen 2021
	Emergency medical service (EMS) database		Huang 2021
	Chang Gung Research Database		Shao 2022
	Chang Gung Research Database		Huang 2022
	Chang Gung Research Database		Shao 2019
	Chang Gung Research Database		Wang 2019

^Out of 21 studies based on EMR/EHR with cardiology and metabolic disorders, database names are available in 18 studies, while 3 studies do not mention any particular names for databases.

Unique names of identified EMR/EHR databases from Taiwan (2)

Identified EMR/EHR databases type from multiple disease areas from Taiwan (N=23^)

Database Type	Database Name	Disease Area	Study Details
EMR/EHR	Chang Gung Research Database	Infectious diseases and vaccines	Chang 2021
	E-Da Healthcare System		Hsu 2022
	Chang Gung Research Database		Chien 2022
	Taiwan Centers for Disease Control and Prevention (TWCDC) influenza surveillance and reporting system; Taipei Medical University Health Care System (TMUHcS) EMR		Yang 2019
	Cohorts from Chi Mei Medical Center; Chi Mei Liouying Hospital; Chi Mei Chiali Hospital	Inflammatory and autoimmune disorders	Chen 2021
	Chang Gung Memorial Hospital Cancer Center databank	Oncology	Liao 2020
	Chang Gung Research Database (CGRD)		Liu 2020
	Chang Gung Research Database	Others	Hsu 2021
	Taipei medical University Institutional and Clinical Database		Kao 2021
	Chang Gung Research Database (CGRD)		Tain 2021
	Chang Gung Research Database (CGRD)		Su 2022
	Chang Gung Research Database (CGRD)		Su 2021
	Hospital data from emergency department visits linked to Police Traffic Accident Dataset (PTAD)		Lin 2022
	Taiwan Adverse Drug Reaction Reporting System for Herbal Medicine		Chang 2021
	Taiwan National Adverse Drug Reaction Reporting System (Taiwan pharmacovigilance database)		Ye 2018

^Out of 23 studies based on EMR/EHR databases, names are available in 15 studies, while 2 studies do not mention any particular names for databases for oncology and 6 do not for others.

Unique names of identified mixed EMR/EHR and clinical registries databases from Taiwan (1)

Identified EMR/EHR; Clinical registry databases type from multiple disease areas from Taiwan (N=12)

Database Type	Database Name	Disease Area	Study Details
**EMR/EHR; Clinical registry**	MJ Health Database; Taiwan Cancer Registry database; Taiwan cause of death database	**Oncology**	Huang 2021
	Tzu Chi General Hospital (Dalin and Taipei) Cancer Registry; out-patients database from the Chinese Medicine and Conventional Medicine		Yeh 2020
	Taiwan Cancer Registry; Database of Kaohsiung Chang Gung Memorial Hospital (KCGMH)		Lin 2022
	National Cancer Registry; results from screening mammography performed at Kaohsiung Veterans General Hospital (VGHKS)		Pan 2018
	Taiwan Cancer Registry (TCR); single hospital chart review		Chao 2020
	cancer registry database		Shieh 2020
	2015 Mei Jau (MJ) Health Examination Database; Taiwan Cancer Registry; Causes of Death dataset		Huang 2022
	Taiwan Cancer Registry; National Birth Reporting Database; Cause-of-Death Database		Li 2020
	National TB registry database; National database of immigrant workers' physical examinations	**Infectious diseases and vaccines**	Kuan 2018
	Clinical registry of shoulder ultrasound examinations and cervical spine radiographs	**Others**	Wu 2018
	Stroke Registry of the Chang-Gung Healthcare System (SRICHS) and the Chang Gung Research Database(CGRD) from the Chang Gung Memorial Hospitals (CGMHs)		Tseng 2020
	Stroke Registry in Chang Gung Healthcare System (SRICHS); National Death Registry Database of Taiwan		Wu 2020

Unique names of identified other mixed databases from Taiwan (1)

Identified EMR/EHR; Clinical registry; Health insurance/claims (N=4) + EMR/EHR; Health insurance/claims (N=10) + EMR/EHR; Pharmacy claims (N=1) databases type from multiple disease areas from Taiwan (N=15)

Database Type	Database Name	Disease Area	Study Details
**EMR/EHR; Clinical registry; Health insurance/claims**	Longitudinal Health Insurance Database 2000 (LHID2000) of the Taiwan National Health Insurance Research Database (NHIRD); Medication records from the Department of Obstetrics and Gynecology, Chung Shan Medical University Hospital (CSMUH); cancer records from the Taiwan Cancer Registry	**Oncology**	Ciou 2022
	Birth registration system (BRS); Birth notification system (BNS); death registration system; National Health Insurance Database; national Methadone Maintenance Treatment (MMT) program	**Others**	Fang 2018
	National Health Insurance Research Database; Multiple Causes of Death Database; National Taiwan University Hospital Integrated Medical Database		Huang 2021
	Taiwan Organ Registry and Sharing Center; National Health Insurance program in Taiwan; Chang Gung Memorial Hospital at Linkou		Chan 2017
**EMR/EHR; Health insurance/claims**	National Health Insurance Research Database; Diabetes Care Management Program (DCMP); Taiwan National Death Index	**Cardiology and metabolic disorders**	Lin 2019
	National Health Insurance Research Database	**Oncology**	Yeh 2021
	National Health Insurance Research Database	**Others**	Hsu 2020
	Claims data from the National Health Insurance (NHI) program; National Registry of Deaths		Lu 2022
	Virtual private network (VPN) datasets of smoking cessation clinics by Health Promotion Administration; National Health Insurance (NHI) Administration		Wen 2022
	National Health Insurance Research Database; National Cheng Kung University Hospital (NCKUH) medical information database		Li 2019
	National Health Insurance Research Database; EHR database		Hung 2019
	National Health Insurance Research Database; Maternal and Child Health Database (MCHD); birth certificate database; death certificate database		Yang 2020
	Chang Gung Research Database; National Health Insurance Research Database		Shao 2019
	Chang Gung Research Database (CGRD); Taiwan's National Health Insurance Database (NHIRD)		Shao 2021
**EMR/EHR; Pharmacy claims**	Chang Gung Research Database	Cardiology and metabolic disorders	Chang 2020

Unique names of identified health insurance/claims databases from Taiwan (1)

Identified Health insurance/claims databases type from cardiology and metabolic disorders disease area from Taiwan (N=81)

Database Type	Database Name	Disease Area	Study Details
**Health insurance/claims**	Taiwan Diabetes Mellitus Health Database (DMHD); National Death Registry Database	**Cardiology and metabolic disorders (1)**	Wang 2022
	National Health Insurance Research Database		Wu 2019
	National Health Insurance Research Database		Chen 2020
	National Health Insurance Research Database		Lin 2018
	National Health Insurance Research Database		Wang 2018
	National Health Insurance Research Database		Kok 2018
	Longitudinal Health Insurance Database (LHID2010, LHID2005, and LHID2000) of the National Health Insurance Research Database		Huang 2020
	National Health Insurance Research Database		Wang 2022
	Longitudinal Health Insurance Database 2005 (LHID 2005), a dataset of the National Health Insurance Research Database		Yang 2018
	Longitudinal Health Insurance Database 2000 (LHID 2000), a subset of the National Health Insurance Research Database		Chang 2019
	National Health Insurance Research Database		Lin 2022
	National Health Insurance Research Database		Wang 2022
	National Health Insurance Research Database		Wu 2021
	National Health Insurance Research Database		Chou 2021
	National Health Insurance Research Database		Yang 2020
	National Health Insurance Research Database		Dong 2020
	National Health Insurance Research Database		Su 2019
	National Health Insurance Research Database		Yeh 2021

Unique names of identified health insurance/claims databases from Taiwan (2)

Identified Health insurance/claims databases type from cardiology and metabolic disorders disease area from Taiwan (N=81)

Database Type	Database Name	Disease Area	Study Details
Health insurance/claims	2003 National Health Insurance claims database for diabetes	Cardiology and metabolic disorders (2)	Chiou 2021
	Longitudinal Health Insurance Database for catastrophic illness patients (LHID-CIP)		Lu 2019
	Taiwan National Health Insurance claims database		Lai 2018
	National Health Insurance Research Database		Yang 2018
	Longitudinal Cohort of Diabetes Patients		Cheng 2018
	Taiwan National Health Insurance claims data		Chou 2021
	National Health Insurance Research Database		Lin 2018
	National Health Insurance Research Database		Lai 2018
	National Health Insurance Research Database		Wu 2021
	National Health Insurance Research Database		Lin 2019
	National Health Insurance Research Database		Ho 2018
	National Health Insurance Research Database		Liang 2018
	National Health Insurance Research Database		Tseng 2020
	Longitudinal Health Insurance Database 2000 (LHID2000) of the National Health Insurance Research Database		Hsieh 2019
	Longitudinal Health Insurance Database 2005		Liu 2020
	National Health Insurance Research Database		Chan 2020
	National Health Insurance Research Database		Porta 2019
	Longitudinal Cohort of Diabetes Patients (LHDB) from Taiwan’s NHI programme		Chen 2018
	Longitudinal Health Insurance Database (LHID)		Hung 2019
	National Health Insurance Research Database		Tai 2021

Unique names of identified health insurance/claims databases from Taiwan (3)

Identified Health insurance/claims databases type from cardiology and metabolic disorders disease area from Taiwan (N=81)

Database Type	Database Name	Disease Area	Study Details
**Health insurance/claims**	National Health Insurance Research Database	**Cardiology and metabolic disorders (3)**	Tung 2017
	National Health Insurance Research Database; Longitudinal Health Insurance Database 2000 (LHID2000)		Huang 2019
	National Health Insurance Research Database		Weng 2018
	Longitudinal Cohort of Diabetes Patients (LHDP) claims dataset		Chen 2017
	National Health Insurance Research Database		Liu 2017
	Longitudinal Cohort of Diabetes Patients (LHDB), a sub-data set of the National Health Insurance Research Database		Huang 2019
	Longitudinal Health Insurance Database (LHID2000) from Taiwan National Health Insurance program		Chou 2018
	National Health Insurance Research Database		Huang 2017
	Longitudinal Health Insurance Dataset (LHID) provided by the Taiwan National Health Research Institutes (NHRI)		Hu 2018
	National Health Insurance Research Database		Lin 2019
	Longitudinal Health Insurance Database 2000 (LHID2000), a subset data of the National Health Insurance Research Institute Database		Wang 2021
	National Health Insurance Research Database (NHIRD)		Yeh 2019
	Year 2000 version of the Longitudinal Health Insurance Database (LHID)		Lee 2018
	Longitudinal Health Insurance Database 2000 (LHID2000) a subset of the National Health Insurance Research Database		Tsai 2019
	Taiwan National Health Insurance Research Database		Chao 2018
	National Health Insurance Research Database		Kuo 2018
	National Health Insurance Research Database		Lin 2019
	National Health Insurance Research Database		Lin 2019
	National Health Insurance Research Database		Huang 2019
	Bureau of National Health Insurance (BNHI) research database		Chen 2017
	Longitudinal Health Insurance Database 2000 (LHID2000) a subset of the National Health Insurance Research Database		Hsieh 2018
	National Health Insurance Research Database		Lee 2021

Unique names of identified health insurance/claims databases from Taiwan (4)

Identified Health insurance/claims databases type from cardiology and metabolic disorders disease areas from Taiwan (N=81)

Database Type	Database Name	Disease Area	Study Details
**Health insurance/claims**	National Health Insurance Research Database	**Cardiology and metabolic disorders (4)**	Chao 2018
	National Health Insurance Research Database		Ma 2018
	Longitudinal Health Insurance Database 2000 (LHID2000) from the Taiwan National Health Insurance (NHI) program		Hu 2018
	National Health Insurance Research Database		Chen 2019
	National Health Insurance Research Database		Li 2019
	National Health Insurance Research Database		Lu 2021
	National Health Insurance claims Database		Lin 2018
	National Health Insurance claims database		Chen 2019
	National-Health-Insurance claims		Lin 2019
	National Health Insurance Research Database		Sung 2021
	National Health Insurance Research Database		Lu 2019
	National Health Insurance Research Database		Chen 2018
	National Health Insurance Research Database		Lee 2018
	National Health Insurance Research Database		Ding 2018
	2005 Longitudinal Health Insurance Database (LHID2005) of the National Health Insurance Research Database		Cheng 2019
	Taiwanese National Health Care Claim database (National Health Insurance Administration 2014)		Chen 2019
	National Health Insurance Database		Wang 2021
	Health and Welfare Data Science Center (HWDC) database, Ministry of Health and Welfare, Taiwan.		Wu 2021
	National Health Insurance Database		Lin 2019
	National Health Insurance Research Database; Taiwan Death Registry; validation cohort at Linkou Chang Gung Memorial Hospital		Chen 2020
	National Health Insurance Research Database		Chou 2020

Unique names of identified health insurance/claims databases from Taiwan (5)

Identified Health insurance/claims databases type from infectious diseases and vaccines disease area from Taiwan (N=13)

Database Type	Database Name	Disease Area	Study Details
**Health insurance/claims**	Administrative claims-based database released by the Health and Welfare Data Science Center, Ministry of Health and Welfare, Taiwan	**Infectious diseases and vaccines**	Fu 2022
	National Health Insurance Research Database; Taiwan Environmental Protection Administration (EPA)		Tseng 2021
	Longitudinal Health Insurance Database 2005 (LHID2005), a subset of the National Health Insurance Research Database		Tung 2018
	Ambulatory claims database from the National Health Insurance Research Database		Lin 2022
	National Health Insurance (NHI); Longitudinal Health Insurance Database		Lin 2019
	Longitudinal Health Insurance Database 2005		Chen 2018
	Longitudinal Health Insurance Database 2005 (LHID 2005) a subset of the National Health Insurance Research Database		Tsao 2020
	Longitudinal Health Insurance Database 2000 (LHID2000) a subset of the National Health Insurance Research Database		Chang 2021
	Health and Welfare Database		Wang 2022
	National Health Insurance Research Database		Chen 2021
	Longitudinal Health Insurance Database 2000 (LHID 2000), a subset database of the National Health Insurance Research Database		Wu 2018
	National Health Insurance Research Database (NHIRD)		Tsai 2019
	Taiwan National Health Insurance Research Database		Teh 2020

Unique names of identified health insurance/claims databases from Taiwan (6)

Identified Health insurance/claims databases type from inflammatory and autoimmune disorders disease area from Taiwan (N=54)

Database Type	Database Name	Disease Area	Study Details
Health insurance/claims	National Health Insurance Database (NHID)	Inflammatory and autoimmune disorders (1)	Yang 2018
	Inpatient database of the Bureau of National Health Insurance; National Health Insurance Research Database		Chang 2020
	Longitudinal Health Insurance Database (LHID) a sub-dataset of the National Health Insurance (NHI) program		Chung 2020
	Longitudinal Health Insurance Database (LHID2000) a subset of the National Health Insurance Research Database		Wu 2018
	Longitudinal Health Insurance Database 2000		Koh 2020
	Longitudinal Health Insurance Database 2000		Lai 2019
	Longitudinal Health Insurance Database 2000 (LHID 2000) a subset of the National Health Insurance Research Database		Lu 2018
	Longitudinal Health Insurance Database 2000 (LHID 2000) of the National Health Insurance Research Database		Lu 2017
	Longitudinal Health Insurance Database 2000 (LHID 2000), a subset of the National Health Insurance Research Database		Jan 2021
	Longitudinal Health Insurance Database 2000 (LHID2000)		Liu 2019
	Longitudinal Health Insurance Database 2000 (LHID2000) a subset of the National Health Insurance Research Database		Sun 2020
	Longitudinal Health Insurance Database 2000 (LHID2000) and 2010 (LHID2010) of the National Health Insurance Research Database; Registry for Catastrophic Illness Patient Database		Lin 2021
	Longitudinal Health Insurance Database 2000 (LHID2000) of the National Health Insurance Research Database		Chang 2021
	Longitudinal Health Insurance Database 2000 (LHID2000) of the National Health Insurance Research Database		Lien 2020
	Longitudinal Health Insurance Database 2000 (LHID2000) of the National Health Insurance Research Database		Lu 2019
	Longitudinal Health Insurance Database 2000 (LHID2000) of the National Health Insurance Research Database		Liu 2018
	Longitudinal Health Insurance Database 2000, (LHID200) a subset of the National Health Insurance Research Database		Li 2022
	Longitudinal Health Insurance Database 2005 (LHID2005), a sub-database of National Health Insurance Research Database		Chen 2020
	Longitudinal Health Insurance Database 2010		Lee 2018
	Longitudinal Health Insurance Database 2010 (LHID2010) a subset of the National Health Insurance Research Database		Li 2018
	Longitudinal Health Insurance Database 2010 (LHID2010) of the National Health Insurance Research Database		Chan 2019
	Longitudinal Health Insurance Database of 1 million enrollees in the Taiwan National Health Insurance (NHI) program between 2000 and 2011		Yeh 2019
	Longitudinal Health Insurance Dataset (LHID) of the National Health Insurance Research Database		Kok 2018
	National Health Informatics Project		Yu 2019
	National Health Insurance (NHI) claims data		Shantakumar 2018
	National health insurance (NHI) database; national death certificate registry database		Yo 2018
	National Health Insurance claims database of Taiwan		Lee 2017
	National Health Insurance Database (NHID)		Yang 2018

Unique names of identified health insurance/claims databases from Taiwan (7)

Identified Health insurance/claims databases type from inflammatory and autoimmune disorders disease area from Taiwan (N=54)

Database Type	Database Name	Disease Area	Study Details
**Health insurance/claims**	National Health Insurance Research Database	**Inflammatory and autoimmune disorders (2)**	Chen 2018
	National Health Insurance Research Database		Chiang 2021
	National Health Insurance Research Database		Chuang 2018
	National Health Insurance Research Database		Hsieh 2020
	National Health Insurance Research Database		Hsieh 2021
	National Health Insurance Research Database		Hsu 2020
	National Health Insurance Research Database		Lin 2018
	National Health Insurance Research Database		Lin 2021
	National Health Insurance Research Database		Tang 2022
	National Health Insurance Research Database		Tung 2021
	National Health Insurance Research Database		Weng 2021
	National Health Insurance Research Database - 2000-2012 catastrophic illness data file; 2000 Longitudinal Health Insurance Database (LHID2000)		Chuang 2020
	National Health Insurance Research Database (NHIRD)		Nien 2018
	National Health Insurance Research Database (NHIRD)		Yu 2017
	National Health Insurance Research Database (NHIRD); 2000 Longitudinal Health Insurance Database		Li 2019
	National Health Insurance Research Database (NHIRD); 2000 Longitudinal Health Insurance Database (LHID 2000)		Hsu 2020
	National Health Insurance Research Database, Ministry of Health and Welfare (NHIRD-MOHW)		Chou 2022
	National Health Insurance Research Database; Longitudinal Health Insurance Database 2000 (LHID2000)		Weng 2021
	National Health Insurance Research Database; Longitudinal Health Insurance Database		Hsu 2021
	National Health Insurance Research Database; Longitudinal Health Insurance Database 2000		Weng 2019
	National Health Insurance Research Database; Longitudinal Health Insurance Database 2000 (LHID2000), a data subset of the National Health Insurance Research Database		Wu 2018
	Registry for Catastrophic Illness Patient Database (CIPD); Longitudinal Health Insurance Database (LHID2000)		Huang 2018
	Taiwan National Health Insurance Research Database		Chang 2019

Unique names of identified health insurance/claims databases from Taiwan (8)

Identified Health insurance/claims databases type from oncology disease area from Taiwan (N=29)

Database Type	Database Name	Disease Area	Study Details
**Health insurance/claims**	National Insurance Research Database (NHIRD); Longitudinal Health Insurance Database	**Oncology (1)**	Lu 2021
	National Health Insurance Research Database		Kok 2019
	Population-based administrative datasets from the Health and Welfare Data Science Center (HWDC) and from The National Health Informatics Project (NHIP)		Hsiao 2021
	National Health Insurance Research Database		Wang 2021
	Taiwan Cancer Registry Database; National Health Insurance Database; Death File Database		MachucaMPG 2022
	Longitudinal Health Insurance Database 2000; Registry of Catastrophic Illness Patient Database		Ding 2019
	Longitudinal Health Insurance Database 2000 (LHID 2000) of the National Health Insurance Research Database (NHIRD)		Li 2020
	National Health Insurance Research Database (NHIRD)		Liu 2019
	National Health Insurance Research Database (NHIRD) of the Taiwan Bureau of National Health Insurance (BNHI)		Wu 2021
	National Health Insurance Research Database		Chen 2018
	National Health Insurance Research Database		Chen 2018
	National Health Insurance Research Database		Kuo 2019
	Longitudinal Health Insurance Database 2005		Lee 2018
	Health Promotion Administration, Ministry of Health and Welfare, Taiwan		Lin 2018
	National Health Insurance Research Database		Wu 2018
	National Health Insurance Research Database		Pan 2020
	Longitudinal Health Insurance Database 2005 (LHID2005), a subset of the National Health Insurance Research Database		Wu 2018

Unique names of identified health insurance/claims databases from Taiwan (9)

Identified Health insurance/claims databases type from oncology disease area from Taiwan (N=29)

Database Type	Database Name	Disease Area	Study Details
**Health insurance/claims**	National Health Insurance Research Database (NHIRD)	**Oncology (2)**	Huang 2021
	Taiwan National Health Insurance Research Database		Chen 2022
	National Health Insurance Research Database		Tsao 2018
	Registry for Catastrophic Illness Patient Database (RCIPD), a subset of the National Health Insurance Research Database (NHIRD)		Tseng 2020
	National Health Insurance Research Database		Yang 2021
	Taiwanese Registry for Catastrophic Illness Patients Database; National Health Insurance Research Database		Wang 2021
	National Health Insurance Research Database		Yeh 2020
	National Health Insurance Research Database		Tsai 2019
	National Health Insurance Research Database; Longitudinal Health Insurance Database (LHID2010)		Phan 2020
	Immune Checkpoint Inhibitor Registry Database (ICIRD) of the National Health Insurance Administration; National Health Insurance Research Database (NHIRD)		Hsieh 2021
	National Health Insurance Database; Registry for Catastrophic Illness		Padua 2021
	National Health Insurance Research Database		Liao 2020

Unique names of identified health insurance/claims databases from Taiwan (10)

Identified Health insurance/claims databases type from 'others' disease area from Taiwan (N=166)

Database Type	Database Name	Disease Area	Study Details
**Health insurance/claims**	Longitudinal Health Insurance Database (LHID) ; National Health Insurance Research Database (NHIRD)	**Others (1)**	Lee 2021
	National Health Insurance Research Database		Pan 2018
	Taiwan National Health Insurance Research Database (NHIRD)		Hsu 2022
	(1) Taiwan Cancer Registry (TCR), (2) NHI claims repository, (3) National Death Registry, and (4) Registry for Board-certified Specialists and Hospitals.		Chao 2021
	2000, 2005, and 2010 cohorts of the Longitudinal Health Insurance Databases (LHID) of the National Health Insurance Research Database		Lin 2018
	2005 Longitudinal Health Insurance Database (LHID2005), a subset of the National Health Insurance Research Database (NHIRD)		Kuo 2018
	3 Longitudinal Health Insurance Databases (LHID2000, LHID2005, and LHID2010) of the National Health Insurance Research Database		Liao 2021
	Bureau of National Health Insurance		Chang 2019
	Bureau of National Health Insurance (BNHI) Taiwan		Chiou 2020
	Claims data of Taiwan's National Health Insurance		Tai 2019
	database from the National Health Research Institutes		Tseng 2020
	Database of the Taiwan National Health Insurance Program		Lin 2018
	Household Registration Database; National Health Insurance Database; National Registration of Deaths		Iqbal 2019
	Longitudinal Health Insurance Database 2000		Lin 2020
	Longitudinal Health Insurance Database 2000 (LHID 2000) a subset of the National Health Insurance Research Database		Hung 2022
	Longitudinal Health Insurance Database 2000 (LHID2000) a subset of the National Health Insurance Research Database		Hou 2018

Unique names of identified health insurance/claims databases from Taiwan (11)

Identified Health insurance/claims databases type from ‘others’ disease area from Taiwan (N=166)

Database Type	Database Name	Disease Area	Study Details
Health insurance/claims	Longitudinal generation tracking database (2000-2016) of the Taiwan National Health Insurance Database	Others (2)	Yu 2021
	Longitudinal Generation Tracking Database (LGTD) of the Taiwan National Health Insurance claims database; National Death Certification Registry		Wang 2020
	Longitudinal Health Insurance Data-base 2005 (LHID2005), derived from Taiwan’s National Health Insurance (NHI) program		Ho 2018
	Longitudinal Health Insurance Database (2000-2013) of the National Health Insurance Research Database		Feng 2021
	Longitudinal Health Insurance Database (LHID) 2000 of the Taiwan National Health Research Institutes		Lin 2021
	Longitudinal Health Insurance Database (LHID) of all enrollees covered under the Taiwan's National Health Insurance program in 2005		Lai 2018
	Longitudinal Health Insurance Database (LHID) of all the national health insurance (NHI) enrollees		Tsai 2017
	Longitudinal Health Insurance Database (LHID) within the National Health Insurance Research Database		Tsai 2021
	Longitudinal Health Insurance Database (LHID2005) of the National Health Insurance Research Database		Lin 2020
	Longitudinal Health Insurance Database 2000		Hsieh 2018
	Longitudinal Health Insurance Database 2000 (LHID2000) of the National Health Insurance Research Database (NHIRD)		Hsia 2018
	Longitudinal Health Insurance Database 2000 (LHID2000)		Lin 2018
	Longitudinal Health Insurance Database 2000 (LHID2000), a subset of the Taiwan National Research Health Insurance Database		Lin 2019
	Longitudinal Health Insurance Database 2000 (LHID 2000), a sub-dataset of the National Health Insurance Research Dataset		Yu 2018
	Longitudinal Health Insurance Database 2000 (LHID2000) a subset of the National Health Insurance Research Database		Tang 2021
	Longitudinal Health Insurance Database 2000 (LHID2000) a subset of the National Health Insurance Research Database		Yang 2017

Unique names of identified health insurance/claims databases from Taiwan (12)

Identified Health insurance/claims databases type from ‘others’ disease area from Taiwan (N=166)

Database Type	Database Name	Disease Area	Study Details
**Health insurance/claims**	Longitudinal Health Insurance Database 2000 (LHID2000) of the National Health Insurance Research Database	**Others (3)**	Chen 2017
	Longitudinal Health Insurance Database 2000 (LHID2000) of the National Health Insurance Research Database		Lee 2021
	Longitudinal Health Insurance Database 2000 (LHID2000) of the National Health Insurance Research Database		Liu 2021
	Longitudinal Health Insurance Database 2000 (LHID2000) of the National Health Insurance Research Database		Tsai 2020
	Longitudinal Health Insurance Database 2000 (LHID2000) within NHIRD; Environment Resource Dataset from Environmental Protection Administration (EPA) of Taiwan		Zhang 2019
	Longitudinal Health Insurance Database 2000 (LHID2000), a subset of Taiwan's National Health Insurance Research Database		Chang 2021
	Longitudinal Health Insurance Database 2000 (LHID2000), a subset of the National Health Insurance Research Database		Hung 2021
	Longitudinal Health Insurance Database 2000 (LHID2000), a subset of the National Health Insurance Research Database		Yang 2019
	Longitudinal Health Insurance Database 2000; National Health Insurance Research Database		Lin 2018
	Longitudinal Health Insurance Database 2005		Chiu 2018
	Longitudinal Health Insurance Database 2005 (LHID2005)		Chu 2017
	Longitudinal Health Insurance Database 2005 (LHID2005) of the National Health Insurance Research Database		Lo 2019
	Longitudinal Health Insurance Database 2005, a subset of the National Health Insurance Research Database		Hwang 2018
	Longitudinal Health Insurance Database 2010 (LHID 2010) of the National Health Insurance Research Database		Hsu 2020
	Longitudinal Health Insurance Database 2010 (LHID 2010) of the National Health Insurance Research Database; Registry for Catastrophic Illnesses,		Wang 2020
	Longitudinal Health Insurance Database of Taiwan		Kuo 2021
	Longitudinal Health Insurance Database of the National Health Insurance Research Database during the period of 2000-2013		Wei 2021
	Longitudinal Health Insurance Database, a subset of the National Health Insurance Research Database		Hsieh 2020
	Longitudinal Health Insurance Database(LHID2010)		Huang 2019

Unique names of identified health insurance/claims databases from Taiwan (13)

Identified Health insurance/claims databases type from ‘others’ disease area from Taiwan (N=166)

Database Type	Database Name	Disease Area	Study Details
**Health insurance/claims**	Longitudinal Health Insurance Databases (LHID2000, LHID2005, and LHID2010) from the original National Health Insurance research Database	**Others (4)**	Wu 2020
	Longitudinal Health Insurance Databases 2000 (LHID2000), 2005 (LHID2005), and 2010 (LHID2010) of the National Health Insurance Research Database		Lee 2017
	Longitudinal Health Insurance Dataset 2005 of the National Health Insurance Research Database		Kok 2018
	Nation Health Insurance Research Database		Hu 2022
	National Health Insurance Administration claims database		Chen 2020
	National Health Insurance Research Database		Kang 2019
	National Health Insurance		Lin 2018
	National Health Insurance (NHI) claims data; four clinical trials: BEACON, SHINE, SPARK, and ILLUMINATE		Chan 2018
	National Health Insurance claims dataset		Lin 2020
	National Health Insurance Database		Ho 2022
	National Health Insurance Database		Hsu 2022
	National Health Insurance Research and Development (NHIRD) database		Islam 2020
	National Health Insurance Research Database		Chang 2020
	National Health Insurance Research Database		Chang 2020
	National Health Insurance Research Database		Chao 2022
	National Health Insurance Research Database		Chen 2019
	National Health Insurance Research Database		Chen 2017

Unique names of identified health insurance/claims databases from Taiwan (14)

Identified Health insurance/claims databases type from ‘others’ disease area from Taiwan (N=166)

Database Type	Database Name	Disease Area	Study Details
**Health insurance/claims**	National Health Insurance Research Database	**Others (5)**	Chen 2019
	National Health Insurance Research Database		Chen 2019
	National Health Insurance Research Database		Chen 2019
	National Health Insurance Research Database		Chen 2021
	National Health Insurance Research Database		Chen 2022
	National Health Insurance Research Database		Chen 2022
	National Health Insurance Research Database		Cheng 2018
	National Health Insurance Research Database		Chou 2020
	National Health Insurance Research Database		Chou 2021
	National Health Insurance Research Database		Chuang 2018
	National Health Insurance Research Database		Chuang 2018
	National Health Insurance Research Database		Hsieh 2022
	National Health Insurance Research Database		Hsu 2019
	National Health Insurance Research Database		Jeng 2018
	National Health Insurance Research Database		Huang 2019
	National Health Insurance Research Database		Huang 2019
	National Health Insurance Research Database		Huang 2021

Unique names of identified health insurance/claims databases from Taiwan (15)

Identified Health insurance/claims databases type from 'others' disease area from Taiwan (N=166)

Database Type	Database Name	Disease Area	Study Details
**Health insurance/claims**	National Health Insurance Research Database	**Others (6)**	Kaewboonchoo 2019
	National Health Insurance Research Database		Kao 2021
	National Health Insurance Research Database		Kuang 2020
	National Health Insurance Research Database		Kuo 2019
	National Health Insurance Research Database		Lai 2018
	National Health Insurance Research Database		Lam 2019
	National Health Insurance Research Database		Lee 2020
	National Health Insurance Research Database		Li 2018
	National Health Insurance Research Database		Li 2021
	National Health Insurance Research Database		Liang 2020
	National Health Insurance Research Database		Liang 2022
	National Health Insurance Research Database		Liao 2018
	National Health Insurance Research Database		Liao 2020
	National Health Insurance Research Database		Lin 2017
	National Health Insurance Research Database		Lin 2017
	National Health Insurance Research Database		Lin 2018
	National Health Insurance Research Database		Lin 2018

Unique names of identified health insurance/claims databases from Taiwan (16)

Identified Health insurance/claims databases type from ‘others’ disease area from Taiwan (N=166)

Database Type	Database Name	Disease Area	Study Details
**Health insurance/claims**	National Health Insurance Research Database	**Others (7)**	Lin 2019
	National Health Insurance Research Database		Lin 2021
	National Health Insurance Research Database		Lin 2021
	National Health Insurance Research Database		Liou 2018
	National Health Insurance Research Database		Liu 2019
	National Health Insurance Research Database		Sun 2019
	National Health Insurance Research Database		Sung 2018
	National Health Insurance Research Database		Tsai 2017
	National Health Insurance Research Database		Tsai 2019
	National Health Insurance Research Database		Tsai 2022
	National Health Insurance Research Database		Tsai 2022
	National Health Insurance Research Database		Wang 2019
	National Health Insurance Research Database		Wang 2021
	National Health Insurance Research Database		Wu 2021
	National Health Insurance Research Database		Yu 2018
	National Health Insurance Research Database		Yang 2020
	National Health Insurance Research Database		Yang 2022

Unique names of identified health insurance/claims databases from Taiwan (17)

Identified Health insurance/claims databases type from ‘others’ disease area from Taiwan (N=166)

Database Type	Database Name	Disease Area	Study Details
**Health insurance/claims**	National Health Insurance Research Database	**Others (8)**	Cheng 2017
	National Health Insurance Research Database		Chiang 2019
	National Health Insurance Research Database		Kuang 2022
	National Health Insurance Research Database		Lai 2022
	National Health Insurance Research Database		Lee 2022
	National Health Insurance Research Database		Li 2018
	National Health Insurance Research Database		Lu 2020
	National Health Insurance Research Database		Tang 2021
	National Health Insurance Research Database		Wang 2018
	National Health Insurance Research Database		Yeh 2020
	National Health Insurance Research Database (NHIRD)		Dovgan 2020
	National Health Insurance Research Database (NHIRD)		Lin 2018
	National Health Insurance Research Database (NHIRD)		Lin 2019
	National Health Insurance Research Database, Longitudinal Health Insurance Database (LHID) 2005 and 2010 subsets.		Hsu 2021
	National Health Insurance Research Database; Longitudinal Cohort of Diabetes Patients		Chen 2019
	National Health Insurance Research Database; Death Registry		Tang 2021

Unique names of identified health insurance/claims databases from Taiwan (18)

Identified Health insurance/claims databases type from ‘other’ disease area from Taiwan (N=166)

Database Type	Database Name	Disease Area	Study Details
**Health insurance/claims**	National Health Insurance Research Database; Longitudinal Health Insurance Database (2000)	**Others (9)**	Ou 2022
	National Health Insurance Research Database; Longitudinal Health Insurance Database 2000		Jeng 2018
	National Health Insurance Research Database; Registry for Catastrophic Illness Patient Database		Yen 2018
	National Health Insurance Research Database; Taiwan Longitudinal Health Insurance Database 2000		Jan 2017
	National Health Interview Survey (NHIS) 2001, 2005, and 2009; National Health Insurance (NHI) claims data; National Register of Deaths Database		Lai 2017
	National Health Research Institute databank		Sung 2018
	National Taiwan Health Insurance		Wang 2018
	Nationwide COPD P4P enrollment program, Taiwan National Health Insurance claims database		Cheng 2021
	Nationwide Poisoning Database (NPD); Longitudinal Health Insurance Database 2000 (LHID2000)		Huang 2018
	Registry for Catastrophic Illness Patients from the National Health Insurance Research Database		Wang 2020
	Registry of Catastrophic Illness Database (subset of NHI Research Database)		Fan 2019
	Reimbursement claims of Taiwan’s National Health Insurance		Huang 2021
	Taipei Medical University Clinical Research Database (TMUCRD		HoangAnh 2022
	Taiwan Longitudinal Health Insurance Database 2000 (LHID2000)		Lin 2019
	Taiwan National Health Insurance Research Database; Longitudinal Health Insurance Database 2000 (LHID 2000)		Chang 2020
	Taiwan National Health Insurance (LHID2005: Longitudinal Health Insurance Database 2005)		Chiou 2022

Unique names of identified health insurance/claims databases from Taiwan (19)

Identified Health insurance/claims databases type from ‘others’ disease area from Taiwan (N=166)

Database Type	Database Name	Disease Area	Study Details
**Health insurance/claims**	Taiwan National Health Insurance (NHI) claims database	**Others (10)**	Weng 2020
	Taiwan National Health Insurance Administration (NHIA)		Shih 2020
	Taiwan National Health Insurance claims database		Yan 2017
	Taiwan National Health Insurance Database		Lin 2021
	Taiwan National Health Insurance Database		Yu 2019
	Taiwan National Health Insurance Research Database		Chen 2018
	Taiwan National Health Insurance Research Database		Hung 2021
	Taiwan National Health Insurance Research Database		Wu 2021
	Taiwan National Health Insurance Research Database (NHIRD)		Wang 2019
	Taiwan National Health Insurance Research Database; Longitudinal Health Insurance Database 2000 (LHID2000)		Chang 2019
	Taiwan National Health Insurance Research Dataset		Cheng 2020
	Taiwan NationalHealth Insurance Research Database (NHIRD), Longitudinal Health Insurance Database 2000 (LHID2000)		Chang 2020
	Taiwan Renal Registry Data System (TWRDS)		Kuo 2022
	Taiwan's 2005 Longitudinal Generation Tracking Database (LGTD2005)		Chen 2022
	Taiwan's National Health Insurance program		Huang 2020

Unique names of identified mixed Health insurance/claims and pharmacy clinical registries databases from Taiwan (1)

Identified Health insurance/claims; Pharmacy claims databases type from ‘other’ disease area from Taiwan (N=1)

Database Type	Database Name	Disease Area	Study Details
**Health insurance/claims; Pharmacy claims**	Taiwan National Health Insurance Research Database (NHIRD) .	**Other**	Liao 2021

Only one study (Liao 2021) from all eligible studies across all countries included *pharmacy claims* database

Liao et al. studied the prescription Patterns in Patients with Chronic Obstructive Pulmonary Disease and Osteoporosis. Data consisted of all medical claims, pharmacy claims and causes of death from National Health Insurance Database.

TOPLINE RESULTS

Real-world studies originating from contemporary integrated databases

Databases identified names from India

Scoping review for Taiwan, India, and Thailand

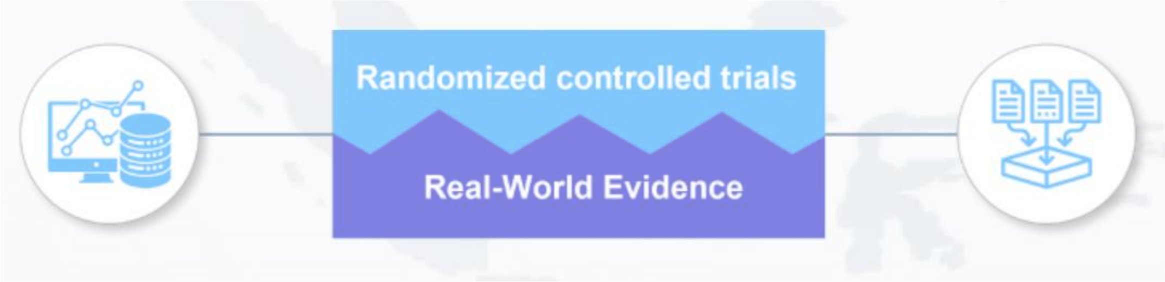

Unique names of identified clinical registries databases from India (1)

Identified registry databases from cardiology and metabolic disorders disease area from India (N=19^)

Database Type	Database Name	Disease Area	Study Details
**Clinical registry**	Envision en-ABL-e registry	**Cardiology and metabolic disorders**	Testa 2018
	Envision en-ABL-e registry		Sharma 2021
	HP-India ACS Registry		Mahajan 2017
	Indian College of Cardiology National Heart Failure Registry		Jayagopal 2021
	Indian Phenotype Registry		Kalra 2022
	Indian PHPT registry		Bhadada 2018
	KERALA-AF Registry		Bahuleyan 2021
	Nanoluté Registry		El-Mokdad 2020
	Nanoluté Registry		Dani 2019
	PERFORMance of biodegradable polymer-coated ultra-thin EVERolimus-eluting stents (PERFORM EVER)		Kasturi 2022
	Primary Angioplasty Registry of Kerala		Jabir 2017
	Primary percutaneous coronary intervention (PPCI) registry of Kerala		Jabir 2020
	South Asian Systolic Heart Failure Registry (SASHFR)		Naik 2018
	T-Flex registry		Pothineni 2021
	Tetriflex SES registry		Ajmera 2019
	The Prospective Observational Longitudinal Registry of patients with stable coronary artery disease (CLARIFY) registry		Hiremath 2018
	Trivandrum Heart Failure Registry (THFR)		Sanjay 2018

^Out of 19 studies based on clinical registries with cardiology and metabolic disorders, databases name are available in 17 studies while 2 studies do not mention any particular names for databases.

Unique names of identified clinical registries databases from India (2)

Identified registry databases from other disease areas from India (N=16^)

Database Type	Database Name	Disease Area	Study Details
**Clinical Registry**	Health Maternal and Newborn Health Registry	**Infectious diseases and vaccines**	Arlington 2019
	PregCovid registry in Maharashtra, India		Malik 2022
	Adalimumab Biosimilar Patient Registry (ASPIRE)	**Inflammatory and autoimmune disorders**	Kapoor 2019
	Adalimumab Biosimilar Patient Registry (ASPIRE)		Kapoor 2019
	Association of Surgical Gastroenterologists of Kerala (ASGK) CRC registry	**Oncology**	Krishnan 2020
	Hyperthermic Intraperitoneal Chemotherapy (HIPEC) Registry		Bhatt 2018
	India State-Level Disease Burden Initiative, Nationwide Sample Registration System		Global Burden of Disease Study 1990-2016
	OncoCollect Lymphoma Registry		Nair 2021
	Abdominal Wall Reconstruction Surgical Collaborative registry	**Others**	Arora 2022
	Australia-India Trauma Systems Collaboration Project		Banerjee 2022
	Global Observatory on Donation and Transplantation questionnaire		Ramesh 2021
	Nagpur, India site of the Global Network for Women and Children Health Research Maternal and Neonatal Health Registry		Simmons 2021
	The Indian PCRRT-ICONIC Neonatal Kidney Educational Registry (TINKER)		Sethi 2022
	Towards Improved Trauma Care Outcomes in India (TITCO) registry		Sinha 2020

^Out of 16 studies based on clinical registries infectious diseases and vaccines (n=3), inflammatory and autoimmune disorders (n=2), oncology (n=5), and others (n=6), databases name are available in 14 studies while 2 studies do not mention any particular names for databases [Infectious diseases and vaccines (1) & oncology (1).

Unique names of other databases from India

Identified other database type from multiple disease areas from India (N=46^)

Database Type	Database Name	Disease Area	Study Details
EMR/EHR	HealthPlix EMR	Cardiology and metabolic disorders	Khan 2021
	HealthPlix EMR		Khan 2021
	HealthPlix EMR		Polavarapu 2020
	EyeSmart EMR	Infectious diseases and vaccines	Rathi 2020
	EyeSmart EMR		Das 2022
	Pooled electronic and physical medical records of 7 tertiary eye care centers		Das 2022
	Eye Smart EMR	Inflammatory and autoimmune disorders	Tyagi 2022
	Australia-India Trauma Systems Collaboration (AITSC) registry	Others	Sarang 2021
	EyeSmart EMR		Thakur 2021
	EyeSmart EMR		Behera 2021
	HealthPlix EMR		Bansal 2022
	India Ozurdex Postmarketing Surveillance Study, observational, prospective, non-interventional, post-marketing surveillance program		Nair 2020
	EyeSmart EMR		Das 2019
	EyeSmart EMR		Das 2020
	OpenClinica		Bassi 2019
	Towards Improved Trauma Care Outcomes in India (TITCO) registry		Bansal 2022
EMR/EHR; Clinical registry	PINNACLE (Practice Innovation and Clinical Excellence) India Quality Improvement Program (PIQIP)	Cardiology and metabolic disorders	Kalra 2018
	OncoCollect Lymphoma Registry	Oncology	Bhurani 2021
Health insurance/claims	Arogyasri health insurance scheme	Others	Singh 2022

^Out of 46 studies based on EMR/EHR (n=42), EMR/HER; clinical registry (n=3), and health insurance/claims (n=1), databases names are available in 19 studies while 27 studies do not mention any particular names for databases [EMR/EHR: cardiology and metabolic (12), infectious diseases and vaccines (3), inflammatory and autoimmune disorders (2), oncology (2), others (7) - EMR/EHR; Clinical Registry (1)]

Abbreviations: EHR, or electronic health records; EMR, electronic medical records

TOPLINE RESULTS

Real-world studies originating from contemporary integrated databases

Databases identified names from Thailand

Scoping review for Taiwan, India, and Thailand

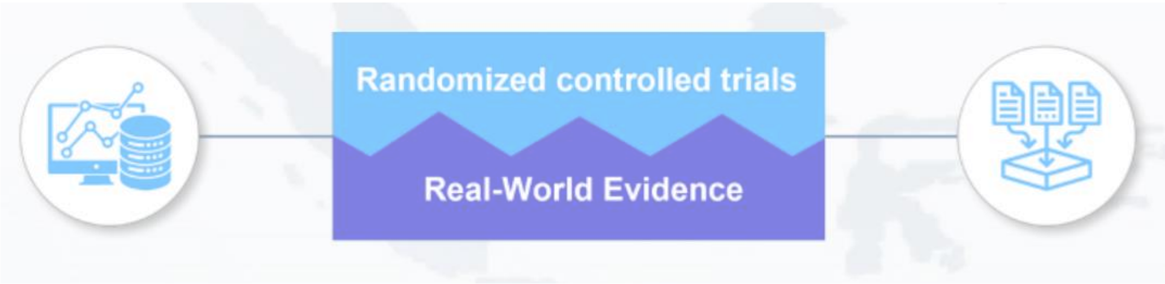

Unique names of identified clinical registries databases from Thailand (1)

Identified registry databases from cardiology and metabolic disorders & inflammatory and autoimmune disorders disease area from Thailand (N=9^)

Database Type	Database Name	Disease Area	Study Details
**Clinical registry**	A nationwide multicenter registry of patients with NVAF	**Cardiology and metabolic disorders**	Krittayaphong 2018
	Cardiac Intervention Association of Tailand (CIAT)		Sansanayudh 2022
	COOL-AF registry (Cohort of Antithrombotic Use and Optimal INR Level in Patients With Non-Valvular Atrial Fibrillation in Thailand)		Winijkul 2021
	COOL-AF registry (Cohort of Antithrombotic Use and Optimal INR Level in Patients With Non-Valvular Atrial Fibrillation in Thailand)		Krittayaphong 2020
	Thai Type 1 Diabetes and Diabetes Diagnosed Before Age 30 Years Registry, Care and Network (T1DDAR CN)		Dejkhamron 2021
	Thailand Orsiro registry		Suwannasom 2021
	Thai Rheumatic Disease Prior Authorization (RDPA) registry	**Inflammatory and autoimmune disorders**	Narongroeknawin 2018
	Thai Rheumatic Disease Prior Authorization (RDPA) registry		Chiowchanwisawakit 2019

^Out of 9 studies based on clinical registries with cardiology and metabolic disorders (n=7) & infectious diseases and vaccines (n=2), databases name are available in 8 studies while one study does not mention any particular names for databases [cardiology and metabolic disorders].

Unique names of identified clinical registries databases from Thailand (2)

Identified registry databases from oncology & other disease areas from Thailand (N=19^)

Database Type	Database Name	Disease Area	Study Details
**Clinical registry**	Chiang Mai Cancer Registry	**Oncology**	Chitapanarux 2019
	Khon Kaen cancer registry; National Health Security Office		Phimha 2019
	Khon Kaen cancer registry; Khon Kaen Central Hospital TB Database; TB database of the Region 7 Office of Disease Prevention and Control		Nanthanangkul 2020
	Khon Kaen cancer registry		Saenrueang 2019
	Thai Acute Leukemia Working Group (TALWG) registry		Chanswangphuwana 2022
	Thai ALL observational registry		Limvorapitak 2019
	Thai Lymphoma Study Group (TLSG) Registry		Wudhikarn 2020
	Thai Lymphoma Study Group (TLSG) Registry		Wudhikarn 2020
	COOL-AF registry (Cohort of Antithrombotic Use and Optimal INR Level in Patients With Non-Valvular Atrial Fibrillation in Thailand)	**Others**	Krittayaphong 2020
	National diabetes registry		Ruamviboonsuk 2022
	Nationwide registry that collected road traffic injury (RTI) data from all hospitals in Thailand		Riyapan 2018
	Thai 4th National Health Examination Survey; National Civil Registration; Vital Statistics System		Srinonprasert 2018
	Thai PCI registry initiated by the Cardiac Intervention Association of Thailand		Limpijankit 2022
	Thai Spinal Cord Injury Registry		Pattanakuhar 2019
	Thai Transplant Registry		Bruminhent 2020
	Thai Transplant Registry; Thai Red Cross Society		Larpparisuth 2022

^Out of 19 studies based on clinical registries with oncology (n=10) & other disease areas (n=9), databases names are available in 17 studies while two studies do not mention any particular names for databases [oncology (1) & others (1)].

Unique names of other identified databases from Thailand

Identified other database types in studies from multiple disease areas from Thailand (N=13^)

Database Type	Database Name	Disease Area	Study Details
**EMR/EHR; Clinical registry**	Thai Type 1 Diabetes and Diabetes diagnosed Age before 30 years Registry, Care and Network (T1DDAR CN)	**Cardiology and metabolic disorders**	Dejkhamron 2022
	Khon Kaen cancer registry; Khon Kaen Central Hospital; the Region 7 Office of Disease Prevention and Control	**Infectious diseases and vaccines**	Siewchaisakul 2021
	National Health Security Office (NHSO)'s in-patient database; Ministry of Interior civil registration database	**Others**	Sangroongruangsri 2018
**Health insurance/claims**	Nationwide Hospital Admission Data Registry	**Oncology**	Chaiteerakij 2017
	43-files database	**Others**	Barua 2020
	Universal Coverage Health Security Insurance Scheme Database Thailand		Szlachetka 2020
	Universal Coverage Scheme (UCS) claim dataset under the National Health Security Office (NHSO)		Sriratanaban 2020

^Databases names are available for 7 studies based on EMR/EHR; clinical registry (n=3), and health insurance/claims (n=4). **Six studies do not mention any particular names for EMR/EHR databases [Cardiology and metabolic (1), Infectious diseases and vaccines (1), oncology (1), others (3)]**

Abbreviations: EHR, or electronic health records; EMR, electronic medical records
